# A natural depsipeptide antibiotic binds the E-site of the bacterial ribosome

**DOI:** 10.1038/s41586-026-10589-2

**Published:** 2026-06-03

**Authors:** Manpreet Kaur, Dmitrii Y. Travin, Max J. Berger, Manoj Jangra, Martino Morici, Haaris A. Safdari, Dorota Klepacki, Wenliang Wang, Michael Cook, Sommer Chou, Allison K. Guitor, Kalinka Koteva, Min Xu, Linda Ejim, Aline Fiebig, Yeganeh Yousefi, Brian K. Coombes, Lesley Macneil, Nora Vázquez-Laslop, Alexander S. Mankin, Daniel N. Wilson, Gerard D. Wright

**Affiliations:** 1 David Braley Centre for Antibiotics Discovery, McMaster University, Hamilton, Ontario Canada; 2 M. G. DeGroote Institute for Infectious Disease Research, McMaster University, Hamilton, Ontario Canada; 3 Department of Biochemistry and Biomedical Sciences, McMaster University, Hamilton, Ontario Canada; 4 Department of Pharmaceutical Sciences, University of Illinois at Chicago, Chicago, IL USA; 5 Center for Biomolecular Sciences, University of Illinois at Chicago, Chicago, IL USA; 6 Institute for Biochemistry and Molecular Biology, University of Hamburg, Hamburg, Germany

**Keywords:** Antibiotics, Drug discovery, Applied microbiology, Target identification, Structural biology

## Abstract

A key challenge in addressing the antibiotic resistance crisis is identifying new antimicrobial compounds^1^. Although natural products produced by fungi and bacteria, particularly actinomycetes, have been the source of most antibiotics discovered over the past 80 years, they have fallen out of favour owing to the frequent rediscovery of known drug scaffolds^2^. The current perception is that antibiotic-producing actinomycetes have been over-mined and possess little novelty left to yield. Here we demonstrate that by using improved fractionation approaches that enrich previously overlooked minor products, even well-studied strains of antibiotic-producing actinomycetes can provide new chemical scaffolds with unique modes of action. By fractionating a library of natural product extracts from soil bacteria, we show that *Streptomyces rimosus*, the source of the well-known antibiotic oxytetracycline, produces a cyclic depsipeptide antibiotic that we call manikomycin. Manikomycin can kill multidrug-resistant Enterobacteriaceae and is not susceptible to resistance associated with clinically used antibiotics. Biochemical, genetic and structural analyses reveal that manikomycin binds in the E-site of the large subunit of the bacterial ribosome, preventing entry of the 3′ end of the tRNA into the E-site and effectively hindering the translocation step of protein synthesis in a sequence-context-specific manner. Manikomycin is the first antibacterial agent, to our knowledge, to target the critical but underexplored E-site in the large ribosomal subunit, highlighting its value as a lead for developing new antibiotics.

## Abstract (web summary)

Improved fractionation strategies can identify antibiotics with previously unseen scaffolds and mechanisms, exemplified by manikomycin from *Streptomyces rimosus*, which acts by targeting the E-site of the bacterial large ribosomal subunit.

## Main

Microbial natural products, particularly those derived from actinomycetes, have been a dominant source of antibacterial agents over the past 80 years. These antibiotics were identified by testing the ability of crude extracts of their producers to inhibit bacterial growth, a strategy termed the Waksman platform after microbiologist Selman Waksman, who pioneered this approach^1^. Despite the initial spectacular success of that approach, its utility has decreased over the years, owing to the frequent re-isolation of common chemical scaffolds and the shift toward target-based high-throughput screens of readily sourced synthetic compounds^2,3^. However, the rise of multidrug-resistant pathogens combined with the limited success of target-based screens and synthetic chemical libraries in antibiotic drug discovery has reignited interest in microbial natural products as sources of new antimicrobial agents. The growing realization of the untapped genomic potential in actinomycetes has fuelled a renewed interest in these microbes as sources of new drug leads^4^.

*Streptomyces* genomes show an abundance of biosynthetic gene clusters (BGCs) that potentially encode antibacterial compounds. However, only a small fraction of such compounds have been isolated, owing to the often low levels of BGC expression, dominance of one of the several produced antibacterial compounds, and insufficiently discriminating analytical approaches. One strategy to tap into the cryptic antibiotic pool is the improved fractionation of natural product extracts^5^, enabling the separation of components with overlapping activities, such as two unrelated antibiotics^6^. Here we report the application of this strategy to discover a new antibiotic with a previously unknown mode of action. We identified a novel cyclic depsipeptide antibiotic, manikomycin (MKM), derived from *Streptomyces rimosus*, which has been known since 1950 as a producer of the well-known antibiotic oxytetracycline^7^. We show that MKM exhibits a unique mechanism of action: it binds to the E-site of the large subunit of the bacterial ribosome and interferes with translocation in a context-specific manner. Additionally, we found that MKM is effective against drug-resistant Gram-negative pathogens and is unaffected by resistance mechanisms found in bacterial clinical strains, thereby offering a new chemical scaffold for antibiotic development.

## Identification of MKM

**Extended Data Fig. 1:**
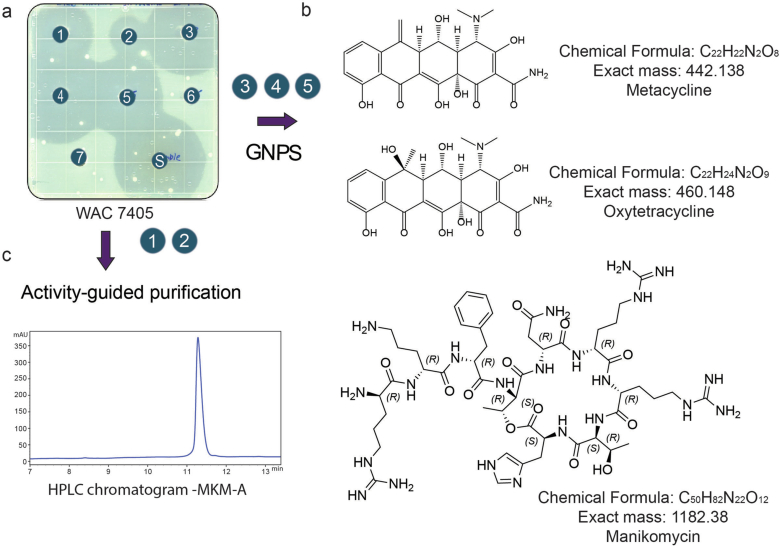
Activity and dereplication strategy of PFL fractions from *Streptomyces rimosus* WAC 7405. **a**, Activity of WAC 7405 fractions obtained through LH-20 chromatography, which separates primarily by size (where 1-7 are the fractions collected, and S stands for the activity of sample loaded on the column with the indicator strain *E. coli* BW25113 Δ*tolC*Δ*bamB*). Fractions were dereplicated through GNPS analysis. The activity is reduced in fraction 2 and increased again in fraction 3-5 suggesting the presence of two compounds. **b**, Larger ions were identified in fractions 1 and 2, while oxytetracycline was detected in fractions 3-5. **c**, Activity-guided purification from fractions 1 and 2 led to the discovery of MKMs as shown in the HPLC chromatogram of MKM-A.

In search of new antibiotics, we screened a collection of methanolic extracts from 255 bacterial strains from our in-house Wright Actinomycetes Collection (WAC) library^6^, consisting primarily of diverse actinomycetes isolated from various soil samples. The WAC library was assembled to maximize chemical novelty, and extracts were pre-fractionated to separate compounds on the basis of polarity, thereby increasing the likelihood of identifying bioactive hits compared with crude extracts. Hit fractions were then subjected to a metabolomics-guided dereplication to minimize the rediscovery of known natural products. Using this library, we screened for growth inhibition of the Gram-negative antibiotic-hypersensitive *Escherichia coli* BW25113 *ΔtolC*Δ*bamB*. Further fractionation by size-exclusion chromatography of the extract from *S. rimosus*, strain WAC 7405, yielded differentially eluting fractions with antibiotic activity (Extended Data Fig. 1a). Global Natural Products Social (GNPS)^8^ molecular networking analysis of the mass spectral signatures, which compares tandem mass spectrometry (MS/MS) fragmentation patterns to group related molecules and quickly recognize known compounds through public spectral libraries, identified oxytetracycline and metacycline in fractions 3, 4 and 5 (Extended Data Fig. 1b). By contrast, no matches to known compounds were found in active fractions 1 and 2 (Extended Data Fig. 1c). Subsequent bioactivity-guided purification of the active compounds from fractions 1 and 2 identified the MKMs, a family of previously unknown cationic cyclic depsipeptides. To further check their novelty beyond GNPS, we performed independent structure-based searches in SciFinder and PubChem; neither database contained any molecules that matched or closely resembled the MKM scaffold.

The compound was named from the Hindi and Punjabi word *manik* (meaning precious gem), reflective of its rarity and unique mode of action. The most abundant was the nonapeptide MKM-A. The chemical structure of MKMs was determined using a combination of mass spectrometry and 1D/2D NMR spectroscopy (Supplementary Figs. 1–22 and Supplementary Tables 1–3) and the stereochemistry of the amino acids was verified by Marfey’s analysis (Supplementary Fig. 23). Less abundant MKM variants included the nonapeptide MKM-B, the octapeptides MKM-C and MKM-D, and the decapeptide MKM-E (Fig. 1a and Supplementary Figs. 24 and 25). MKMs are depsipeptides that are cyclized through an ester linkage between carboxyl of the C-terminal His and the side-chain hydroxyl group of Thr4 (MKM-A numbering) (Fig. 1a). MKM-A, MKM-D and MKM-E incorporate a d-ornithine (d-Orn) residue at position 2 (MKM-A numbering), whereas MKM-B and MKM-C have d-Arg in this position (Fig. 1a).

**Fig. 1:**
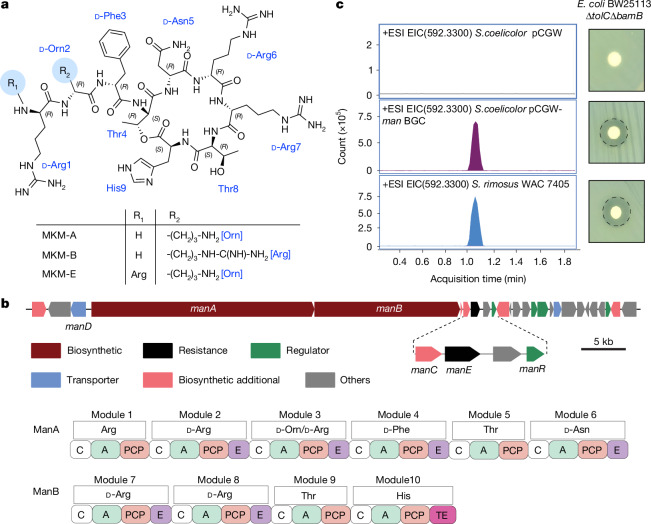
Identification of the cyclic depsipeptide MKM produced by *S. rimosus.* **a**, Chemical structure of MKMs. The table represents the substitutions in the various isoforms. Numbering of residues reflects MKM-A structure. **b**, Top, *man* BCG, the BGC responsible for MKM production. The predicted functions of selected encoded proteins are listed. Bottom, the modular structure of the NRPSs ManA and ManB. A, adenylation domain; C, condensation domain; PCP, peptidyl carrier protein; E, epimerization domain; TE, thioesterase domain. **c**, Liquid chromatography–mass spectrometry analysis and bioactivity of partially purified extracts of MKM conjugants obtained from the heterologous expression of the MKM BGC in *S. coelicolor*. Top, control strain carrying the empty plasmid pCGW. Middle, *man* BGC expressed from the pCGW plasmid. Bottom, wild-type *S. rimosus* WAC 7405. The doubly charged species of MKM-A observed at 592.33 [M+2H]^2+^ is highlighted in colour. The mass spectrum is shown in Supplementary Fig. 34b. The panels on the right display bioactivity assays, in which extracts from exconjugants were spotted on a lawn of the indicator strain *E. coli* BW25113 Δ*tolC*Δ*bamB*. The growth-inhibition zones are outlined with black dashed circles.

**Extended Data Fig. 2:**
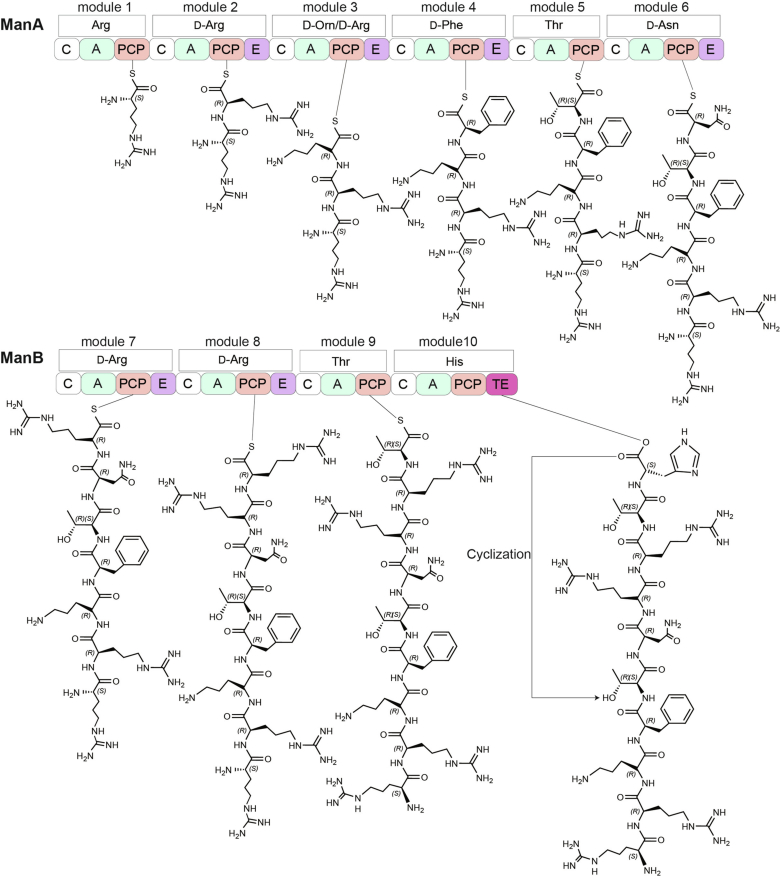
Proposed biosynthesis pathway of MKM. MKM biosynthesis involves NRPSs, ManA and ManB, containing 10 amino acid modules, and encoding six epimerization domains, with the L or D stereochemistry of each amino acid specified. The thioesterase (TE), responsible for ring closure between histidine and threonine, is shown on the right. Once synthesized, the peptide is exported via the ManD transporter. Domain abbreviations: **A**, adenylation domain; **C**, condensation domain; **PCP**, peptidyl carrier protein; **E**, epimerization domain; **TE**, thioesterase domain.

Sequencing of the *S. rimosus* WAC 7405 genome, followed by antiSMASH analysis, identified *man* BCG, the putative BGC responsible for the production of MKMs (Fig. 1b and Supplementary Table 5). The BGC encompasses the genes encoding the non-ribosomal peptide synthetases (NRPSs) ManA and ManB, which consist of six and four modules, respectively, and are collectively capable of incorporating ten amino acids into the MKM scaffold. The organization of ManA and ManB modules corresponds to the amino acid composition observed in the MKM-E variant. The final step in MKM biosynthesis involves the macrocyclization between the C-terminal His9 and Thr4 and release from the NRPS complex, which is likely to be catalysed by a thioesterase domain encoded by the C-terminal final module^9^ (Extended Data Fig. 2). The stereochemistry predicted on the basis of the presence or absence of epimerase domains accurately aligned with experimental data for all ten amino acids (Supplementary Figs. 23 and 24).

We transferred the entire 67 kb MKM BGC into the heterologous host *Streptomyces coelicolor* M1154 using transformation-associated recombination cloning^10^. The resulting strain successfully secreted MKMs, as confirmed by mass spectrometry and antibacterial activity assays (Fig. 1c and Supplementary Fig. 34b), validating the assignment of the MKM BGC in *Streptomyces* sp. WAC 7405. MKM-A (hereafter referred to simply as MKM) was used in further studies unless mentioned specifically.

## MKM inhibits bacterial translation

**Fig. 2:**
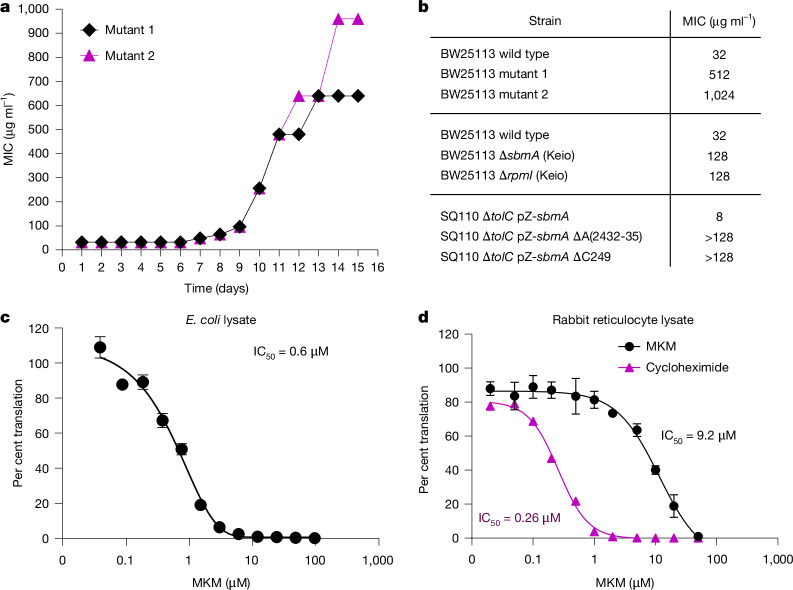
MKM targets bacterial protein synthesis. **a**, Acquisition of resistance during serial passaging in the presence of sub-MIC concentrations of MKM. The *y* axis represents the highest concentration at which cells continued to grow during passaging**. b**, MIC values for the MKM-resistant mutants and corresponding wild-type strains (*E. coli* BW25113, *E. coli* Δ*sbmA*, Δ*rpmI* and *E. coli* strain SQ110∆*tolC* pZ-*sbmA*). **c**, MKM inhibits protein synthesis in a *E. coli* cell-free transcription–translation system programmed with firefly luciferase-encoding plasmid. Per cent translation represents residual luminescence compared with the untreated control. Data are mean ± s.d. of three technical replicates and are representative of two biological replicates with similar results. **d**, Inhibition of protein synthesis by MKM in rabbit reticulocyte lysate programmed with luciferase mRNA. Cycloheximide was used as a positive control. Data for MKM are mean ± s.d. of three independent replicates.

**Extended Data Fig. 3:**
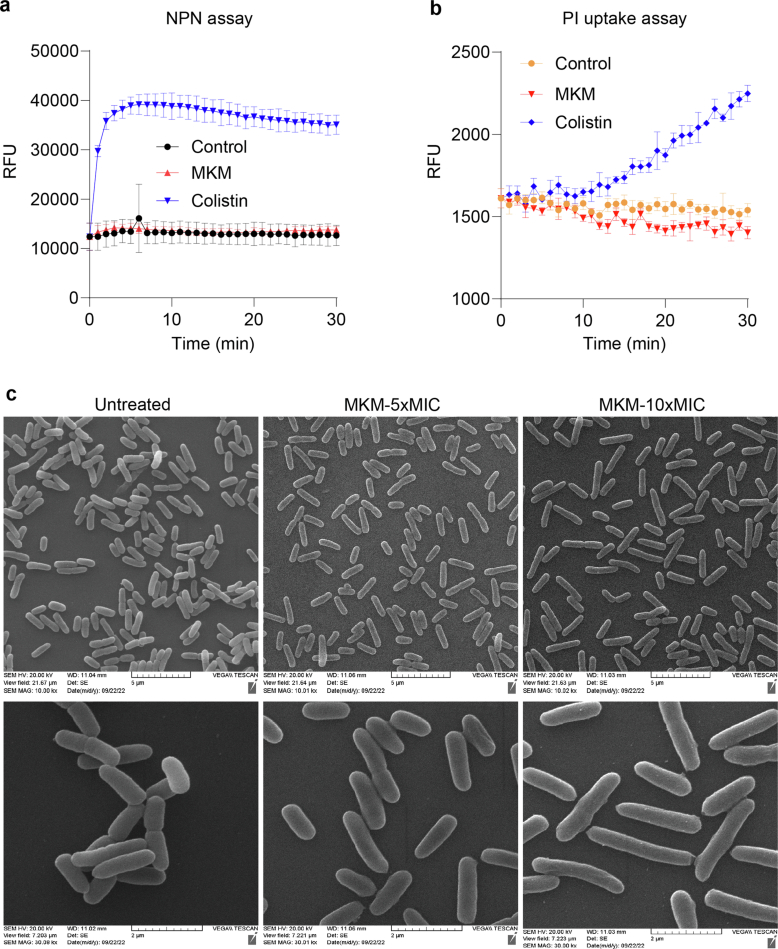
Effect of MKM on *E. coli* membrane integrity and morphology. **a**, Outer membrane permeabilization assay using NPN dye in *E. coli* BW25113 cells. The y-axis shows relative fluorescence units (RFU). Controls include untreated cells and colistin (5 µg/ml) as a positive control for outer membrane disruption. MKM was tested at 10× MIC (10 µg/ml). **b**, Inner membrane disruption assay using propidium iodide (PI) in *E. coli* BW25113 cells. The y-axis indicates RFU corresponding to DNA-bound PI. MKM and colistin were tested at the same concentrations as in (a). Data in (a) and (b) are presented as mean ± SD of three biological replicates. **c**, Scanning electron microscopy (SEM) analysis of *E. coli* BW25113 treated with MKM at 5× and 10× MIC. The upper panels (magnification: 10,000×, scale bar: 5 µm) and lower panels (magnification: 30,008×, scale bar: 2 µm) display different magnifications of the same sample. The images are representative of two independent experiments.

The abundance of positively charged residues in MKM could suggest membranolytic activity, similar to other cationic antimicrobial peptides. However, MKM did not permeabilize or disrupt the bacterial membrane (Extended Data Fig. 3a,b), and no detectable changes in bacterial cell morphology were observed that would indicate membrane restructuring (Extended Data Fig. 3c). Therefore, we shifted our focus to the possibility that MKM acts on an intracellular target. Passaging of *E. coli* strain BW25113 (ref. ^11^) in the presence of subinhibitory concentrations of MKM led to the selection of highly resistant MKM mutants with minimum inhibitory concentrations (MICs) of 512–1,024 µg ml^−1^, exceeding the MIC of the parental strain by 16- to 32-fold (Fig. 2a,b). The resistance frequencies for wild-type strains were as low as 3.7 × 10^−10^ for *E. coli* BW25113 and 1.1 × 10^−8^ for *Klebsiella* *pneumoniae* C1559. Whole-genome sequencing of one of the resistant mutants identified, among other changes, loss-of-function mutations in the gene *sbmA* encoding an inner membrane peptide transporter involved in the uptake of other peptide antibiotics^12–14^ (Supplementary Table 6). In general agreement with this finding, the *∆sbmA* strain from the *E. coli* single-gene knockout Keio collection^15^ exhibited a fourfold increase in MIC (Fig. 2b). Deletion of the genes encoding the components of YejABEF, another transporter that also internalizes peptide antibiotics^16,17^, similarly resulted in a twofold to eightfold increase in MIC (Supplementary Fig. 36a,b), indicating that MKM, unlike some other antibiotics whose uptake depends on a single transporter^18^, exploits several pathways to enter the cell and is likely to act on an intracellular target.

Sequencing of the genome of another selected MKM-resistant mutant showed the presence of a nonsense mutation in the *rpmI* gene encoding the large ribosomal subunit protein bL35 (Supplementary Table 6). This finding pointed to the ribosome as a possible cellular target of MKM. Most ribosome-targeting antibiotics interact with ribosomal RNA (rRNA)^19^; however, because of the redundancy of rRNA genes in bacteria such as *E. coli*, rRNA resistance mutations are extremely rare. Therefore, to verify that MKM interferes with bacterial growth by inhibiting the ribosome, we selected MKM-resistance mutants in the antibiotic-hypersusceptible *E. coli* strain SQ110∆*tolC* pZ-*sbmA* that carries a single copy of the rRNA operon on its chromosome and overexpresses the MKM-internalizing transporter^20,21^. Several hundred resistant colonies appeared with a frequency of about 10^−7^ when cells were plated on agar supplemented with 4× MIC of MKM. Sequencing the 23S rRNA gene in 11 randomly picked MKM-resistant mutants revealed a deletion of an adenine in the 4-adenine stretch (A2432–A2435) in 10 of these mutants and a deletion of C249 in the remaining mutant. All these mutants showed more than 16-fold increase in MKM MIC (Fig. 2b). Of note, these mutations, as well as the bL35 protein, are located near the E-site of the bacterial large ribosomal subunit, a location that has never been previously identified as a binding site for antibacterial compounds^22^.

Using an *E. coli* cell-free translation system, we confirmed that MKM is a potent inhibitor of bacterial protein synthesis (half-maximal inhibitor concentration (IC_50_) = 0.6 µM) (Fig. 2c). By contrast, MKM acted as a much weaker inhibitor of protein synthesis in a rabbit reticulocyte lysate translation assay, exhibiting IC_50_ of 9.2 µM, approximately 15-fold higher than that for the bacterial system and 35-fold higher than that of cycloheximide (Fig. 2d), a known inhibitor of eukaryotic translation at the E-site^23^. These results reveal MKM as a selective antibacterial compound targeting protein synthesis.

## MKMs bind to the E-site of the 50S ribosome

**Fig. 3:**
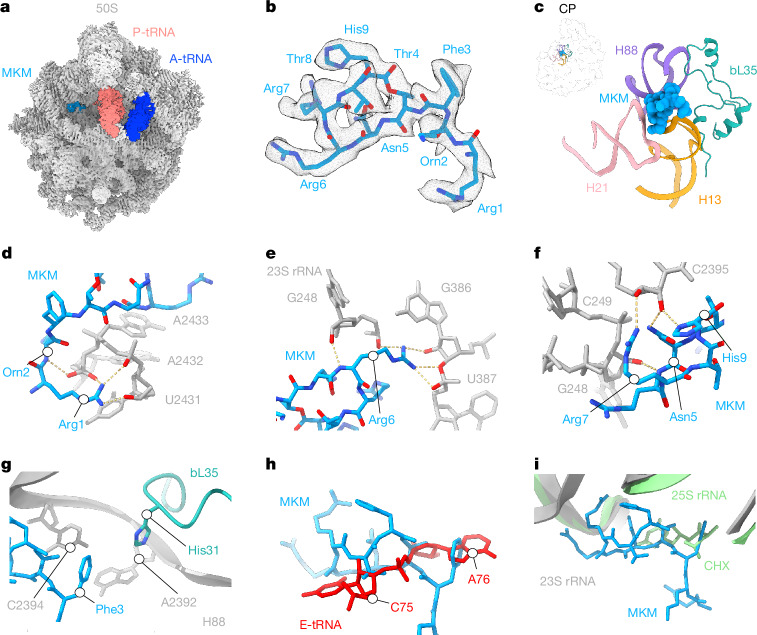
MKM binds within the E-site of the *E. coli* 50S subunit. **a**, Cryo-EM density map of the 50S subunit with A-tRNA, P-tRNA and MKM highlighted. **b**, Cryo-EM density (grey mesh) and molecular model for MKM (blue). **c**, Binding site of MKM on the 50S subunit, encompassing H13, H21 and H88 of the 23S rRNA and ribosomal protein bL35. Inset shows the orientation of MKM-binding site on the 50S subunit (transparent grey), with central protuberance (CP) labelled. **d**–**f**, Potential hydrogen-bond interactions (dashed yellow line) between MKM and nucleotides of the 23S rRNA (light grey). **g**, Ribosomal protein bL35 does not directly interact with MKM (blue), but directly interacts with 23S rRNA nucleotides located within H88. **h**, Overlay of the binding position of MKM with the modelled E-tRNA showing clash with A76 and C75 of the CCA-end of the E-tRNA (Protein Data Bank (PDB) ID 8AKN)^28^. **i**, Overlay of the binding position of MKM on the *E. coli* 70S ribosome (23S rRNA) with cycloheximide (CHX) bound to the eukaryotic yeast 80S ribosome (25S rRNA) (PDB ID 4U3U)^29^.

**Extended Data Fig. 4:**
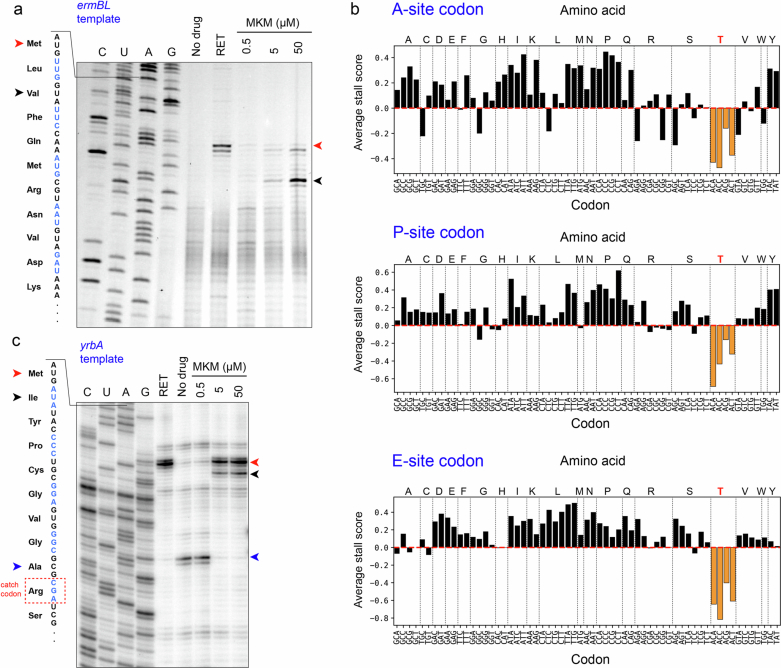
Mechanism of MKM action. **a**, Toeprinting analysis of MKM effect on translation of *ermBL*. Red and black arrowhead indicates the sites of MKM-induced translation arrest at the start and internal codons, respectively. **b**, Codon-specificity of MKM-induced translation arrest. Average stall-score is calculated for each of the 61 codons in the three functional sites of the ribosome based on the Ribo-seq data. **c**, Toeprinting analysis of the MKM effect on translation of the modified *yrbA* template. Blue arrowhead indicates ribosome stalling at the Ala codon preceding the Arg 10 “catch codon” due to the presence of Arg-RS inhibitor (Arg-AMS) in all reaction mixture. The start codon toeprinting band is indicated by a red arrowhead and the other MKM-induced stalls are marked by black arrowheads. Retapamulin (RET), known to stall ribosomes at start codons^56^, was used as a control. Gels shown in panels a and c are representative of two independent experiments with similar outcomes and their raw blots are shown in Supplementary Fig. 39.

We used high-resolution structural analysis to determine the binding site of MKMs on the translating ribosome. First, by using toeprinting, a technique that enables mapping of the position of a drug-arrested ribosome on mRNA^24,25^, we showed that the ribosomes stall primarily on the third codon of the model *ermBL* mRNA translated in an *E. coli* in vitro protein synthesis system in the presence of MKM (Extended Data Fig. 4a). We then analysed the resulting MKM-stalled ribosome complexes (MKM-SRC) using single particle cryogenic electron microscopy (cryo-EM), as we have done previously for other antibiotic-SRCs^26,27^. In silico sorting of the cryo-EM data revealed one major functional state of the ribosome containing A- and P-tRNAs, but no E-tRNA (Supplementary Fig. 26), which could be refined to 2.5 Å resolution (Extended Data Table 2 and Supplementary Fig. 27), and further improved to 2.4 Å by focused refinement on the 50S subunit (Fig. 3a, Extended Data Table 2 and Supplementary Fig. 27). After modelling of the 70S ribosome, as well as of A- and P-site tRNAs, two additional cryo-EM densities remained that could be unambiguously assigned to MKM. One MKM-binding site (which we refer to as the primary site) is located within the E-site of the 50S subunit (Fig. 3a–c). The second binding site is at the back of the 50S subunit (Supplementary Fig. 28), far from any known ribosomal functional centres. The de novo modelled conformation of MKM confirms that this cyclic peptide is formed by linkage of the side-chain hydroxyl of Thr4 with the backbone carboxyl of His9 and contains a linear N-terminal tail consisting of Arg1, Orn2 and Phe3 (Fig. 3b and Supplementary Fig. 28). In the primary binding site, the core ring of MKM inserts into a pocket formed by the tips of the 23S rRNA helices H13 and H21 in domain I and nucleotides at the base of H88 in domain V (Fig. 3c). Many direct hydrogen-bonding interactions are formed between the charged residues of MKM (Arg1, Orn2 within the N-terminal tail and Asn5, Arg6, Arg7 and His9 in the ring), predominantly with the sugar-phosphate backbone of 23S rRNA nucleotides, including G248–C249 (H13), G386 (H21), C2395 and U2431–A2433 (H88) (Fig. 3d–f and Supplementary Video 1). Additionally, a network of water-mediated interactions is observed between Asn5 and Arg7 of MKM with G248 and C249 within H13 (Supplementary Fig. 29 and Supplementary Video 1). Consistent with this site's being the primary point of MKM action, the locations of the rRNA resistance mutations (deletion of C249, or of an adenine within the A2432–A2435 stretch) are in close proximity to the bound antibiotic. These deletion mutations likely confer resistance by inducing local conformational changes in the 23S rRNA that disrupt the interactions observed with MKM (Fig. 3d–f and Supplementary Fig. 29). Protein bL35, whose truncation by a nonsense mutation confers resistance, is also located close to this primary MKM-binding site but does not directly contact the antibiotic. Instead, bL35 establishes multiple interactions with H13 and H88 (Fig. 3c,g) and therefore, MKM resistance resulting from the bL35 gene mutation probably arises owing to allosteric perturbations in the MKM-binding site via conformational changes in the 23S rRNA (Fig. 3g).

**Extended Data Fig. 5:**
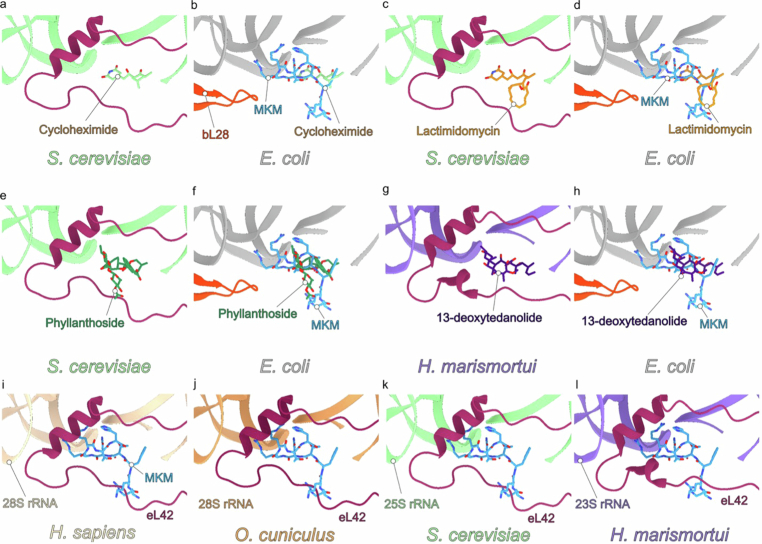
Binding position of MKM relative to eukaryotic-specific translation inhibitors and eukaryotic ribosomes. **a-f**, comparison of the Yeast/*S. cerevisiae* 80S ribosome bound with (**a**) cycloheximide (green, PDB ID 4U3U)^29^, (**c**) lactimidomycin (orange, PDB ID 4U4R)^29^ and (**e**) phyllanthoside (dark green, PDB ID 4U4Z)^29^ alone, or (**b,d,f**) overlaid with the position of MKM (blue) from the *E. coli* MKM-70S structure determined here. **g-h**, comparison of the archaeal/*H. marismortui* 70S ribosome bound with (**g**) 13-deoxytedanolide (purple, PDB ID 2OTJ)^31^ alone, or (**h**) overlaid with the position of MKM (blue) from the *E. coli* MKM-70S structure determined here. Structural alignments based on large subunit rRNAs. **i-l**, Binding position of MKM (blue) superimposed onto the E-site of (**a-c**) eukaryotic ribosomes including (**i**) Humans/*Homo sapiens* (PDB ID 8GLP)^30^, (**j**) Rabbit/*Oryctolagus cuniculus* (PDB ID 6SGC)^102^, and (**k**) Yeast/*Saccharomyces cerevisiae* (PDB ID 4U3U)^29^ and the archaeal 70S ribosome from (**l**) *Haloarcula marismortui* (PDB ID 2OTJ)^31^.

Bound in its primary site, MKM obstructs placement of C75 and A76 of the CCA end of a deacylated tRNA within the E-site of the large ribosomal subunit^28^, with the greatest overlap of Phe3 of MKM with the 3′ terminal adenine of the E-tRNA (Fig. 3h). Notably, the E-site of the large subunit of archaeal and eukaryotic ribosomes is the target of several translation inhibitors, such as cycloheximide (Fig. 3i), lactimidomycin, 13-deoxytedanolide and phyllanthoside^29–31^, that exhibit overlapping but distinct binding modes compared to MKM (Extended Data Fig. 5a–h) but do not inhibit bacterial translation^32,33^. A comparison of the primary MKM-binding site with eukaryotic ribosomes^29,30^ shows that the presence of an archaeal/eukaryotic-specific ribosomal protein, eL42, would sterically block binding of MKM to the E-site (Extended Data Fig. 5i–l). This explains the low toxicity of MKM against human cell lines (Extended Data Table 1) and poor inhibition by MKM of eukaryotic in vitro translation (Fig. 2d).

## Mechanism of translation inhibition by MKM

**Fig. 4:**
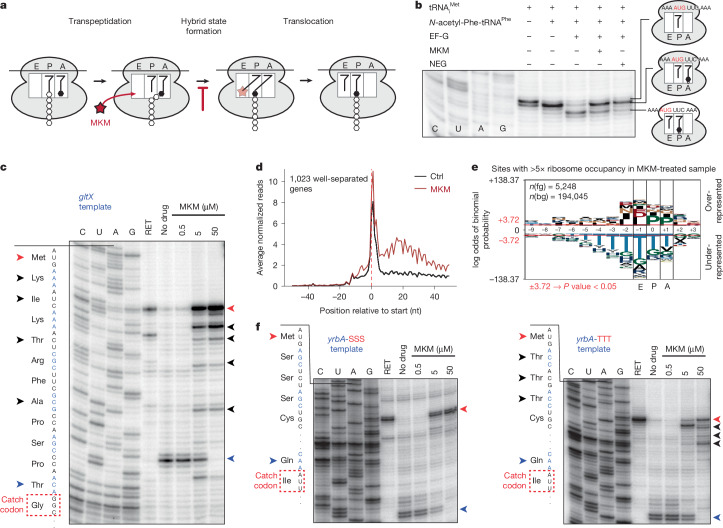
Mechanism of translation inhibition by MKM. **a**, The general scheme of translocation. Binding of MKM in the large subunit E-site would prevent the ribosome hybrid state formation, thereby interfering with translocation. **b**, Translocation assay. NEG, negamycin. **c**, Toeprinting analysis of the effect of MKM on *gltX* translation. Blue arrowhead indicates ribosome stalling at the Thr 12 codon preceding the Gly 13 ‘catch codon’ due to the presence of Gly-RS inhibitor (Gly-AMS) in all the reactions. The start codon toeprinting band is indicated by a red arrowhead, and the other MKM-induced stalls are marked by black arrowheads. Retapamulin (RET), known to cause translation arrest at start codons^56^, was used as a control. **d**, Metagene analysis of ribosome density around the start codons of the genes in MKM-treated (red line) or control (Ctrl) untreated (black line) cells deduced from the results of ribosome profiling. **e**, pLogo analysis of codons specifying individual amino acids at the sites of preferential MKM-induced ribosome stalling. The red horizontal lines correspond to *P* = 0.05 (ref. ^57^). **f**, Toeprinting analysis of MKM-induced ribosome stalling during translation of the *yrbA* template carrying the insertion of three Thr codons (right) or three Ser codons (left) after the start codon of the gene. Red and black arrowheads indicate positions of the ribosomes stalled at the start codon or internal codons of the gene, respectively. Blue arrowhead indicates ribosome stalling in front of the Ile 13 codon due to the presence of the Ile-RS inhibitor mupirocin in the reaction mixture. Gels shown in panels **b**,**c**,**f** are representative of at least two independent experiments with similar outcomes; raw blots are shown in Supplementary Fig. 37.

The overlap in the binding site of MKM with the CCA end of the E-site tRNA (Fig. 3h) suggests that MKM would impede the formation of the ribosome hybrid state eventually preventing translocation of the P-tRNA into the E-site (and, therefore, of A-tRNA into the P-site) (Fig. 4a). Consistent with that, the majority of MKM-SRCs visualized in the cryo-EM reconstructions were in the pre-translocation state containing A- and P-site tRNAs, whereas no hybrid states (A/P-tRNAs and P/E-tRNAs) or post-translocation states (P-tRNAs and E-tRNAs) were detected (Supplementary Fig. 26). To experimentally verify the mode of MKM action, we directly tested the ability of MKM to interfere with translocation in vitro (Fig. 4b). To do this, we generated a pre-translocation complex by binding deacylated tRNA_i_^Met^ in the P-site and *N*-acetyl-Phe-tRNA^Phe^ in the A-site of the *E. coli* ribosome. After the addition of elongation factor G (EF-G), in the absence of antibiotics, the ribosome readily translocated to the next mRNA codon. This reaction, however, was notably inhibited by MKM or the antibiotic negamycin, a known translocation inhibitor^34^.

Our original toeprinting experiments showed that MKM stalled the ribosome on the third codon of the *ermBL* template encoding a protein with the N-terminal sequence MLVFQ (Extended Data Fig. 4a). Consistent with that, careful analysis of the cryo-EM structure of the ribosome stalled by MKM on the *ermBL* mRNA revealed the P-tRNAs and A-tRNAs as being tRNA^Val^ and tRNA^Phe^, respectively. However, a universal inhibitor of translocation added to an in vitro translation reaction prior to its onset is expected to arrest the ribosome at the start codon (Supplementary Fig. 30). Motivated by this apparent contradiction, we used toeprinting to examine the effect of MKM on translation of a template encoding the early codons of the *E. coli* gene *gltX* (Fig. 4c). Whereas MKM arrested some ribosomes at the start codon, a notable fraction of them became arrested at several specific sites along the mRNA, suggesting that the inhibitory action of MKM on translation elongation (probably by interfering with translocation; Fig. 4b), is influenced by the sequence context of the mRNA or the synthesized protein.

To gain further insights into the sequence specificity of MKM action, we analysed its effect on translation in *E. coli* cells by ribosome profiling, a technique that can reveal the redistribution of ribosomes through the length of mRNAs in response to antibiotic treatment^35,36^. Metagene analysis showed increased ribosome occupancy at the early mRNA codons in the cells exposed to MKM (Fig. 4d), an effect that is commonly observed with elongation-inhibiting antibiotics^37–39^. Analysis of the sites of preferential MKM-induced ribosome stalling showed enrichment of Pro codons in the P- and A-sites of the drug-arrested ribosomes (Fig. 4e). Because incorporation of proline residues into proteins is generally problematic^40–42^, it is possible that the resulting transient idling of a ribosome translating Pro codons offers more time for the E-site tRNA to dissociate prior to the subsequent translocation, thereby increasing the accessibility of the vacant E-site for MKM binding. We also noted increased presence of specific amino acid residues in the three C-terminal positions of the nascent peptide at the preferential sites of MKM action (most notably Ile, Leu and Pro), suggesting nascent polypeptide-dependent ribosome pausing that could also facilitate MKM binding. Notably, the strongest effect was the depletion of Thr codons at the sites of MKM-induced arrest and over several preceding codons (Fig. 4e and Extended Data Fig. 4b). This result suggests that arrest at Thr codons is the least efficient compared to that at other codons. To experimentally verify this observation, we modified the *yrbA*-derived template (Extended Data Fig. 4c) used in our previous studies^43^ by introducing either three Thr codons (*yrbA*-TTT mRNA) or, as a control, three Ser codons (that did not show enrichment or depletion at the sites of MKM stalling) (*yrbA*-SSS) after the mRNA start codon. In agreement with the observations drawn from ribosome profiling, at high concentrations of MKM no ribosomes pass downstream of the start codon of the *yrbA*-SSS template (Fig. 4f, left). By contrast, despite the presence of MKM, a ribosome was able to move, albeit inefficiently, through the Thr codons of the *yrbA*-TTT mRNA (Fig. 4f, right). We envision that either the structural properties of tRNA^Thr^ or peculiarities of its translocation may account for some aspects of the context specificity of the MKM action.

## Mechanisms of MKM self-resistance in *S. rimosus*

**Fig. 5:**
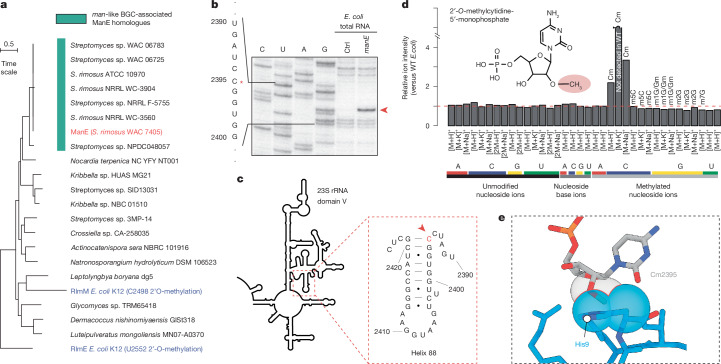
ManE methyltransferase confers resistance to MKM by modification of 2′-OH group of C2395 in 23S rRNA. **a**, Phylogenetic tree of ManE homologues. Close homologues found in *man*-like BGCs are highlighted in green. Distant homologues that are not associated with any BGC are also shown. *E. coli* RlmE served as the outgroup. **b**, Primer extension analysis of 23S rRNA modification. Note the appearance of a band reflecting the modification of C2395 for an RNA sample from ManE-expressing, but not control *E. coli*. The gel is representative of two independent experiments with similar outcomes. The raw blot is shown in Supplementary Fig. 38. **c**, Location of C2395 in the 2D structure of *E. coli* 23S rRNA. **d**, Integrated ion intensities for individual ions, including nucleosides, nucleobases resulting from in-source fragmentation and methylated nucleosides, expressed as a ratio relative to the corresponding ions in wild-type (WT) strains. Ions are annotated by the molecular position of methylation, determined by the retention time of individual standards. The chemical structure of 2′-*O*-methylcytidine-5′-monophosphate is shown as an inset. **e**, Modelling of the 2′-*O*-methyl group of Cm2395 shows that it sterically clashes with the side chain of His9 in MKM.

**Extended Data Fig. 6:**
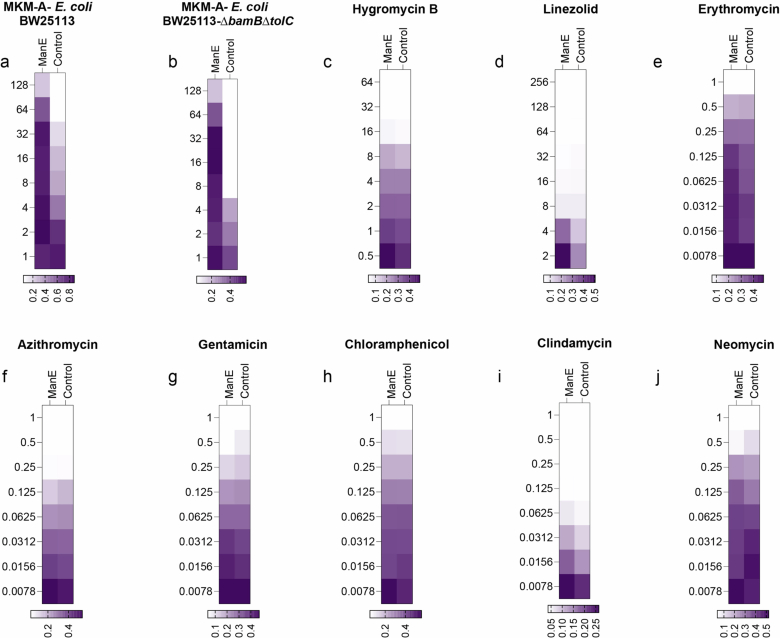
ManE confers specific resistance to MKM, but not to other tested translation inhibitors. **a,b**, Heat maps of MIC of *manE*-expressing strain compared to control strains with MKM-A. **c-j**, Heat maps of MIC of various translation inhibitors against *manE*-expressing strains. The data are displayed as a heatmap, where darker colours indicate relative growth based on OD_600_ values, as shown in the scale bar.

To avoid self-toxicity, bacterial antibiotic producers must be immune to the action of the inhibitor that they synthesize. Such immunity to ribosome-targeting antibiotics is often conferred by methylation of specific rRNA residues located at the sites of the action of the compound^44–46^. Analysing genes associated with the MKM BGC, we identified a putative rRNA methyltransferase gene, *manE* (Fig. 1b), which we hypothesized might encode a self-resistance protein. Notably, the *manE* gene is found exclusively in the strains of *S. rimosus* whose genomes encode MKM BGCs, underscoring its relevance for MKM production and action (Fig. 5a and Supplementary Fig. 31). To investigate the putative site of ManE action and its role in MKM resistance, we expressed the *manE* gene in *E. coli*. ManE expression conferred resistance to MKM (an increase of more than 32-fold in MIC for both *E. coli* BW25113 and *E. coli* BW25113 Δ*tolC*Δ*bamB*) (Extended Data Fig. 6a,b), but not to other translation inhibitors (Extended Data Fig. 6c–j), suggesting that ManE modifies rRNA within the site of MKM action. Primer extension on rRNA isolated from wild-type or ManE-expressing *E. coli* was used to identify the site(s) of ManE-installed rRNA modification. A cDNA band corresponding to the reverse transcriptase pausing in front of C2395 in ManE-modified 23S rRNA suggested the presence of a ManE-catalysed posttranscriptional modification of this nucleotide (Fig. 5b,c). To verify the nature of the modification, the 23S rRNA from wild-type and ManE-expressing *E. coli* cells was isolated, digested to nucleosides and analysed by hydrophilic interaction liquid chromatography–mass spectrometry (HILIC–MS)^47^. The specific ions present at increased abundance in the ManE-expressing strain were consistent with methylation of the 2′-OH of the cytidine ribose (Cm) (Fig. 5d). Notably, in our structure, the 2′-OH of C2395 directly interacts with MKM in its primary binding site, and methylation of the 2′-OH of C2395 would preclude the formation of this hydrogen bond with MKM, thus providing a structural explanation for how methylation confers MKM resistance (Fig. 5e).

## Antimicrobial efficacy of MKMs

**Fig. 6:**
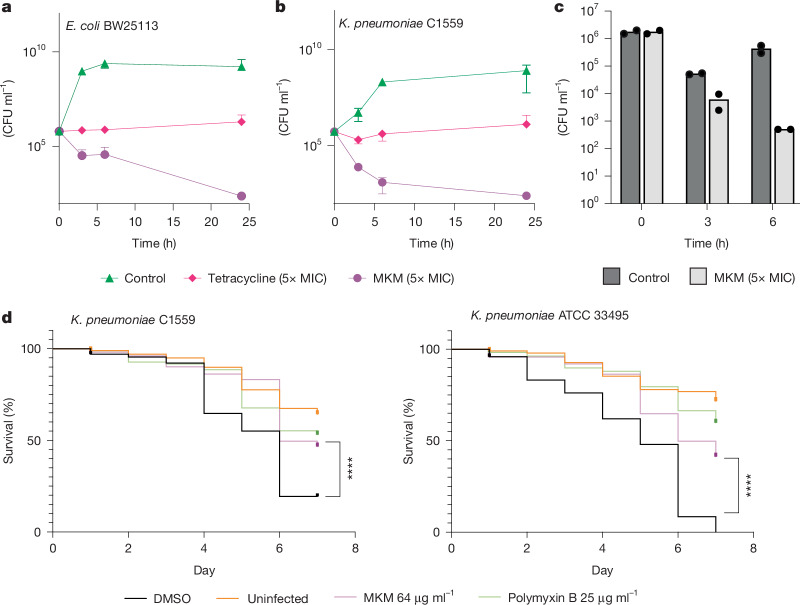
Antimicrobial activity of MKM. **a**,**b**, In vitro time-kill assay in Mueller–Hinton broth medium showing the bactericidal effect of MKM against *E. coli* BW25113 (**a**) and *K. pneumoniae* C1559 (**b**), with tetracycline as a positive control. CFU, colony-forming units. Data are mean of three biological replicates ± s.d. **c**, Effect of MKM in an ex vivo human blood model. *K. pneumoniae* C1559 was inoculated into human blood, and growth was assessed after 3 and 6 h of treatment with MKM. Data are mean of two biological replicates, with black circles indicating individual data points for each experiment. **d**, Survival curves of *C. elegans* infected with *K. pneumoniae* C1559 (left) or *K. pneumoniae* ATCC 33495 (right) and treated with MKM (64 µg ml^−1^; pink) or polymyxin B (25 µg ml^−1^; green) for 6 days. DMSO-treated infected worms (black) showed the lowest survival, whereas MKM and polymyxin B significantly improved survival compared with the DMSO group. Uninfected worms (orange) served as healthy controls. Each curve represents a minimum of three biological replicates (*n* > 60 worms per group). Survival was monitored daily. Statistical significance was determined using the log-rank (Mantel–Cox) test (****P < 0.0001). Source data

**Extended Data Fig. 7:**
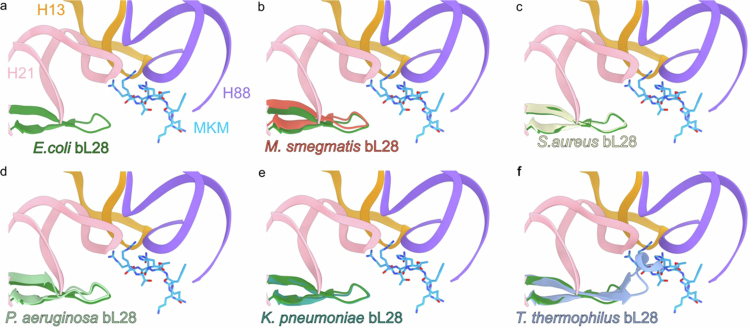
Comparison of the binding site of MKM on different bacterial ribosomes. **a**, The binding site of MKM (blue) on the *E. coli* 70S ribosome, composed of 23S rRNA helices H13 (orange), H21 (pink) and H88 (purple), as well as the ribosomal protein bL28 (green). **b-f**, Superimposition of MKM binding site on the *E. coli* 70S ribosomes from (**a**) with (**b**) *M. smegmatis* 70S ribosome (red, PDB ID 5O61)^48^, (**c**) *S. aureus* 70S ribosome (cream, PDB ID 8P2G)^49^, (**d**) *P. aeruginosa* 70S ribosome (lime, PDB ID 6SPG)^103^, (**e**) *K. pneumoniae* bL28 (lime) and (**f**) *T. thermophilus* 70S ribosome (pale blue, PDB ID 6ND6)^104^ where *T. thermophilus* bL28 sterically clashes with MKM. Ribosomes were structural alignment based on the 23S rRNA.

**Extended Data Fig. 8:**
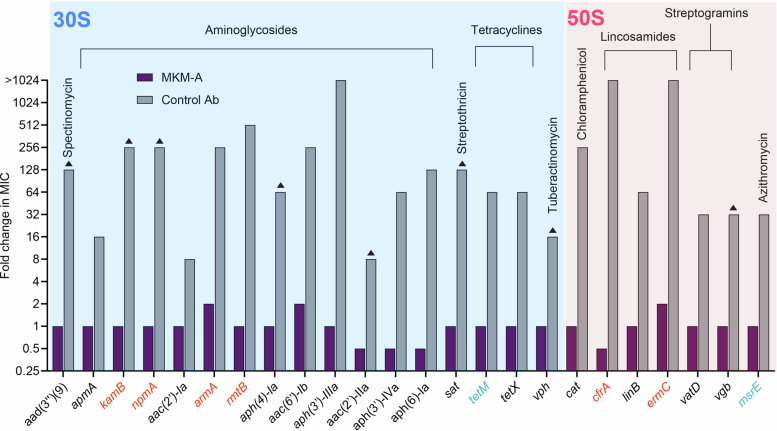
MKM activity is unaffected by common resistance mechanisms to ribosome-targeting antibiotics. The graph shows the MIC increase of MKM and control antibiotics upon overexpression of designated resistance determinants in *E. coli* BW25113 Δ*tolC*Δ*bamB*. For controls marked with black triangle, the actual MIC change exceeds the values shown, indicating bacterial growth at the highest antibiotic concentration tested. The gene name’s colour reflects the resistance mechanism: antibiotic modification – black, rRNA modification – red, ribosome protection – blue. Antibiotics targeting the small and large ribosomal subunits are shown on blue and pink background, respectively.

MKMs exhibit selective antimicrobial activity against Gram-negative Enterobacteriaceae (*E. coli* and *K. pneumoniae*) (Fig. 6a,b and Extended Data Table 1). They also readily inhibit the growth of mycobacteria (Extended Data Table 1). Conversely, MKMs are ineffective against many other Gram-negative and most Gram-positive bacteria, including gut microbes (Extended Data Table 1). Because the MKM-binding site is conserved in ribosomes across various bacterial species, including not only *K. pneumoniae* and *Mycobacterium* species^48^ (Extended Data Fig. 7) but also Gram-positive bacteria such as *Staphylococcus aureus*^49^ (Extended Data Fig. 7), the lack of antimicrobial activity of MKMs against such strains (Extended Data Table 1) is probably due to poor uptake (caused, for example, by the lack of the required transporters), rather than an inability to inhibit translation. Of note, owing to the unique site of action in the ribosome, MKM is insensitive to resistance mechanisms protecting bacterial pathogens from other translation-inhibiting antibiotics (Extended Data Fig. 8).

No haemolytic activity or mammalian cytotoxicity against HEK293 and HepG2 cell lines was detected with MKMs at a concentration of up to 256 µg ml^−1^ (Extended Data Table 1). An ex vivo infection model using human blood inoculated with 10^5^ colony-forming units per ml of *K. pneumoniae* C1559 showed approximately 1,000-fold reduction in bacterial load after 6 h of exposure to 5× MIC of MKM (Fig. 6c), indicating the potential effectiveness of MKM in treating bloodstream infections. Initial attempts to evaluate MKM in a murine infection model did not demonstrate efficacy, potentially owing to pharmacokinetic issues (Supplementary Fig. 35). To further assess in vivo efficacy, we turned to a *Caenorhabditis elegans* infection model, which offers a convenient and physiologically relevant system for antimicrobial testing. *C. elegans* was infected with *K. pneumoniae* reference strain ATCC 33495 or with the C1559 multidrug-resistant clinical isolate. Infected nematodes were treated with MKM (64 µg ml^−1^), polymyxin B (25 µg ml^−1^) or DMSO, and survival was tracked over 6 days (Fig. 6d). MKM markedly improved survival in both infection models, with 55–60% of nematodes remaining alive by day 6, compared with 10% (ATCC 33495) and 30% (C1559) in the DMSO-treated groups. Polymyxin B provided a similar survival benefit.

## Discussion

Microbial antibiotic producers may simultaneously produce and secrete several different antimicrobial compounds. In such a scenario, the antibacterial activity of the more moderately expressed inhibitors may be obscured by the most dominant antibiotic. Indeed, *S. rimosus* has been known as the producer of oxytetracycline for over 75 years and, more recently, as the producer of the Type V glycopeptide antibiotic rimomycin^50^. Only careful fractionation allowed the detection of MKM, a previously unknown antibiotic with an idiosyncratic mechanism of action. It is likely that many other unknown antibiotics may still be masked by the well-studied antibacterials secreted by the well-exploited antibiotic-producing *Streptomyces* strains.

MKMs represent a new antibiotic chemical scaffold. The NRPS modules of ManAB predict the production of an Arg-enriched decapeptide corresponding to MKM-E, which we detected in extracts of *S. rimosus* and under MKM BGC heterologous expression in *S. coelicolor*. We further examined the adenylation domains of modules 1, 2, 3, 7 and 8, responsible for Arg activation. Notably, module 3 carries two mutations in its Stachelhaus motif^51^ (a set of residues that confer substrate specificity to adenylation domains) that correlate with the variable incorporation of Orn or Arg at this position across MKM variants (Supplementary Fig. 32), suggesting substrate promiscuity. Although rare, such flexibility in adenylation domains has been observed previously, such as in the PheATE module of gramicidin S synthetase, which activates multiple aromatic amino acids^52^.

MKM is the first antibiotic, to our knowledge, that targets the E-site of the large subunit of the bacterial ribosome. The importance of the E-site for translation has long been debated^53,54^, and the availability of the large subunit E-site inhibitor MKM will likely serve as a useful tool for investigating the contribution of this functional ribosomal site to the general mechanism of protein synthesis. The binding mode of MKM (MKM-A) determined here is also compatible with other identified MKM congeners, including MKM-B and MKM-E (Supplementary Fig. 33), suggesting that these forms are likely to have the same mechanisms of action and inhibit translocation in a context-specific manner as elucidated here for MKM-A.

Although several E-site inhibitors of eukaryotic or archaeal protein synthesis are known, none of them interfere with bacterial translation, owing to the structural heterogeneity of the E-site across the evolutionary kingdoms (Extended Data Fig. 5). In turn, the difference in the ribosomal E-site architecture between bacteria and eukaryotes accounts for the therapeutic selectivity of MKM, which is a very weak inhibitor of mammalian translation and does not exhibit toxicity to cultured mammalian cells. Furthermore, because MKM is the first known bacterial large ribosomal subunit E-site inhibitor, none of the ribosome-based resistance mechanisms found in clinical isolates protect sensitive cells from MKM action (Extended Data Fig. 8).

The known E-site inhibitors of eukaryotic translation exhibit contrasting modes of action. Whereas cycloheximide arrests the elongating ribosome, structurally related lactimidomycin captures the ribosomes at start codons but has little effect on translation elongation or termination^23,29,55^. Superficially, the mode of action of MKM resembles that of cycloheximide, since MKM also stalls the elongating ribosome. However, the mechanism of translation inhibition by cycloheximide and MKM are likely to be principally different. Because of the small size of cycloheximide, it was proposed to co-accommodate in the E-site of the eukaryotic ribosome together with the displaced CCA end of the E-site tRNA^23^. This is unlikely to happen with the much bulkier MKM, which nearly completely occludes the E-site cavity of the bacterial 50S subunit, thereby blocking the entrance of the CCA end of deacylated tRNA and preventing formation of the P/E hybrid state and translocation (Figs. 3i and 4a). However, we cannot rule out that some P/E hybrid states can form in the presence of MKM—for example, with tRNA^Thr^, explaining why it may be less susceptible to MKM action.

The severity of translation inhibition by most elongation inhibitors often depends on the sequence of the mRNA or the nascent polypeptide being translated^36^. MKM does not directly interact with mRNA or with the growing protein chain. Furthermore, in addition to the ribosome, potential contacts with other players of the translation process are likely to be restricted to the universally conserved CCA ends of E-site tRNAs. Therefore, it was unexpected to find that the bacterial E-site inhibitor still acts in a context-specific manner, more efficiently inhibiting ribosomes translating Pro and Leu codons and exhibiting less effect on those translating Thr codons. Although we do not fully understand the structural reasons for the context specificity of MKM action, it is likely to be related to the kinetics of translation elongation or the duration of the E-site tRNA retention. Faster E-site tRNA dissociation, slow accommodation of the A-site tRNA or slow peptide bond formation would provide a better opportunity for the MKM binding in the vacant E-site. Conversely, longer occupancy of the E-site by the deacylated tRNA or preferential antibiotic displacement by certain tRNAs during formation of the ribosome hybrid state (Fig. 4a) would make MKM-induced ribosome stalling less likely. Reduced obstruction of ribosome transit through such codons prior to the drug-induced ribosome arrest could possibly account for the underrepresentation of threonine codons at and prior to the sites of the drug-induced translation arrest (Fig. 4e). Further investigations can be carried out to identify structural properties of tRNA^Thr^ that could account for such an effect. Moreover, exploring the mechanistic principles of the context specificity of MKM may illuminate unknown aspects of the translation process.

Foreseeing possible mechanisms of resistance necessitates staying ahead in the arms race against bacterial pathogens. Therefore, understanding how bacteria can avoid inhibition by MKM provides us a healthy head start. Our results show that mutations in the peptide transporters provide a low level of resistance. Altering the chemical properties of MKM, manipulating the overall charge, and making its entrance into the cell independent of the peptide transporters could potentially overcome the uptake-based resistance mechanism and broaden the antimicrobial spectrum. The MKM producer avoids self-toxicity by expressing the ManE rRNA methyltransferase that 2′-O-methylates the 23S rRNA C2395 residue located in the drug binding site (Fig. 5). It is conceivable that if MKM is developed into a drug, the *manE* gene may eventually be acquired by bacterial pathogens. However, our high-resolution structure of the ribosome-MKM complex provides a path for evading the ManE-based resistance by modifying residues of MKM, such as His9, that clash with the C2395 2′-*O*-methyl (Fig. 5e).

MKM-A showed acceptable acute tolerability in mice at doses up to 220 mg kg^−1^ per day, but no efficacy was observed in initial mouse infection models, prompting a comprehensive pharmacokinetic evaluation. In vitro pharmacokinetics revealed excellent stability in mouse and human plasma (Supplementary Fig. 35a,b). However, MKM-A exhibited suboptimal peak plasma concentration (*C*_max_ = 9.13 µg ml^−1^) and rapid systemic elimination, with a terminal half-life of about 36 min after a single subcutaneous dose of 50 mg kg^−1^ (Supplementary Fig. 35c). These results suggest that insufficient plasma exposure, rather than inherent pharmacological inactivity, accounts for the lack of efficacy observed in our experiments. Follow-up studies will focus on improving the pharmacological properties of MKM. Notably, the polycationic nature of MKM may pose liabilities commonly associated with this class, including potential nephrotoxicity and pharmacokinetic limitations, and these will need to be carefully addressed. However, the scaffold is amenable to chemical modification, providing a clear opportunity to modulate these properties while maintaining antimicrobial activity. Efforts to create and assess analogues are currently underway.

Offering a novel antibiotic scaffold with a new site of action on the bacterial ribosome and activity against challenging Gram-negative pathogens, MKM shows promise for further development. By revisiting ‘old’ antibiotic producers, this study shows that there remains a wealth of antibiotic chemical diversity to be mined in microbial genomes.

## Methods

### Bacterial strains and plasmids

Bacterial strains and plasmids used in this study are listed in Supplementary Table 7.

### Screening of WAC library for antimicrobial activity

The pre-fractionation library from the WAC^6^ was screened against hyperpermeable efflux-deficient *E. coli* BW25113 Δ*tolC*Δ*bamB* in 384-well microtitre plates (Corning 3701). Each well contained 49 μl of inoculated Mueller–Hinton broth (MHB) medium (cation-adjusted MHB (BD Difco)) and 1 μl of crude methanolic extract, fractions, or conditioned medium. A Biomek FXP Integrated Liquid Handler was used to dispense the fractions, extracts, and inoculated media into the plates. Plates were incubated at 37 °C for 20 h in a static incubator. Cell growth was measured by OD at 600 nm using EnVision, SpectraMax, or Biotek Neo microtitre plate readers.

### Purification of MKMs

*S. rimosus* WAC 7405 was routinely cultured in tryptic soy broth (TSB) medium (Difco) in 225 ml flasks for 16 h, before inoculating at 1% (v/v) into ASM medium^58^ in 2.8 l flasks for 4 days. Cultures were maintained at 30 °C, shaking at 220 rpm.

During initial discovery, the active compound, as defined by routine testing against *E. coli* BW25113 Δ*tolC*Δ*bamB*, was isolated from conditioned medium mixed with 5% (v/v) Diaion HP-20 resin, mixed for 2.5 h. HP-20 resin was filtered using a milk filter and extracted with 300 ml of methanol for 2 h. The extract was collected and dried by rotary evaporation. After reconstitution in 10 ml water, compounds were separated by Sephadex LH-20 column (300 ml bed volume) and eluted with 50% methanol to yield 7× 50 ml fractions. Active fractions were analysed by the liquid chromatography–tandem mass spectrometry (LC–MS/MS) method; for this analysis, mass range was set to 150–2,500 *m*/*z* at a scan rate of 1 spectrum s^−1^. Three collision energies of 10, 30, 60 eV were selected with a medium isolation width of 4 atomic mass units. The liquid chromatography was performed using a gradient of H_2_O (0.1% formic acid v/v) and acetonitrile on an Eclipse SDB-C8 column (2.1 mm ID × 100 mm, 3.5 µm). The flow rate was 0.4 ml min^−1^, and the gradient started with 10% B for 2 min, followed by a linear gradient to 100% B over 15 min. After this, the fractions were assessed by GNPS^8^ to identify known compounds. Masses consistent with oxytetracycline were identified in fractions 3, 4 and 5. Fractions 1 and 2 were loaded as a liquid load on reverse-phase Combi Flash column (RediSep Rf C18 High performance Gold-50g, Teledyne) and eluted with a linear gradient of H_2_O (0.07% trifluoroacetic acid, solvent A) and acetonitrile (0.07% trifluoroacetic acid, solvent B). Active fractions were purified further by preparative reversed-phase high-pressure liquid chromatography (RP-HPLC- Agilent technologies) using C8 column (Eclipse XDB C8 Semi Prep 9.4 × 250 mm, 5 μm, Agilent Technologies) with a gradient of 5% to 20% of solvent B in 20 min. MKM-A and MKM-B were eluted at retention times of 17.5 and 18.5 min, respectively.

Later purifications were optimized as follows. Seed cultures in TSB were cultured for 2 days and inoculated into ASM at 10% (v/v). After HP-20 extraction, the sample was processed using SP-Sepharose cation-exchange chromatography. The column was pre-equilibrated with a 10 mM ammonium acetate buffer (buffer A; pH 5.0–5.2). The sample pH was adjusted to the same range. The column was washed with buffer A and 1 M NaCl in buffer A at pH 5.0. MKMs were eluted in 1 M NaCl in buffer A at pH 8.5–9.5. Fractions were neutralized using 0.6 N HCl during the elution. Following combiflash separation, as described above, analogues of MKM were resolved on a Chromatik Sunniest C28 RP-Aqua Semi Prep column for HPLC (10 × 250 mm, 5 µm) Purity of the compounds (>95%) was confirmed with a C28 analytical column (Sunniest RP-Aqua C28 4.6 × 100 mm, 5 µm). MKM-A (most abundant product), MKM-B, and MKM-E were purified as single peaks. Yield variations are shown in Supplementary Fig. 25.

### Structural characterization of MKMs

High-resolution electrospray ionization mass spectra were acquired using an Agilent 1290 UPLC separation module and a qTOF 6550 mass detector in positive ion mode. For general liquid chromatography separation an Agilent Eclipse XDB C8 column (2.1 × 150 mm; 3.5 µm) and the following method were used: from 0 to 1 min 75% A (0.1 v/v formic acid in water), from 1 to 7 min a linear gradient to 100% B (0.1 v/v formic acid in acetonitrile) at a flow rate of 0.4 ml min^−1^. The NMR spectra were recorded on an AVIII 700 MHz NMR spectrometer, equipped with a cryoprobe. The compounds used in this study were dissolved in deuterated water as a solvent (Cambridge Isotope Laboratories) to a concentration of approximately 5.0 mg ml^−1^. Chemical shifts are reported in ppm relative to tetramethylsilane using the residual solvent signal at ppm. Chemical shifts values are expressed in ppm (*δ*), coupling constants (*J*, Hz) and peak patterns are reported as broad singlet (bs), singlet (s), doublet (d), triplet (t), quartet (q), pentet (p) and multiplet (m). MKM-A (1 mg) was treated with 6 N HCl (1 ml) in a sealed tube at 110 °C for 24 h. The reaction mixture was then extracted with ethyl acetate and the aqueous solution was dried under nitrogen. To the aqueous residue (10 µl) was added 1 M NaHCO_3_ (10 µl), followed by Marfey’s reagent (1-fluoro-2,4-dinitrophenyl-5-d-alanine amide) (50 µl, 1% solution in acetone). The reaction was then carried out for 1 h at 40 °C and stopped by the addition of 1 N HCl (10 µl) and methanol (420 µl)^59,60^.

On the basis of the predicted structure by NMR and HRMS/MS data, the following reference amino acids were selected for modification with Marfey’s reagent: l-Phe, dl-Phe, l-His, dl-His, l-Orn, dl-Orn, l-Thr, dl-Thr, l-Asn, dl-Asn, l-Arg and dl-Arg. In brief, to 10 µl of 10 mg ml^−1^ solution of the corresponding amino acid was added 10 µl of 1 M NaHCO_3_, followed by Marfey’s reagent (50 µl, 1% (w/v) solution in acetone). The reaction was carried out as described above. The standards and sample hydrolysate were analysed on a LC–MS system (qTOF 6550 coupled to UPLC 1290, Agilent Technologies) with an optimized method to best resolve between individual modified amino acids: 0–0.5 min, 10% solvent B, 0.5–20 min, linear gradient to 30% B, 20–40 min linear gradient to 65% B. Solvent A was 0.1% formic acid in water and solvent B was acetonitrile (100%) at a flow of 0.2 ml min^−1^ using an Agilent Eclipse XDB C8 column (2.1×150 mm; 3.5 µm). The absolute configuration of the chiral amino acids was assigned as d-Arg, d-Orn, d-Phe, d-Asn, l-Thr and l-His. Mass spectrometry analyses of MKM variants are shown in Supplementary Fig. 24 and Supplementary Table 4.

### Whole-genome sequencing and BGC analysis

Genomic DNA extraction and Illumina sequencing were performed as described^61^. The NEB Next Ultra V1 kit was used with 500 ng of sonicated DNA from WAC 7405 and AMPure XP beads were used for size selection. Library preparation and sequencing were performed at the McMaster Genomics Facility. Skewer (v0.2.2)^62^ and FLASH (v1.2.11)^63^ were used for trimming and merging reads, and SPAdes (v3.11.1)^64^ for de novo assembly. Illumina assemblies are available from BioProject ID PRJNA1273197. For nanopore sequencing, 400 ng of high molecular weight genomic DNA was prepared with Oxford Nanopore’s Rapid Barcoding Kit and sequenced on a MinION R9.4.1 flow cell. Reads were assembled with Unicycler (v0.4.9b)^65^ and SPAdes (v3.13.0)^64^. MKM BGCs were identified by analysing the genome sequence using antiSMASH (v6.0.0)^66^ and antiSMASH (v8.0)^67^. The gene annotations of the MKM BGC are shown in Supplementary Table 5.

### Heterologous expression of MKM

The MKM BGC was captured using transformation-associated recombination cloning^10,68^. DNA sequences that target MKM BGC were designed by identifying boundary sequences of the BGCs that included suitable yeast transcription start sites, as outlined in Supplementary Table 8. pCGW^69^ was linearized using NdeI and XhoI restriction enzymes, and the synthesized gBlocks were introduced through Gibson assembly to generate vector MKM-Gbk. The capture vector was linearized with PmeI. Genomic DNA (gDNA) from WAC 7405 was isolated using the salting-out method and treated with RNase A to remove RNA. The purified gDNA was then digested with BstZ17I/XhoI and BstZ17I/XmaJI, sites flanking the MKM BGC. Digested gDNA was further purified via sodium acetate precipitation. Linearized pCGW-gBlocks capture plasmid (~500 ng) and digested gDNA (~2 µg) were co-transformed into *Saccharomyces cerevisiae* VL6-48N spheroplasts. Captured clones were selected on sorbitol containing SD-Trp (synthetic defined minus Trp) medium (91 g sorbitol (1 M), 10 g glucose (2%) and 10 g agar (2%)) containing 0.1% 5-fluoroorotic acid as described^70^. Positive clones were obtained after 3–5 days at 30 °C. Yeast transformants were cultured in SD-Trp medium for 24 h, and plasmid DNA was extracted using the alkaline lysis method for PCR screening. Positive transformants were re-transformed into *E. coli* EPI300 cells by electroporation and confirmed via restriction digestion mapping.

Plasmid pCGW7405 (Supplementary Fig. 34) was reintroduced into *E. coli* ET12567 cells via electroporation and mobilized into the host strain *S*. *coelicolor* M1154 through *E. coli*-*Streptomyces* tri-parental mating, using *E. coli* ET12567/pR9406 as the helper strain^71^ as described previously^72^.

### Overexpression of ManE rRNA methyltransferase

The ManE gene from the gDNA of the WAC 7405 strain was amplified using primers, METH-TRANS-FP and RP and digested with XhoI and NdeI and cloned into the corresponding sites in pGDP3, a low copy plasmid with a P_lac_ promoter^73^. The corresponding sequence validated plasmid was transformed into *E. coli* BW25113 and BW25113 Δ*tolC*Δ*bamB* strains to assess the impact on MKM susceptibility.

### MIC determination

MIC against Gram-negative bacteria was assessed using the broth microdilution method in cation-adjusted MHB (MHBII, BD Difco), RPMI-1640 (Sigma Aldrich) and MOPS minimal medium with 0.4% glucose (M2106 Teknova), and MIC against *S. aureus* and *Bacillus subtilis* was determined in MHBII following standard procedures, unless specified otherwise^74^. Mycobacterial MICs were performed in Middlebrook 7H9 medium supplemented with 10% OADC Enrichment (oleic acid, bovine albumin, dextrose and catalase) (BD Difco) and 0.05% Tween-80. Growth (measured as colony-forming units per ml (CFU ml^−1^) for all mycobacterial strains was confirmed to be within the range of 1 × 10^5^ to 5 × 10^5^ cells per ml by growth on 7H10 + OADC agar. Anaerobic microbiota strains were cultured in BHI supplemented with l-cysteine (0.5 g l^−1^), haemin (10 mg l^−1^) and vitamin K (1 mg l^−1^) in anaerobic chambers (37 °C, 5% H_2_, 10% CO_2_, 85% N_2_).

### Testing cytotoxicity of MKM

Mammalian culture was performed in Dulbecco Modified Eagle Medium (DMEM) supplemented with 10% fetal bovine serum (FBS), 2 mM l-glutamine, 100 units ml^−1^ penicillin and 100 µg ml^−1^ streptomycin. HEK293 cells (ATCC CRL-1573; generation 6) were seeded at 7,500 cells per well and HepG2 cells (ATCC HB8065; generation 18) were seeded at 4,000 cells per well in 384-well tissue culture-treated white plates in 50 µl DMEM and incubated for 18 h at 37 °C in the presence of 5% CO2. After 18 h of incubation, 500 nl of the compound and DMSO (1% final concentration) were added to the cells using a Labcyte Echo acoustic dispenser (Beckman Coulter). Cells were further incubated for 48 h and after that cell viability was assessed using Promega Cell Titer Glo 2.0 reagent (Fisher Scientific). Before incubating the plates at room temperature for 10 min, 50 µl of Cell Titer Glo was added directly to the medium. The plates were then shaken for 2 min. The Neo2 plate reader (Biotek) was used to read the luminescence through luminescent fibre. Cells that were either not treated or solely treated with DMSO were used as a control.

### Haemolysis assay

Human blood was obtained from BioIVT (USA). Red blood cells (RBCs) were separated from blood by centrifugation at 500*g* for 5 min. Plasma was removed and the RBCs were washed twice with 150 mM NaCl, using a volume equivalent to the removed plasma. After washing, phosphate-buffered saline (PBS) with a pH of 7.4, in a volume equal to the removed plasma, was added to create the RBC suspension. Using a Labcyte Echo acoustic dispenser (Beckman Coulter), 1 µl of the compound was added to the wells of a 96-well V-bottom plate. The final concentration of DMSO was kept at 1% (v/v), with a DMSO-only negative control included in each replicate. For the positive control, 10 µl of Triton X-100 was added, starting at a concentration of 20% and serially diluted twofold to 0.02%. RBCs were diluted 1:50 in PBS (pH 7.4), and 99 µl of this diluted solution was added to each well. The plates were incubated at 37 °C for 1 h, then centrifuged at 500*g* for 5 min to pellet the intact RBCs. Sixty-five microlitres of the supernatant was transferred to a clear, flat-bottom 96-well plate, and the absorbance was measured at 540 nm.

### Time-dependent killing assay

*E. coli* BW25113 and *K. pneumoniae* C1559 were grown overnight in 5 ml of cation-adjusted MHB. The cultures were then inoculated into fresh medium to achieve an optical density at 600 nm (OD_600_) of 0.1, corresponding to a concentration of 10^8^ CFU ml^−1^. The culture was diluted 100 times, and 1 ml of the cell suspension was treated with either 5× MIC of MKM-A (160 μg ml^−1^ for *E. coli* and 80 μg ml^−1^ for *K. pneumoniae*) or tetracycline (5 μg ml^−1^ for *E. coli* and 40 μg ml^−1^ for *K. pneumoniae*) in a 1 ml culture, incubated at 37 °C with agitation at 250 rpm. Samples were taken at 0 h, 3 h, 6 h and 24 h post-incubation, plated on Mueller–Hinton agar plates, and incubated for 20–24 h at 37 °C. Colony counts were used to determine the log reduction in CFU ml^−1^, comparing treated strains to untreated control samples.

### Propidium iodide uptake assay

*E. coli* BW25113 cells were grown to mid-exponential phase in MOPS minimal medium, and the OD_600_ was adjusted to 0.1–0.2. Cells were mixed with propidium iodide at a final concentration of 4 μM. A 190 μl aliquot of the cell suspension was added to the wells of a 96-well black-wall plate, followed by the addition of 10 μl of compounds at different concentrations up to 10× MIC (80 µg ml^−1^). Colistin (5 µg ml^−1^) was included as a positive control. Fluorescence was measured at an excitation/emission wavelength of 535/617 nm for 30 min at room temperature using a Synergy Microplate Reader (Biotek).

### Assessing outer membrane permeability by NPN assay

An overnight culture of *E. coli* BW25113 was subcultured into fresh MOPS minimal medium and allowed to grow until reaching mid-exponential phase. The culture was then diluted to an OD _600_ of 0.1–0.2 in the same medium and supplemented with 10 µM *N*-phenyl-1-naphthylamine (NPN) dye (prepared from a 20 mM stock solution in acetone). A volume of 190 µl of this cell suspension was combined with 10 µl of test compounds at various concentrations in a black 96-well plate. Water served as the vehicle control, as MKM-A was dissolved in water (the vehicle is adjusted accordingly on the basis of the solubility of the compound). Colistin at 5 µg ml^−1^ was included as a positive control. Fluorescence was measured over a 30 min period at room temperature using a microplate reader, with readings taken every 0.5–1 min at an excitation/emission wavelength of 350/420 nm.

### Scanning electron microscopy

Approximately 10^8^ cells of exponentially growing *E. coli* BW25113 were exposed to MKM at concentrations of 32 μg ml^−1^ (5× MIC) and 80 μg ml^−1^ (10× MIC) in MOPS minimal medium for 1 h at 37 °C. After treatment, the cells were centrifuged at 5,000*g* for 5 min, then resuspended in a fixative solution (4% glutaraldehyde in PBS, pH 7.4) at 0.1× the original volume. The cells were fixed at room temperature for 1 h and stored overnight at 4 °C. The following day, 50 μl of the fixed cells were transferred onto coverslips coated with poly-l-lysine, dehydrated through a series of ethanol treatments, and dried using a critical point dryer. The samples were then examined with a scanning electron microscope (TESCAN VEGA-II LSU) equipped with an X-MAX 80 mm^2^ EDS detector, and images were captured using INCA software.

### Resistance studies

To develop resistance through sequential passaging, *E. coli* BW25113 cells were grown to an OD_600_ of 1–2 and then were diluted 200-fold in 1 ml of cation-adjusted MHB medium. The cells were incubated at 37 °C with shaking in the presence of varying concentrations of MKM-A (0.25×, 0.5× and 1× MIC) and passaged every 24 h for 15 days, with the treatment concentration increased daily as per growth to promote resistance development. The MIC of the resulting mutants was determined using broth microdilution. Genomic DNA from two resistant mutants (Ecmut1 and Ecmut2) was sequenced using Illumina and analysed with Breseq v0.37.1 to identify mutations relative to the parental strain^75^.

To select spontaneous mutants, approximately ~10^9^ CFU of *E. coli* BW25113 and *K. pneumoniae* C1559 were plated on cation-adjusted Mueller–Hinton agar, and ~10^9^ CFU of *E. coli* BW25113 were also plated on MOPS minimal agar containing 8× MIC of MKM-A. The plates were incubated at 37 °C for 24–48 h. The resulting colonies were tested for MKM-A susceptibility. The frequency of resistance was determined by dividing the number of colonies obtained on the treated plates by numbers of colonies plated.

To select resistance mutations in rRNA genes, ~10^9^ CFU of *E. coli* SQ110Δ*tolC* pZ-*sbmA* cells^20,21^ overexpressing the transporter involved in MKM uptake were plated on LB agar supplemented with 32 µg ml^−1^ (4× MIC) of MKM-A, 100 µg ml^−1^ ampicillin, 50 µg ml^−1^ kanamycin, 50 µg ml^−1^ spectinomycin, and 50 uM IPTG. The plate was incubated overnight at 37 °C, the single colonies were randomly picked. The unique 23S rRNA gene (*rrlE*) of this strain was amplified by PCR from 11 clones using the primers 23S_rRNA_F and 23S_rRNA_R and Sanger sequenced.

### In vitro transcription–translation assay

The impact of MKM-A on in vitro protein synthesis was evaluated using the *E. coli* S30 extract transcription–translation system (Promega) following the manufacturer’s protocol. The pBESTluc plasmid DNA was utilized as the template to produce firefly luciferase. MKM analogues were tested across a concentration range of 0.05 to 100 µM. The reactions were incubated for 1 h at 37 °C. Luminescence was then measured in opaque 96-well plates using a Synergy Microplate Reader (Biotek). The IC_50_ values, indicating the concentration at which MKMs inhibited protein synthesis by 50%, were determined using GraphPad Prism 10 software.

### Toeprinting analysis

Toeprinting analysis was carried out in the *E. coli* in vitro transcription–translation system assembled from the purified components (PURExpress, NEB). Toeprinting was carried out using either ^32^P-radiolabelled or fluorescently labelled reverse transcription primers, following the procedures described previously^21,26,27^.

Reactions either contained no antibiotic or were supplemented with 50 μM retapamulin or varying concentrations of MKM. The inhibitors of aminoacyl-tRNA synthetases (mupirocin, Gly-AMS (MedChemExpress, HY-108940) or Arg-AMS (MedChemExpress, HY-112862)) were added to the final concentrations of 50 μM to stall translation at downstream catch codons (Ile, Gly or Arg, respectively). The *gltX* template was prepared by amplifying the *gltX* gene from *E. coli* BW25113 genomic DNA using the primers T7_gltX_F and gltX_NV1_R (Supplementary Table 8). The *yrbA*_wt template and its derivatives containing 3-Ser or 3-Thr codons were generated by four-primer PCRs using the primers T7_IR_AUG_F, yrbA_wt_F/yrbA_TTT_F/yrbA_SSS_F, yrbA_wt_R/yrbA_Ile_catch_R and posT-NV1_R (Supplementary Table 8).

Fluorescently labelled reactions were carried out on the *ermBL* toeprint mRNA template. The template was generated by PCR of two overlapping 77-nt- and 78-nt-long primers T7_ermBL_F and ermBL_UGA_R (Supplementary Table 8). Reactions were assembled in a 6 µl volume with 30 ng of the *ermBL* mRNA template and incubated for 15 min at 37 °C. Reverse transcription was carried out using AMV reverse transcriptase and primer NV*1-Alexa 647 (Supplementary Table 8) for 20 min at 37 °C. Reactions were terminated with 1 µl of 5 M NaOH, neutralized with 0.7 µl of 25% HCl, and nucleotide removal was performed with the QIAquick Nucleotide Removal Kit (Qiagen). The samples were dried under vacuum for 2 h at 60 °C for subsequent gel electrophoresis. The 6% acrylamide gels were scanned on a Typhoon scanner (GE Healthcare). The sequences of all toeprinting templates used in the study can be found in Supplementary Table 9.

### Preparation of complexes for structural analysis

MKM–ribosome complexes were generated by in vitro translation reactions in the PURExpress In vitro Protein Synthesis Kit (NEB) as described by the manufacturer. Complex formation reactions were carried out on *ermBL* toeprint mRNA template (Supplementary Table 9) in a 75 µl of reaction in presence of 50 µM MKM. The reaction was incubated for 15 min at 37 °C. The reaction volume was then split: 69 µl were used for complex generation and 6 µl were further analysed by toeprinting. Ribosome complexes were isolated by centrifugation in 900 µl of sucrose gradient buffer (containing 40% sucrose, 50 mM HEPES-KOH, pH 7.4, 100 mM potassium acetate, 25 mM magnesium acetate and 6 mM 2-mercaptoethanol) for 3 h at 4 °C with 80,000*g* in a Optima Max-XP Tabletop Ultracentrifuge with a TLA 120.2 rotor. The pelleted complex was resuspended in Hico buffer (50 mM HEPES-KOH, pH 7.4, 100 mM potassium acetate, 25 mM magnesium acetate) supplemented with 50 µM MKM, then incubated for 10 min at 37 °C, similarly to that described previously^26,27^.

### Preparation of cryo-EM grids

Cryo-EM grids were prepared by applying 3.5 µl of MKM–70S complexes onto freshly glow-discharged Quantifoil R3.5/1 grids (copper, 300 mesh, with an additional 3 nm carbon layer; C3-C19nCu30-01). The glow discharge was performed using a GloQube Plus system (Quorum Technologies) at 25 mA for 30 s, in a negatively charged atmosphere. Vitrification of the samples was carried out with a 1:2 ethane-to-propane mixture using a Vitrobot Mark IV (Thermo Scientific). The chamber was maintained at 100% relative humidity and 4 °C. Blotting was performed for 3.5 s at blot force 0, using Whatman 597 filter paper. After vitrification, the grids were loaded into autogrid cartridges and stored in liquid nitrogen until further use.

### Data acquisition

Data acquisition was conducted on a Titan Krios G3i transmission electron microscope (Thermo Fisher Scientific/FEI) operating at the Center for Structural Systems Biology (CSSB), Hamburg. The microscope was operated in fringe-free imaging (FFI) mode, equipped with a K3 direct electron detector and a BioQuantum energy filter with a 20 eV slit width. Prior to data collection, gain reference and GIF fine-centring were completed. Automated data acquisition was carried out using EPU software (v3.2.0.4775REL).

Movies were captured at a nominal magnification of 105,000×, corresponding to a calibrated pixel size of 0.832 Å (0.416 Å in super-resolution mode, binned 2× via EPU). The dataset was collected using defocus values ranging from −0.3 µm to −1.0 µm in 0.1 µm increments between holes. Each exposure lasted 1.95 s in nanoprobe mode, during which 35 frames were recorded at a dose rate of ~1.14 electrons per frame per Å^2^, resulting in a total accumulated dose of approximately 40 electrons per Å^2^ (~15 e^−^ px^−1^ s^−1^ over vacuum). A 70 µm C2 aperture and beam spot size 7 were used. Objective lens astigmatism was corrected to below 1 nm, and coma-free alignment was refined to under 50 nm using Sherpa’s AutoCTF module (v2.11.1). In total, 5,455 gain-corrected TIFF micrographs of the MKM–70S complex were acquired.

### Cryo-EM data processing

RELION (v5.0.0)^76,77^ was used for image processing, unless specified otherwise. For motion correction, RELION’s implementation of MotionCor2 with 7 × 5 patches^78^, and, for initial contrast transfer function (CTF) estimation, CTFFIND (v4.1.14)^79^, were used. After motion correction and CTF estimation, 691,679 particles were picked using crYOLO^80^ (Supplementary Fig. 26a), and the particle coordinates were then imported into RELION. 2D classification with 100 classes was performed and 483,608 ribosome-like particles were selected for further processing (Supplementary Fig. 26b,c). After 2D classification, all ribosome-like particles were selected, extracted with pixel size of 2.49 Å, and 60 Å low pass filtered 70S ribosome (PDB ID 7K00)^28^ was used as reference to perform 3D consensus refinement of these particles. With this 3D refined map, 3D classification was performed without angular sampling. All classes that contained 70S ribosomes at high resolution were used for further processing. Particles with homogenous 3D class distribution were re-extracted using smaller pixel size and subjected to 3D refinements. Subsequently, CTF refinements were performed to correct for anisotropic magnification, defocus and astigmatism, beam tilt, trefoil and higher order aberration followed by Bayesian polishing^81^. After several rounds of 3D classifications and focused classification on the tRNA binding pockets (Supplementary Fig. 26d–f), focus refinement on the 50S (70S, P-tRNA, A-tRNA, 261,301 particles) led to a final average resolution (gold-standard Fourier shell correlation (FSC) = 0.143) of 2.40 Å (Supplementary Fig. 26g). The 70S containing classes were combined (70S complex, P-tRNA, A-tRNA, 340,201 particles) and refined to a final average resolution (gold-standard FSC = 0.143) of 2.45 Å (Supplementary Fig. 26h). Local resolution was calculated with RELION (v5.0.0)^76,77^.

### Generation of molecular models

The molecular models were based on the *E. coli* 70S ribosome (PDB ID 7K00)^28^. Starting models with individual chains of ribosomal proteins and rRNA were rigid body fitted using ChimeraX^82^ and modelled using Coot (0.9.8.92)^83,84^ from the CCP4 software suite (v8.0)^85^. Model refinement was done using Servalcat^86^. For the antibiotic MKM, without available 3D structure, models were generated using ChemDraw (PerkinElmer Informatics) with structural restrains generated using aceDRG^87^. Manual adjustments using real space refinement function was done using Coot^83,84^. The final molecular models were validated using Phenix comprehensive cryo-EM validation tool in Phenix 1.20–4487 (ref. ^88^) (Extended Data Table 2).

### Figure preparation for cryo-EM data

Particle orientations and their distribution was determined and plotted using Relion (v5.0.0)^76,77^. The Molprobity server^89^ was used to calculate map versus model cross–correlation at FSC = 0.5 for all maps (Supplementary Fig. 27). UCSF ChimeraX (v1.8)^82^ was used to isolate densities, colour zone maps and visualize density images. Models were aligned using PyMol (v3.0) (Schrödinger). Figures were assembled using Inkscape v1.3.

### Translocation assay

In vitro translocation assay was carried out using the model mRNA MFK (Supplementary Table 9) as described^58^. The mRNA was prepared by in vitro transcription of a PCR product amplified using the primers MF_F1, MF_F2, and MF_R (Supplementary Table 8). A 4.5 μl reaction containing 1 μM *E. coli* ribosomes, 0.5 μM mRNA, 1 μM tRNA_i_^Met^, 0.5 μM radiolabelled NV1 primer (Supplementary Table 8), 2 U μl^−1^ RiboLock RNase Inhibitor (Thermo), and antibiotic tested (50 μM MKM or 250 μM negamycin) in pure system buffer (PSB; 9 mM Mg(CH_3_COO)_2_, 5 mM K_3_PO_4_, 95 mM potassium glutamate, 5 mM NH_4_Cl, 0.5 mM CaCl_2_, 1 mM spermidine, 8 mM putrescine, 1 mM dithiothreitol, pH 7.3)^90^ was incubated for 20 min at 37 °C. Then *N-*acetyl-Phe-*N*-tRNA^Phe^^ (ref. 91^), in which amino acid is attached to the A3′ hydroxyl of the tRNA via an amide bond, was added to the final concentration of 2 μM followed by 10 min incubation at 37 °C. After addition of the *E. coli* EF-G and GTP to the final concentrations of 0.2 μM and 533 μM, respectively and incubation for 5 min at 30 °C, 1 µl of the mixture of AMV reverse transcriptase (Roche) and dNTPs (2.1 U/µl AMV reverse transcriptase and 2 mM dNTPs in PSB) was added, and the reactions were incubated for another 5 min at 30 °C. The reaction was stopped by addition of 200 μl of the resuspension buffer (300 mM NaCH_3_COO, 5 mM EDTA, 0.5% SDS), DNA was then isolated by phenol-chloroform extraction, precipitation by addition of 3 volumes of ice-cold ethanol, incubating at −70 °C for 15 min, and centrifugation for 30 min (4 °C, 20,000*g*). The reaction products were resolved in 6% sequencing polyacrylamide gel and imaged on the Typhoon phosphorimager.

### Semiquantitative analysis of methylated 23S ribonucleoside abundance

To identify the posttranscriptional modification installed by ManE 50S ribosomal subunits were isolated from *E. coli* BW25113 transformed with either empty vector pGDP3^73^ or pGDP3-*manE*, constitutively expressing ManE methyltransferase under the control of the P_bla_ promoter. Overnight cultures of the two strains were diluted 1:50 in 75 ml of LB medium supplemented with 100 μg ml^−1^ ampicillin and incubated with shaking for 5 h (37 °C, 240 rpm). The cultures were chilled on ice for 10 min, and the cells were collected by centrifugation at 4,400*g* for 10 min at 4 °C. Cell pellets were washed once with 20 ml of wash buffer (50 mM HEPES pH 7.6, 10 mM MgCl_2_, 50 mM NH_4_Cl), frozen in liquid nitrogen, and stored at −80 °C.

For isolation of the ribosomes, 0.5 g of frozen cell paste for each strain was resuspended in 0.7 ml of lysis buffer (20 mM Tris/HCl pH 8.0, 10 mM MgCl_2_, 100 mM NH_4_Cl, 5 mM CaCl_2_, 0.4% Triton X-100, 0.1% NP-40, 1 mg ml^−1^ lysozyme, 100 U ml^−1^ DNAse I (Roche), 320 U ml^−1^ SUPERase·In RNase Inhibitor (Invitrogen)) and incubated on ice for 30 min. Cell suspension was transferred into 3× 2-ml tubes containing 400 mg of Lysing Matrix B beads (MP Biomedicals) each. Cells were lysed in FastPrep-24 bead beater (MP Biomedicals) (3 min, 6.5 beats s^−1^). The tubes were centrifuged 12 min at 20,000*g* (4 °C) and 600 μl of clarified lysates were layered on top of 1.7 ml of sucrose cushion (20% sucrose, 20 mM Tris/HCl pH 8.0, 10 mM MgCl_2_, 100 mM NH_4_Cl) in the tubes for the S110AT rotor of Sorvall MX 120 Plus Micro-Ultracentrifuge (Thermo). Ribosomes were pelleted by centrifugation at 422,000*g* for 1 h (4 °C). The ribosome pellets were rinsed with 300 µl of resuspension buffer (20 mM Tris/HCl pH 8.0, 1.5 mM MgCl_2_, 100 mM NH_4_Cl) and then resuspended in 300 µl of the same buffer. The samples were centrifuged at 20,000*g* for 10 min (4 °C) and 70 A_260_ units from each sample were loaded on top of two tubes (12 ml each) with 5–20% sucrose gradients prepared in the following buffer: 20 mM Tris/HCl pH 8.0, 1 mM MgCl_2_, 100 mM NH_4_Cl. Tubes were centrifuged for 2.5 h at 273,000*g* (39,000 rpm) in SW 41 Ti rotor (Beckman). The contents of the tubes were fractionated using piston gradient fractionator (Biocomp Instruments) and the fractions containing 30S ribosomal subunits were collected.

Total RNA was isolated from the fractions by hot phenol/chloroform extraction procedure as follows: acid-phenol:chloroform:isoamyl alcohol pH 4.5 (125:24:1; Ambion) prewarmed to 65 °C was added to sucrose gradient fractions in 1:1 ration (v/v) and the mixture was incubated for 5 min at 65 °C with shaking (1,400 rpm) followed by centrifugation at 15,000*g* for 2 min. The aqueous phase was transferred to a new tube and phenol extraction was repeated with 1 vol of room temperature acid-phenol: chloroform: isoamyl alcohol mixture. After that 0.9 vol of chloroform was mixed with aqueous phase followed by immediate centrifugation. The RNA from aqueous phase was then precipitated by addition of sodium acetate, pH 5.5, to the final concentration of 300 mM and 1.1 vol of ice-cold isopropanol followed by 30 min incubation at −80 °C. RNA was pelleted by centrifugation at 20,000*g* 30 min (4 °C) and supernatant was discarded. The RNA pellet was rinsed with 0.8 ml of ice-cold 80% ethanol and then resuspended in 60 µl of 10 mM Tris/HCl pH 7.0. The quality of the 23S rRNA was analysed by agarose gel electrophoresis and the presence of C2395 modification was confirmed by primer extension (see ‘Primer extension’).

Twenty-five micrograms of RNA from each sample were digested overnight by 1 U of Nuclease P1 (NEB) at 37 °C in 50 µl reactions containing 1× P1 Reaction Buffer (NEB) supplemented with 0.8 mM ZnSO_4_. In order to convert the resulting ribonucleotides to ribonucleosides, 0.25 U of Shrimp Alkaline Phosphatase (rSAP, NEB) and 5.5 µl of 10× rSAP Buffer (NEB) were added and the reactions were incubated at 37 °C for 3 h.

Samples containing ribonucleosides were further purified by extraction with 90% acetonitrile:water to remove insoluble material and concentrated by vacuum centrifugation. Samples were dissolve in 90% acetonitrile:water and analysed by high-resolution LC–MS on an Agilent 6546 LC-Q-TOF by hydrophilic interaction chromatography according to published methods^92^. Samples were separated on an Agilent Poroshell 120 HILIC-Z column (2.7 μm, 2.1 × 150 mm) at 0.1 ml min^−1^ in a 10 mM ammonium acetate (pH 5.2)–acetonitrile gradient starting with 10% of acetonitrile to 60% in 32 min, followed by 4 min isocratic and returning to 10% in 1 min with isocratic again at 6 min and further 6 min post-run. The flow rate was set to 0.1 ml min^−1^. Retention times of methylated cytidine and guanosine nucleoside standards were defined using compounds obtained from Cedarlane (Cm, 5mC, Gm, 1mG, 7mG) and TargetMol (m2G).

Integrated ion intensities were determined for hydrogen, sodium, and potassium adducts of expected nucleosides, methylated nucleosides, and nucleobases produced through in-source fragmentation. Mass error for all analysed nucleoside ions was less than 5 ppm. Signals for all ions were normalized by the median intensity and compared between control or methyltransferase containing *E. coli* cells to identify ions with 1.5-fold or greater change in intensity.

### Primer extension

Total RNA was extracted from the corresponding strains of *E. coli* using the RNeasy total RNA extraction kit (Qiagen). Primer extension analysis of rRNA modifications was performed using one microgram of total RNA essentially as described^93^. Primer L2507 (Supplementary Table 8) was used for the analysis of C2395 modification.

### Ribosome profiling

Ribo-seq experiments were performed as described^93^. In brief, the overnight culture of *E. coli* BL21Δ*tolC* was diluted 1:100 in 4 flasks (2 MKM-treated and 2 control samples) containing 100 ml of MOPS minimal medium (M2106 Teknova) each. The cultures were grown at 37 °C until reaching the OD_600_ of ~ 0.55. For MKM-treated samples, MKM-A dissolved in DMSO was added to the cultures to a final concentration of 50 µg ml^−1^ (25× MIC), and incubation continued for 2 min. Equivalent amount of DMSO was added to control samples for 2 min. Cells were collected by rapid filtration and flash frozen in liquid nitrogen. Cell pellets were resuspended in 300 µl of cold lysis buffer (20 mM Tris, pH 8.0, 10 mM MgCl_2_, 100 mM NH_4_CL, 5 mM CaCl_2_, 0.4% Triton X-100, and 0.1% NP-40) supplemented with 3 mM GMPPNP, 30 U RNase-free DNase I (Roche) and 96 U Superase•In RNase inhibitor (Invitrogen) and lysed by bead-beating with 300 mg of zirconium beads in the FastPrep-24 bead-beater (MP Biomedicals) for 1 min at 6.5 beats s^−1^. Cell lysates were clarified by centrifugation at 20,000*g* for 10 min at 4 °C. 22 A_260_ units of the clarified lysates were treated with 880 U of *S. aureus* Micrococcal Nuclease (MNase, Roche) for 60 min at 25 °C. The MNase reaction was quenched by addition of EGTA to final concentration of 6 mM. The lysates were layered over 2 ml of sucrose cushion (20% sucrose, 20 mM Tris/HCl pH 8.0, 10 mM MgCl_2_, 100 mM NH_4_Cl) in 4 ml tubes for S110AT rotor of Sorvall MX 120 Plus Micro-Ultracentrifuge (Thermo Fisher). Ribosomes were pelleted by centrifugation for 1 h at 422,000*g* (100,000 rpm). The pellets were resuspended in 500 µl of resuspension buffer (20 mM Tris/HCl pH 8.0, 10 mM MgCl_2_, 100 mM NH_4_Cl, 1% SDS) and frozen in liquid nitrogen. Subsequent isolation of ribosomal footprints and library preparation were performed as described^94^.

A script was used to demultiplex the samples, remove the linker barcode and then remove 5 nt from the 3′ end and 2 nt from the 5′ end, which were added as part of the library design^94^. Bowtie2 (v2.2.9)^95^ within the Galaxy pipeline^96^ first aligned the trimmed reads to the non-coding RNA sequences. The remaining unmapped reads were aligned to the reference genome of the *E. coli* strain BL21 (GenBank ID CP053601.1). The 24 nt- to 46 nt-long reads were used in the subsequent analyses. The first position of the P-site codon was assigned 15 nt from the 3′ end of the read^37^.

The metagene analyses at the annotated start and stop regions followed the described protocol^97^. Included in the analysis were the open reading frames (ORFs) that were: (1) separated by at least 50 nt; (2) with the length of 300 nt or more; (3) with at least 20% of the positions had assigned reads values above zero; and (4) with average number of reads per million mapped reads (RPM) per nt greater than 0.005. For the metagene plots, ribosome footprint density was normalized to the average coverage of the ORF, including 50 flanking nucleotides. The mean of the normalized values was computed and plotted for the ORF segments around the start and stop codons.

To analyse sequence specificity of MKM-induced ribosome stalling we first selected the codons in the bodies of the genes (excluding the first ten and last three codons of the genes), for which the ribosome occupancy was at least five times higher in the MKM-treated sample compared to the control (data from duplicates were merged for this analysis). For each site, the corresponding sequence of the amino acids was determined, and the over- or underrepresentation of amino acids for each position around the stall was analysed using the online pLogo tool^57^ (https://plogo.uconn.edu/) with selected (*n* = 5,248) and total (*n* = 194,045) samples of stalling sequences.

To evaluate the dependence of MKM-induced stalling efficiency on the identity of the codons in the ribosomes’ A, P and E-sites, we calculated the MKM stall score on a codon-by-codon basis throughout the genome and determined average stall scores for each of 61 sense codons (Extended Data Fig. 4b). For each codon MKM stall score was calculated as:

$${\rm{MKM}}\,{\rm{stall}}\,{\rm{score}}=\log 2\frac{({\rm{normalized\; codon\; RPM\; in\; the\; MKM\; sample}})}{({\rm{normalized\; codon\; RPM\; in\; the\; control\; sample}})}$$

Each codon RPM value was normalized to the total gene RPM. Only the codons having more than 5 aligned reads in both the MKM-treated and control samples were taken into the analysis. Genes showing fewer than 100 aligned reads were excluded from the analysis. Data from the duplicates were merged for this analysis.

### Ex vivo efficacy

Human blood was procured from BioIVT. *K. pneumoniae* C1559 cells were cultivated in cation-adjusted Mueller–Hinton medium until the OD_600_ reached 1.0. Then, 10 µl of these cells were inoculated into 990 µl of blood containing either 5× MIC of MKM-A or an equal amount of sterile water, followed by incubation at 37 ºC. Samples were obtained at 0 h, 3 h, and 6 h, and CFU values were determined by serial dilution and plating on Mueller–Hinton agar plates.

### *C. elegans–K. pneumoniae* in vivo antibiotic activity assay

A *C. elegans* double mutant strain, AU37 *(glp-4(bn2);sek-1(km4))*, was used for this assay due to its enhanced pathogen infection sensitivity and temperature-sensitive sterility. The infection protocol was carried out as previously described^98^, with slight modifications to accommodate our experimental conditions. Standard *C. elegans* media and protocols were used for the maintenance and growth of worms^99^. In summary, eggs were collected from gravid adult worms by bleaching and incubated on solid agar plates at 25 °C for 48 h, until early adulthood. Worms were thoroughly washed with M9 buffer and transferred onto LB agar plates containing lawns of infective *K. pneumoniae* C1559 and ATCC 33495 and were incubated for another 24 h. Worms were then washed from plates and resuspended in M9 to an approximate 2 worm per microlitre concentration. A survival assay was conducted in a 96-well plate with experimental wells containing 15 µl of worms, 80 µl S-basal (5.85 g NaCl, 1 g K_2_HPO_4_, 6 g KH_2_PO_4_, 1 ml cholesterol (5 mg ml^−1^ in ethanol), H_2_O to 1 l. Sterilize by autoclaving), and 5 µl of test compound. Plates were sealed with a porous film and incubated at 25 °C for a total of 7 days. The number of dead worms were counted every 24 h to generate a Kaplan–Meier survival curve.

### Identification of ManE-containing BGCs and phylogenetic tree construction

The protein sequence of ManE was used as a query in NCBI BLASTp. The results were manually curated to obtain non-redundant protein sequences. To analyse the corresponding genomes that showed the presence of *manE* homologues, antiSMASH (v.7.0) was used. The BGCs from each strain were extracted and compared using Clinker^100^. The manE-like methyl transferases from these BGCs were extracted and aligned with the in-built MUSCLE algorithm in MEGA11 (ref. ^101^) using default settings. This alignment was used to build a maximum-likelihood phylogenetic tree with MEGA11, utilizing the WAG substitution model, a bootstrap value of 100, and default parameters. An unrelated RlmE methyltransferase from *E. coli* K12 was used as an outgroup for tree rooting.

### In vitro plasma stability assay

Human and mouse blood (10 ml each) were collected into EDTA-coated tubes and centrifuged at 2,000*g* for 15 min at 4 °C to obtain plasma. Plasma samples were aliquoted and stored at −20 °C until use. For each assay, 500 µl of plasma was mixed with 500 µl of PBS (pH 7.4). The resulting 1 ml mixture was divided equally into two tubes (500 µl each). MKM-A was added from a 5 mM stock solution to a final concentration of 10 µM. Samples were incubated at 37 °C with shaking, and aliquots (50 µl) were collected at 0, 15, 30, 60 and 120 min. Each aliquot was immediately mixed with 50 µl of ice-cold acetonitrile, followed by centrifugation at 17,000 rpm for 5 min. Supernatant (10 µl) was removed, and 5 µl was injected onto an Agilent Eclipse Plus C18 column (1.8 µm, 2.1 × 100 mm) for LC–MS analysis with acetonitrile and water gradient in presence of 0.1% formic acid. Extracted ion chromatograms corresponding to the (M + 2H)^2+^ species of MKM-A were integrated, and peak areas were normalized to the PBS control containing 10 µM MKM-A, which was set to 100% for calculation of the percentage of compound remaining. Benfluorex (1 µM) served as the positive control, and its (M + H)^+^ ion (*m*/*z* 352.15) was quantified using the same workflow. Data analysis and curve fitting were performed in GraphPad Prism. All experiments were conducted in duplicate with two independent biological replicates.

### Pharmacokinetic studies in mice

All mouse experiments were performed in the Central Animal Facility at McMaster University under Animal Use Protocol 24-37 as approved by the Animal Research Ethics Board according to guidelines set by the Canadian Council on Animal Care. Animals were housed in a specific pathogen-free barrier facility under containment level 2 conditions and maintained on a 12 h:12 h light:dark cycle, which was maintained at a temperature of 21 °C and 30–50% humidity. Animals were randomly allocated to different groups and blinding was not deemed necessary. Pharmacokinetic evaluation of MKM-A was performed in immunocompetent, uninfected female ICR CD-1 mice (7–10 weeks of age; Envigo). Mice received a single subcutaneous dose of MKM-A (50 mg kg^−1^ in normal saline). The blood samples (0.3–0.4 ml) were collected by cardiac puncture under isoflurane anaesthesia at 0.25, 0.5, 1, 2, 4, 8 and 24 h post-dose (three animals per time point). Blood was drawn into K_2_EDTA tubes, kept on ice, centrifuged at 2,500*g* for 15 min at 4 °C, and plasma was stored at −70 °C for no longer than 1 week.

For LC–MS analysis, 100 µl plasma aliquots were mixed with 50 µl of 0.1% formic acid in acetonitrile to precipitate the proteins, and then 100 µl of 0.1% formic acid in water containing vancomycin (16 µg ml^−1^), as the internal standard, was added. Samples were vortexed for 5 min, centrifuged at 4,000 rpm for 5 min, and 1 µl of the supernatant was analysed on an Agilent 6550 qTOF coupled to a 1290 UPLC system. MKM-A and vancomycin were quantified using the ions [M + 4H]^4+^ (*m/z* 296.6723, retention time 6.007 min) and [M + 2H]^2+^ (*m/z* 724.7233, retention time 6.321 min), respectively. Chromatographic separation was achieved on a Luna Omega Polar C18 column (3 µm, 100 Å, 100 × 4.6 mm) at a flow rate of 0.5 ml min^−1^ and using the following gradient: 100% aqueous (0.1% formic acid) for 2 min, and then 100% aqueous to 95% acetonitrile over 9 min. Calibration standards (0.8–100 µg ml^−1^) were prepared in plasma and processed identically; the quantifiable linear range was 1.6–50 µg ml^−1^. Samples without detectable peaks or <75% of the lower limit of quantification were classified as being below the limit of quantification.

Peak areas were integrated using MassHunter Quantitation (v10.1). Plasma concentrations were plotted over time and pharmacokinetic parameters were calculated using Phoenix WinNonlin (Build 8.4.). Non-compartmental analysis used linear-scale data between 1–4 h, as values at 8 h and 24 h were below the limit of quantification. Parameters obtained included, elimination rate constant (λz), terminal half-life *t*_1/2_, C_max_ and AUC_(0–24 h)_.

### Statistics and reproducibility

Survival curves were analysed using a log-rank (Mantel–Cox) test. Statistical significance was defined as ****P  <  0.0001, as indicated in the figure legends. Data were compiled in Microsoft Excel, and statistical analyses were performed using GraphPad Prism (v.10.2.3 and v.10.5.0). All toeprinting assays were performed at least twice; shown gels are representative of at least two independent experiments that produced similar outcomes.

### Reporting summary

Further information on research design is available in the Nature Portfolio Reporting Summary linked to this article.

## Online content

Any methods, additional references, Nature Portfolio reporting summaries, source data, extended data, supplementary information, acknowledgements, peer review information; details of author contributions and competing interests; and statements of data and code availability are available at 10.1038/s41586-026-10589-2.

## Supplementary information

Supplementary Information Supplementary Information file contains Supplementary Figs. 1–39 and Supplementary Tables 1–9. These figures and tables contain data for chemical characterization of MKM variants, plasmid map, MKM BGC analysis, MKM-A plasma stability and pharmacokinetics, mutation analysis of MKM resistant strains, susceptibility of MKM to transporter mutants, list of bacterial strains, primers and mRNA sequences, Cryo-EM data and maps, raw blots

Reporting Summary

Supplementary Data Source Data Supplementary Fig. 35

Peer Review file

Supplementary Video 1 **MKM-A binding site on the 50S subunit**. The movie describes zoomed version of MKM-A interactions with E-site of the 50S subunit of the ribosome. Direct hydrogen-bonding interactions are shown as yellow dashed lines.

## Source data

Source Data Fig. 6

## Figures and Tables

**Extended Data Table 1:**
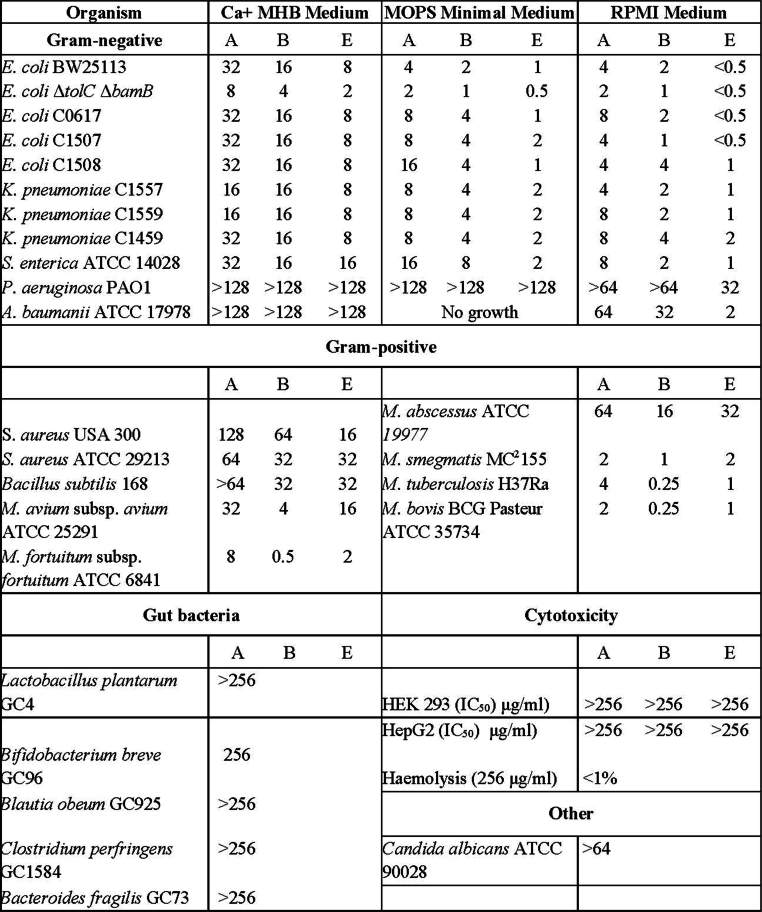
Antimicrobial spectrum and mammalian toxicity of manikomycins

MICs are represented in μg/ml of three variants of MKM: MKM-A, MKM-B, and MKM-E.

**Extended Data Table 2:**
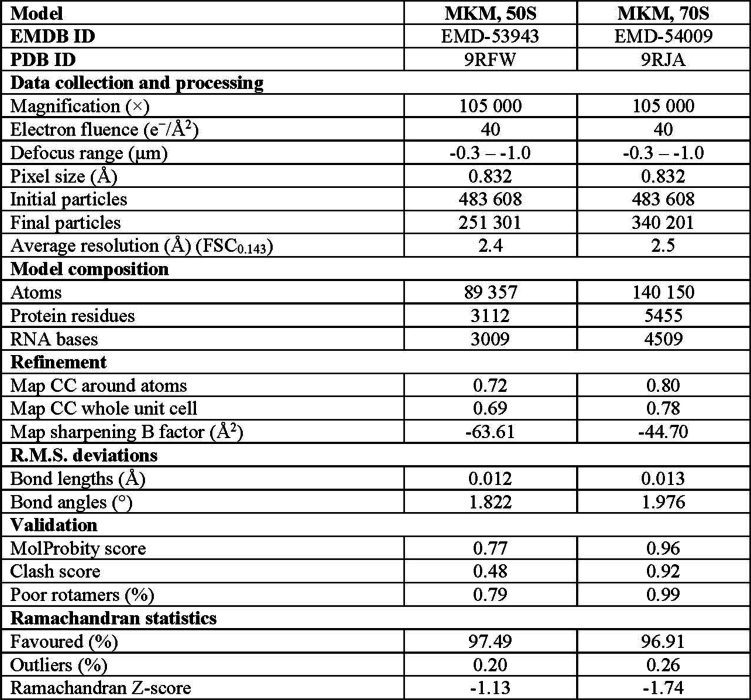
Cryo-EM data collection, modelling and refinement statistics

## Data Availability

Cryo-EM maps and molecular models were deposited in the Electron Microscopy Data Bank (EMDB) and Protein Data Bank (PDB) with accession codes EMD-54009 and PDB ID 9RJA (MKM–70S complex) and EMD-53943 and PDB ID 9RFW (MKM–50S complex), respectively. All previously published structures used in this work for structural comparisons were retrieved from the RCSB Protein Data Bank: PDB entries 2OTJ, 4U3U, 4U4R, 4U4Z, 5O61, 6SGC, 6SPG, 6ND6,7K00, 8AKN, 8GLP and 8P2G. This whole-genome shotgun project (*S. rimosus* WAC 7405) has been deposited at DDBJ/European Nucleotide Archive/GenBank under the accession JBPBLP000000000. The version described in this paper is version JBPBLP010000000. Sequencing data collected for ribosome profiling experiment were deposited in NCBI Sequence Read Archive (SRA) with BioProject ID PRJNA1265262. All other materials used in this study are available from corresponding authors upon request. Source data are provided with this paper.
