# Supplementary material for: A natural depsipeptide antibiotic binds the E-site of the bacterial ribosome
Source: Nature. 2026 Jun 3;655(8123):737–46. doi: 10.1038/s41586-026-10589-2 (PMC13372681; doi:10.1038/s41586-026-10589-2)
Supplement: Supplementary file 1 — Supplementary Information file contains Supplementary Figs. 1–39 and Supplementary Tables 1–9. These figures and tables contain data for chemical characterization of MKM variants, plasmid map, MKM BGC analysis, MKM-A plasma stability and pharmacokinetics, mutation analysis of MKM resistant strains, susceptibility of MKM to transporter mutants, list of bacterial strains, primers and mRNA sequences, Cryo-EM data and maps, raw blots [file 41586_2026_10589_MOESM1_ESM.pdf]

nature portfolio

https://doi.org/10.1038/s41586-026-10589-2

Supplementary information

A natural depsipeptide antibiotic binds the E-site of the bacterial ribosome

In the format provided by the authors and unedited

Supplementary Information

A Natural Depsipeptide Antibiotic binds the E-site of the Bacterial Ribosome

Manpreet Kaur[1,2,3,†], Dmitrii Y. Travin[4,5,†], Max J. Berger[6,†], Manoj Jangra[1,2,3], Martino Morici[6], Haaris A. Safdari[6], Dorota Klepacki[4,5], Wenliang Wang[1,2,3], Michael Cook[1,2,3], Sommer Chou[1,2,3], Allison. K. Guitor[1,2,3], Kalinka Koteva[1,2,3], Min Xu[1,2,3], Linda Ejim[1,2,3], Aline Fiebig[1,2,3], Yeganeh Yousefi[1,2,3], Brian K. Coombes[1,2,3], Lesley Macneil[1,2,3], Nora Vázquez-Laslop[4,5], Alexander S. Mankin[4,5, #], Daniel N. Wilson[6, #], Gerard D. Wright[1,2,3, #]

[1] David Braley Centre for Antibiotics Discovery, McMaster University, Hamilton, Ontario, Canada.
[2] M.G. DeGroote Institute for Infectious Disease Research, McMaster University, Hamilton, Ontario, Canada.
[3] Department of Biochemistry and Biomedical Sciences, McMaster University, Hamilton, Ontario, Canada.
[4] Department of Pharmaceutical Sciences, University of Illinois at Chicago, Chicago, IL 60607, USA.
[5] Center for Biomolecular Sciences, University of Illinois at Chicago, Chicago, IL 60607, USA.
[6] Institute for Biochemistry and Molecular Biology, University of Hamburg, Martin-Luther-King-Platz 6, 20146 Hamburg, Germany.
[†] these authors contributed equally
[#] corresponding authors

[#] correspondence to: wrightge@mcmaster.ca, shura@uic.edu, daniel.wilson@uni-hamburg.de

Keywords: Depsipeptide; NRPS; ribosome; antibiotic; E site; translocation; gram-negative bacteria; antimicrobial resistance.

Table of Contents

SUPPLEMENTARY DATA FIGURES 4

Supplementary Fig. 1 | 1H NMR spectrum of MKM-A 4

Supplementary Fig. 2 | ^{13}C DEPTQ NMR spectrum of MKM-A 5

Supplementary Fig. 3 | ^{1}H-^{1}H COSY NMR spectrum of MKM-A 6

Supplementary Fig. 4 | ^{1}H-^{13}C HSQC NMR spectrum of MKM-A 7

Supplementary Fig. 5 | ^{1}H-^{13}C HMBC NMR spectrum of MKM-A 8

Supplementary Fig. 6 | TOCSY- HSQC NMR spectrum of MKM-A 9

Supplementary Fig. 7 | NOESY-NMR spectrum of MKM-A 10

Supplementary Fig. 8 | NMR assignment of MKM-A in deuterium oxide (D2O) 11

Supplementary Fig. 9 | ^{1}H NMR spectrum of MKM-B 12

Supplementary Fig. 10 | ^{13}C DEPTQ NMR spectrum of MKM-B 13

Supplementary Fig. 11 | ^{1}H-^{1}H COSY NMR spectrum of MKM-B 14

Supplementary Fig. 12 | ^{1}H-^{13}C HSQC NMR spectrum of MKM-B 15

Supplementary Fig. 13 | ^{1}H-^{13}C HMBC NMR spectrum of MKM-B 16

Supplementary Fig. 14 | TOCSY- HSQC NMR spectrum of MKM-B 17

Supplementary Fig. 15 | NOESY-NMR spectrum of MKM-B 18

Supplementary Fig. 16 | ^{1}H NMR spectrum of MKM-E 19

Supplementary Fig. 17 | ^{13}C DEPTQ NMR spectrum of MKM-E 20

Supplementary Fig. 18 | ^{1}H-^{1}H COSY NMR spectrum of MKM-E 21

Supplementary Fig. 19 | ^{1}H-^{13}C HSQC NMR spectrum of MKM-E 22

Supplementary Fig. 20 | ^{1}H-^{13}C HMBC NMR spectrum of MKM-E 23

Supplementary Fig. 21 | TOCSY- HSQC NMR spectrum of MKM-E 24

Supplementary Fig. 22 | NOESY-NMR spectrum of MKM-E 25

Supplementary Fig. 23 | Extracted Ion Chromatogram (EIC) of acid-hydrolyzed MKM variants treated with Marfey's reagent. 26

Supplementary Fig. 24 | Mass spectrometry analysis of MKM variants. 27

Supplementary Fig. 25 | (a) Yield proportions of the MKM variants. (b) HPLC chromatogram displaying the peaks corresponding to the individual MKM variants... 28

Supplementary Fig. 26. | In silico sorting scheme of the E. coli MKM complex 29

Supplementary Fig. 27. | Angular distribution and local resolution of MKM cryo-EM maps. 31

Supplementary Fig. 28. | Secondary binding site of MKM on the 50S subunit. 32

Supplementary Fig. 29. | Interaction of MKM in the E-site of the 50S subunit. 33

Supplementary Fig. 30 | tRNA identity and codon-anticodon interaction. 34

Supplementary Fig. 31 | BLAST analysis of the MKM BGC 35

Supplementary Fig. 32 | Stachelhaus code analysis. 36

Supplementary Fig. 33 | Conserved binding mode of MKM congeners 37

Supplementary Fig. 34 | Map of the pCGW vector with MKM BGC. 38

Supplementary Fig. 35| Plasma stability and pharmacokinetic characterization of MKM-A. 40

Supplementary Fig. 36 | Role of peptide uptake transporters SbmA and YejABEF in MKM susceptibility in E. coli. 41

Supplementary Fig. 37 | Raw blots for toeprinting experiments used in Fig. 4b,c,f. 42

Supplementary Fig. 38 | Raw blot for toeprinting experiment used in Fig. 5b. 43

Supplementary Fig. 39 | Raw blots for toeprinting experiments used in Extended data Fig. 4 a, c. 44

SUPPLEMENTARY TABLES 45

Supplementary Table 1 | ^{1}H and ^{13}C NMR assignment of MKM-A 45

Supplementary Table 2 | ^{1}H and ^{13}C NMR assignment of MKM-B 46

Supplementary Table 3 | ^{1}H and ^{13}C NMR assignment of MKM-E 47

Supplementary Table 4 | Calculated and Observed Masses of MKM variants. 48

Supplementary Table 5 | Proposed functions of proteins encoded by the MKM BGC (accession no: JBPBLP010000002.1) using antiSMASH v6.0.0[3] and antiSMASH v8.0.0[4] 49

Supplementary Table 6 | Mutations in MKM-resistant E. coli mutants from serial passaging 50

Supplementary Table 7 | Bacterial strains and plasmids used in the study. 51

Supplementary Table 8 | Oligonucleotide primers used in this study. 53

Supplementary Table 9 | mRNA templates used in the study. 54

SUPPLEMENTARY DATA FIGURES

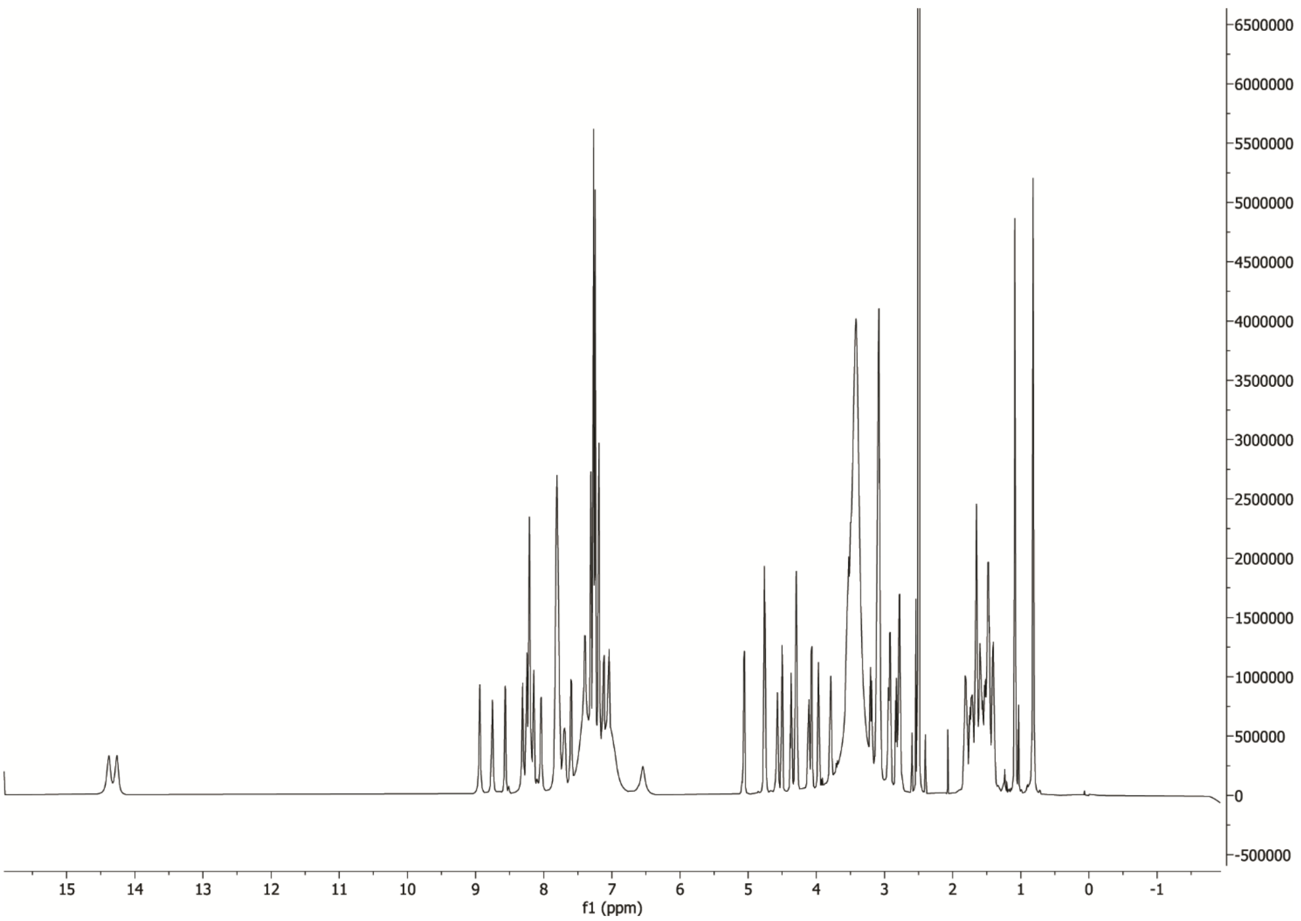

Supplementary Fig. 1 | 1H NMR spectrum of MKM-A

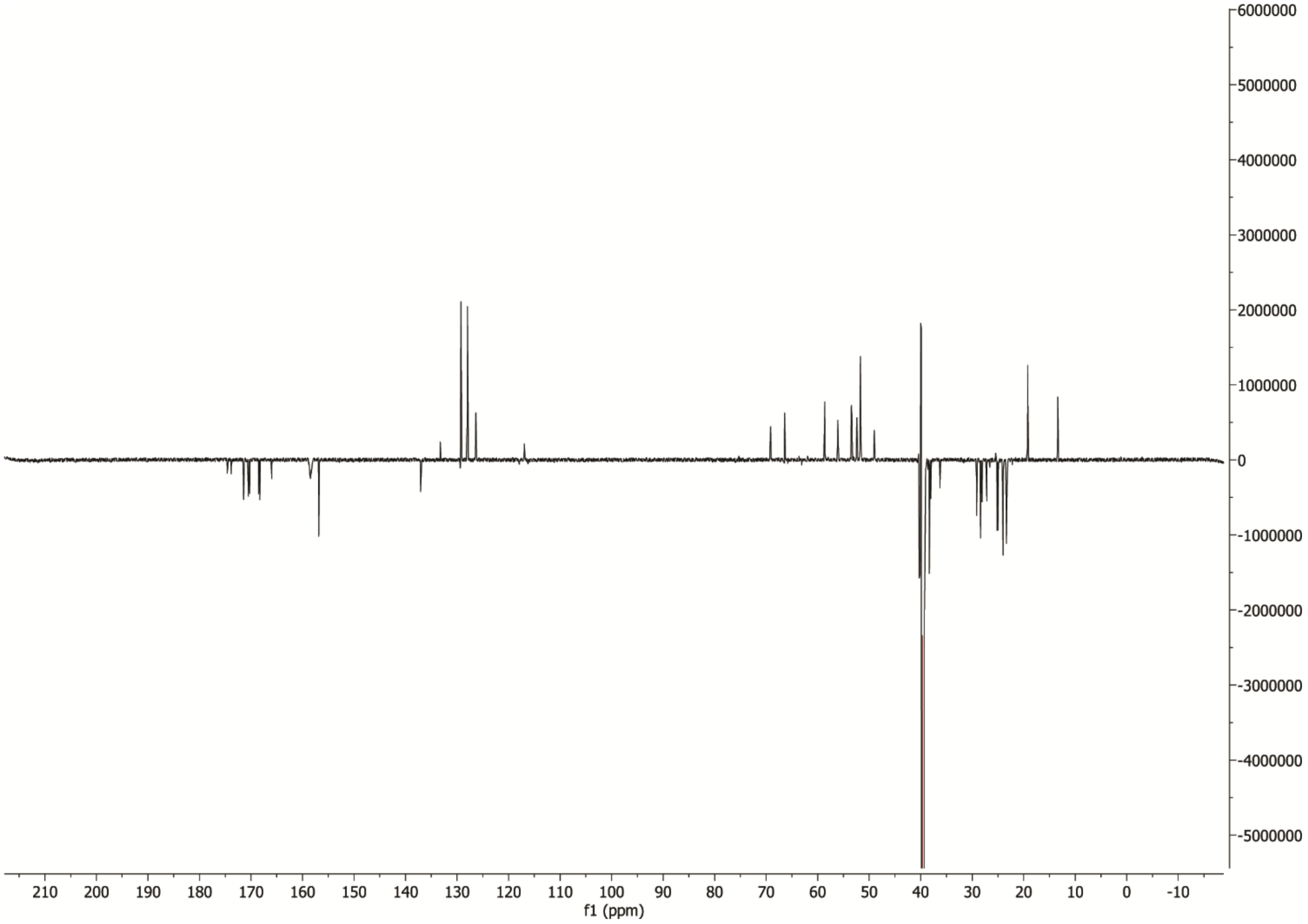

Supplementary Fig. 2 | ^{13}C DEPTQ NMR spectrum of MKM-A

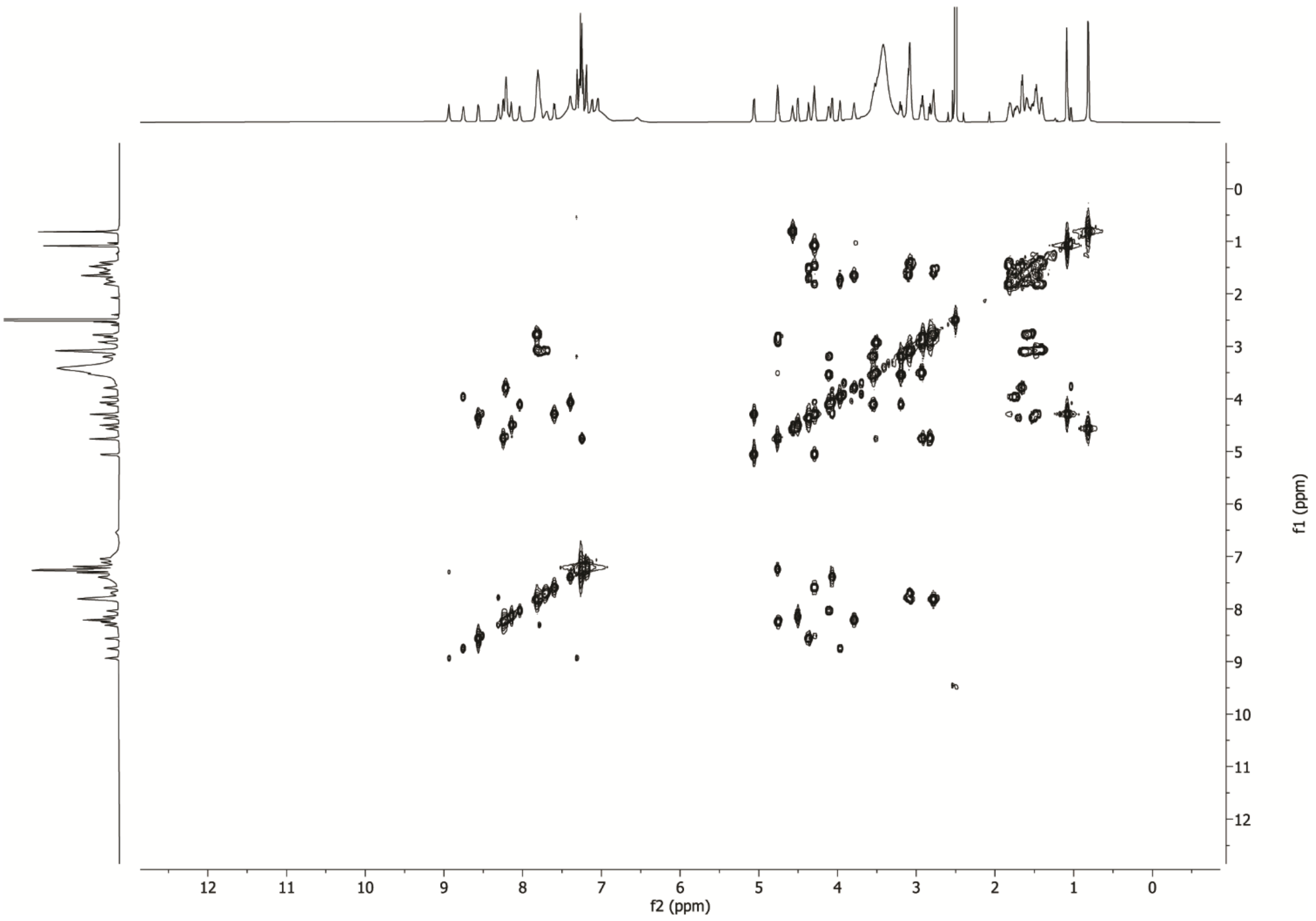

Supplementary Fig. 3 | ^{1}H-^{1}H COSY NMR spectrum of MKM-A

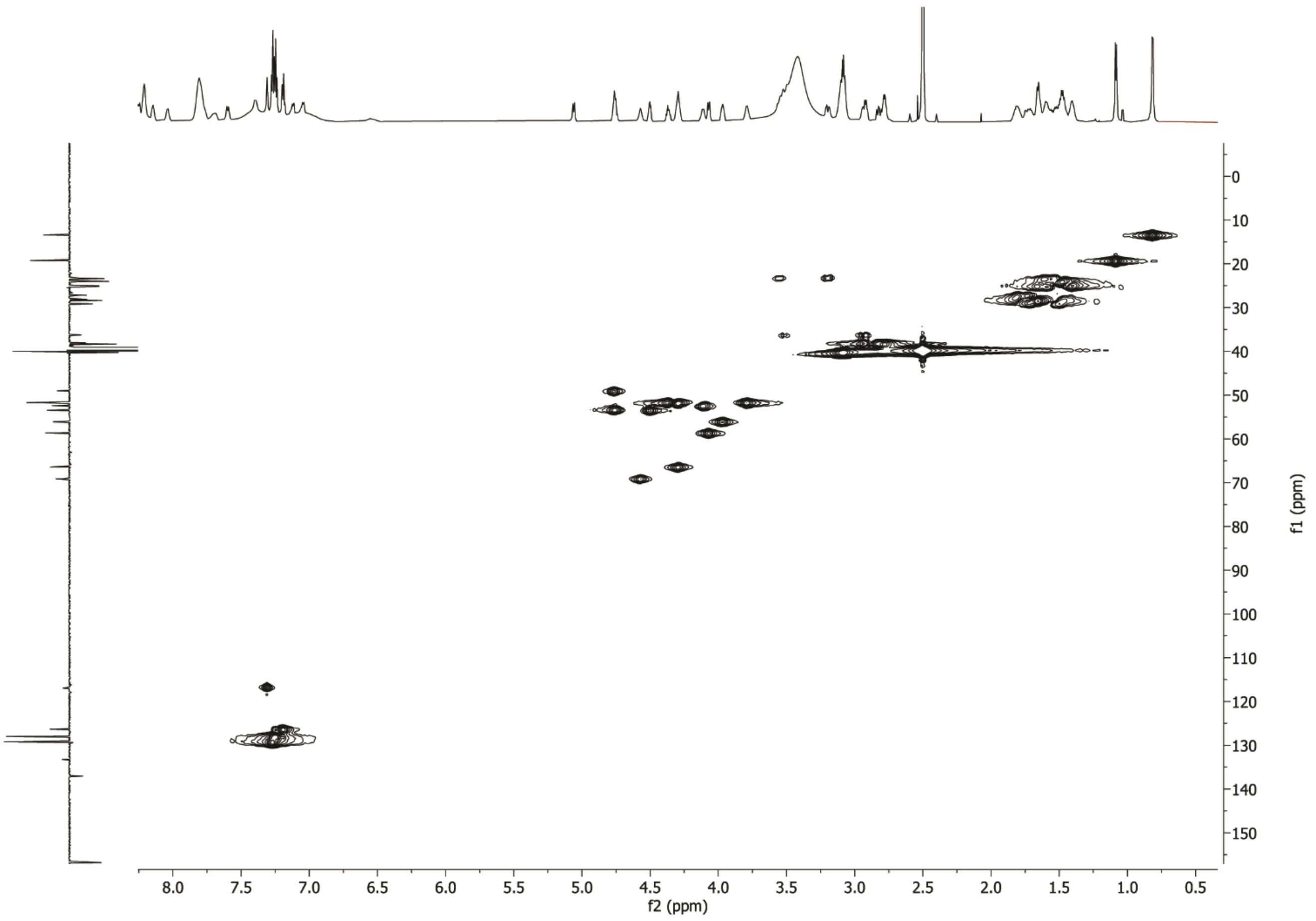

Supplementary Fig. 4 | ^{1}H-^{13}C HSQC NMR spectrum of MKM-A

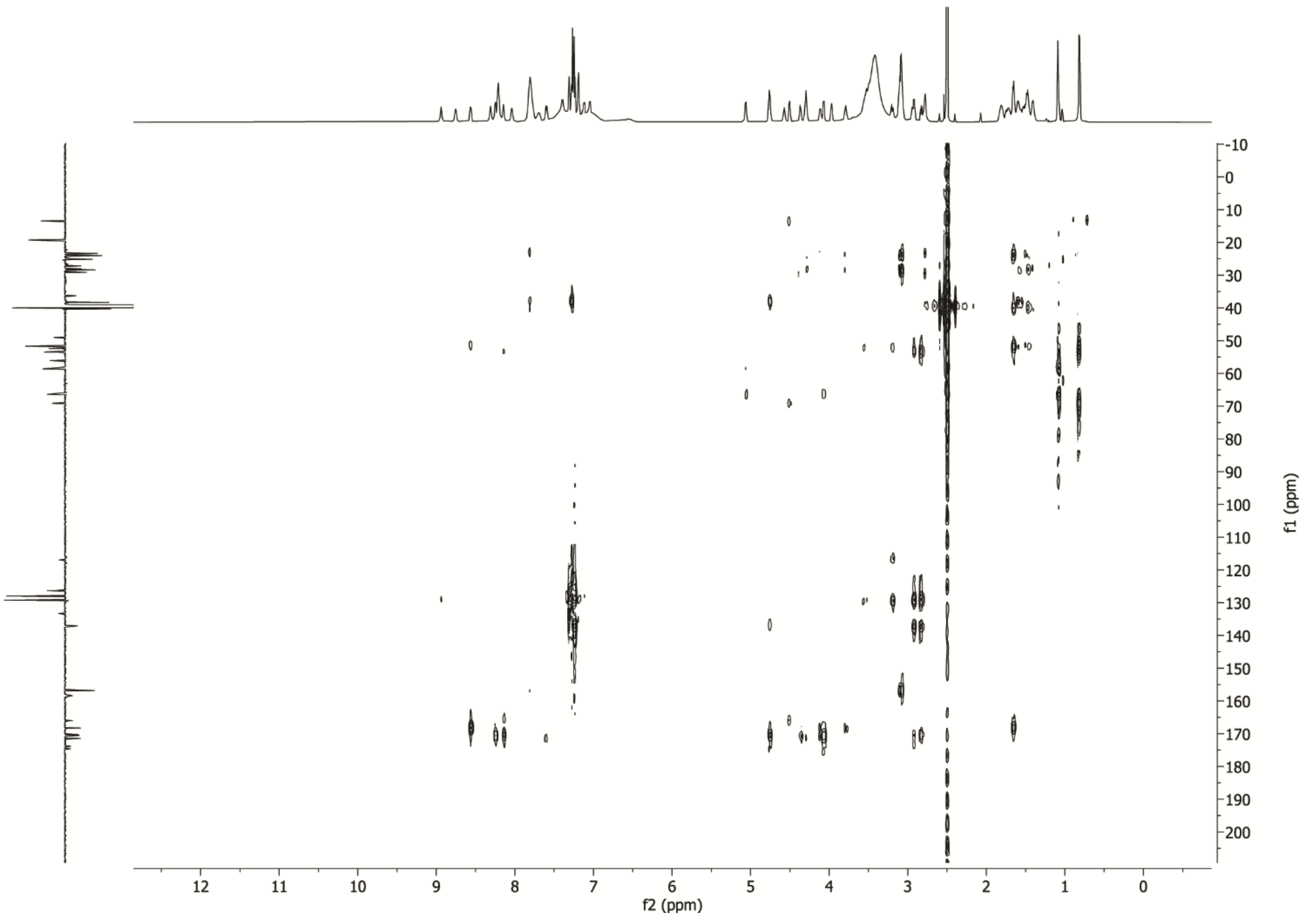

Supplementary Fig. 5 | ^{1}H-^{13}C HMBC NMR spectrum of MKM-A

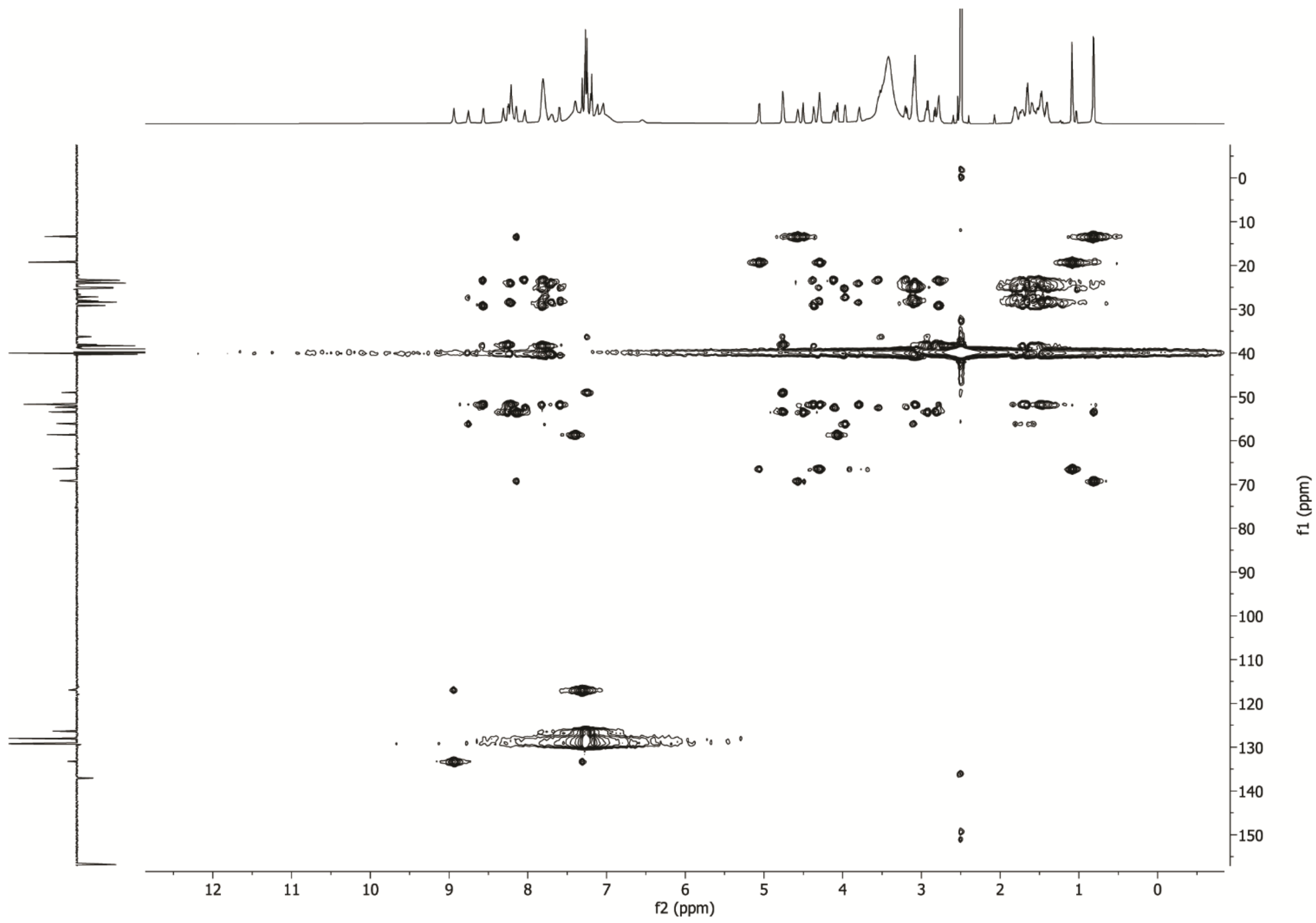

Supplementary Fig. 6 | TOCSY- HSQC NMR spectrum of MKM-A

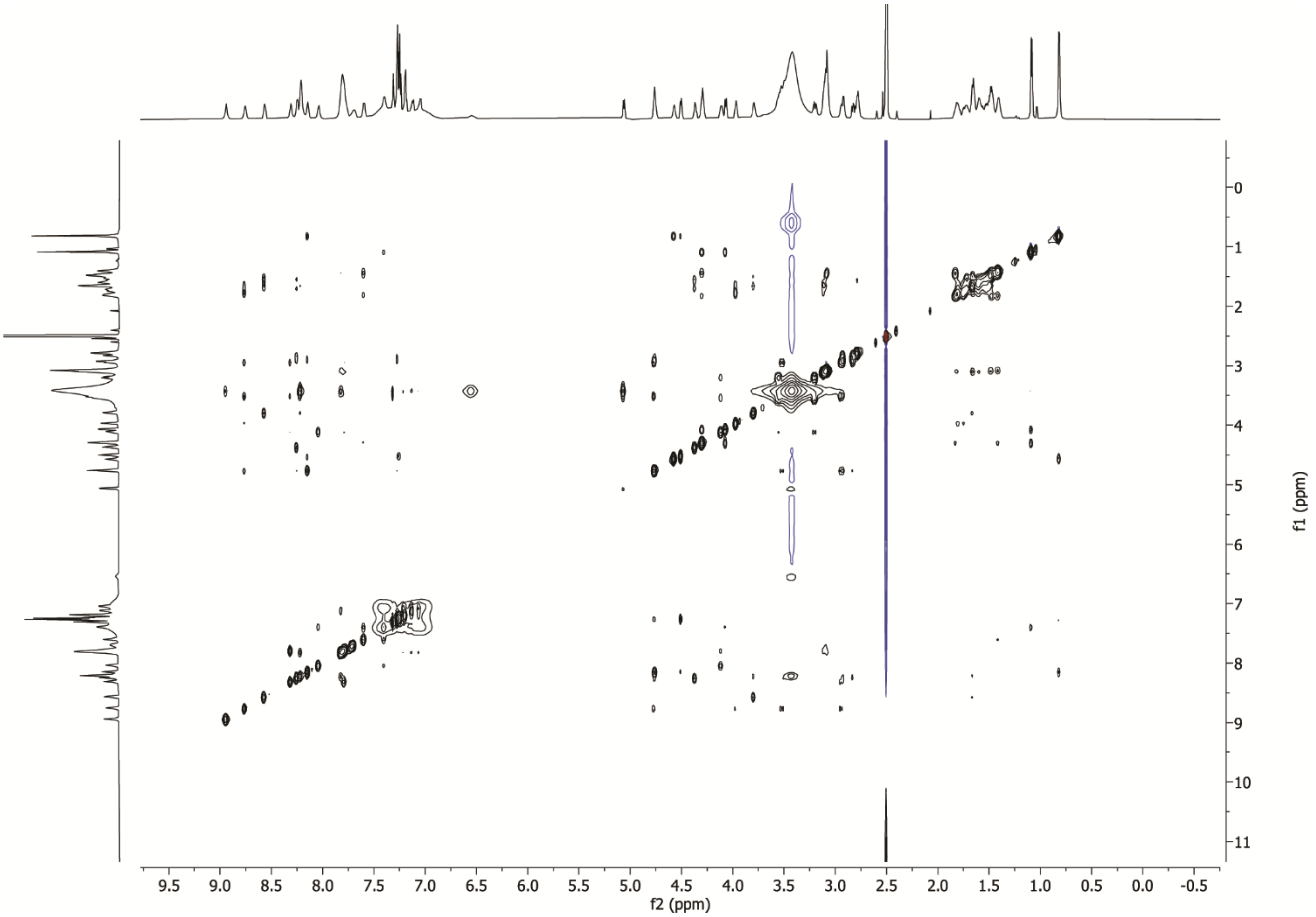

Supplementary Fig. 7 | NOESY-NMR spectrum of MKM-A

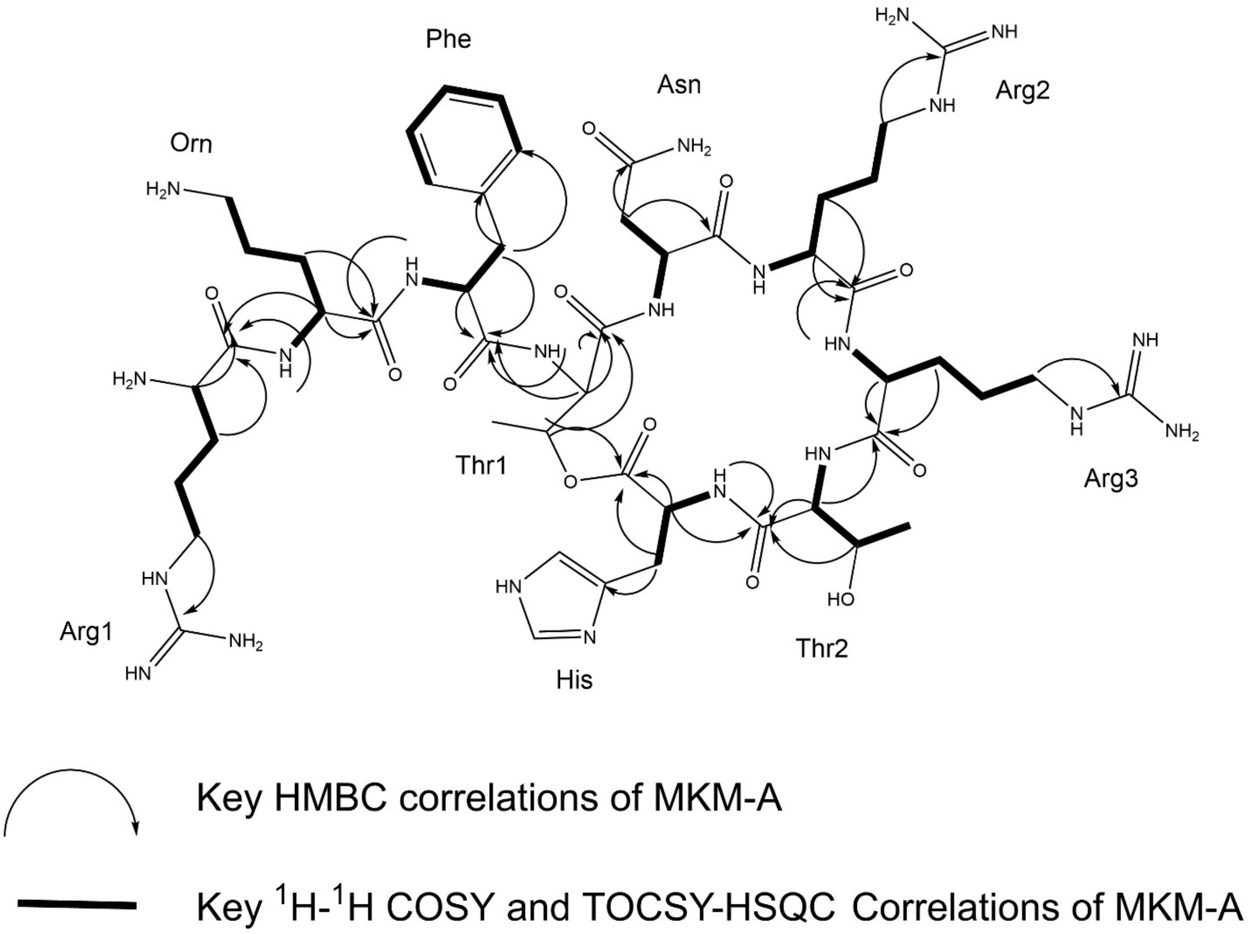

Supplementary Fig. 8 | NMR assignment of MKM-A in deuterium oxide (D2O). ^{1}H and ^{13}C NMR chemical shifts of various amino acids in MKM. The key correlations of MKM-A relevant to NMR spectra are shown.

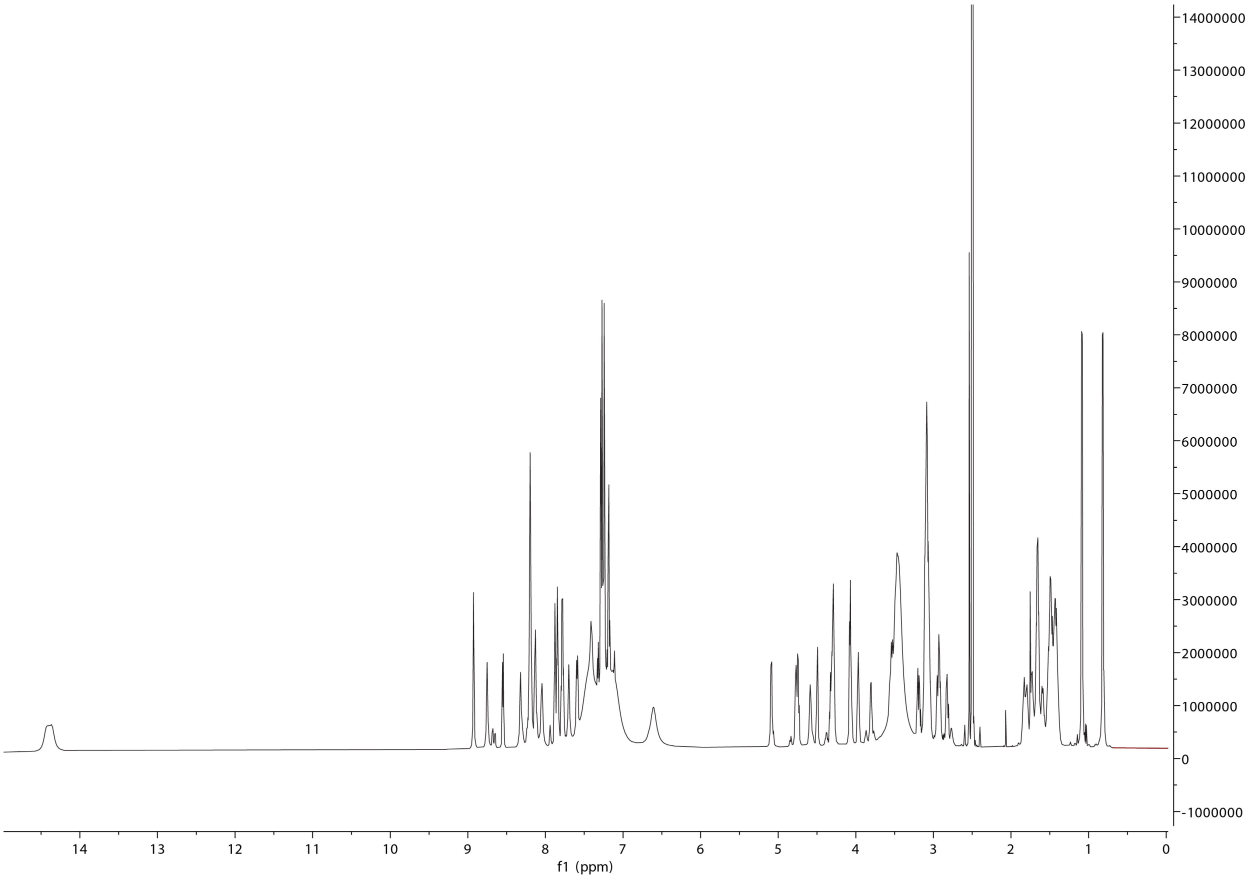

Supplementary Fig. 9 | ^{1}H NMR spectrum of MKM-B

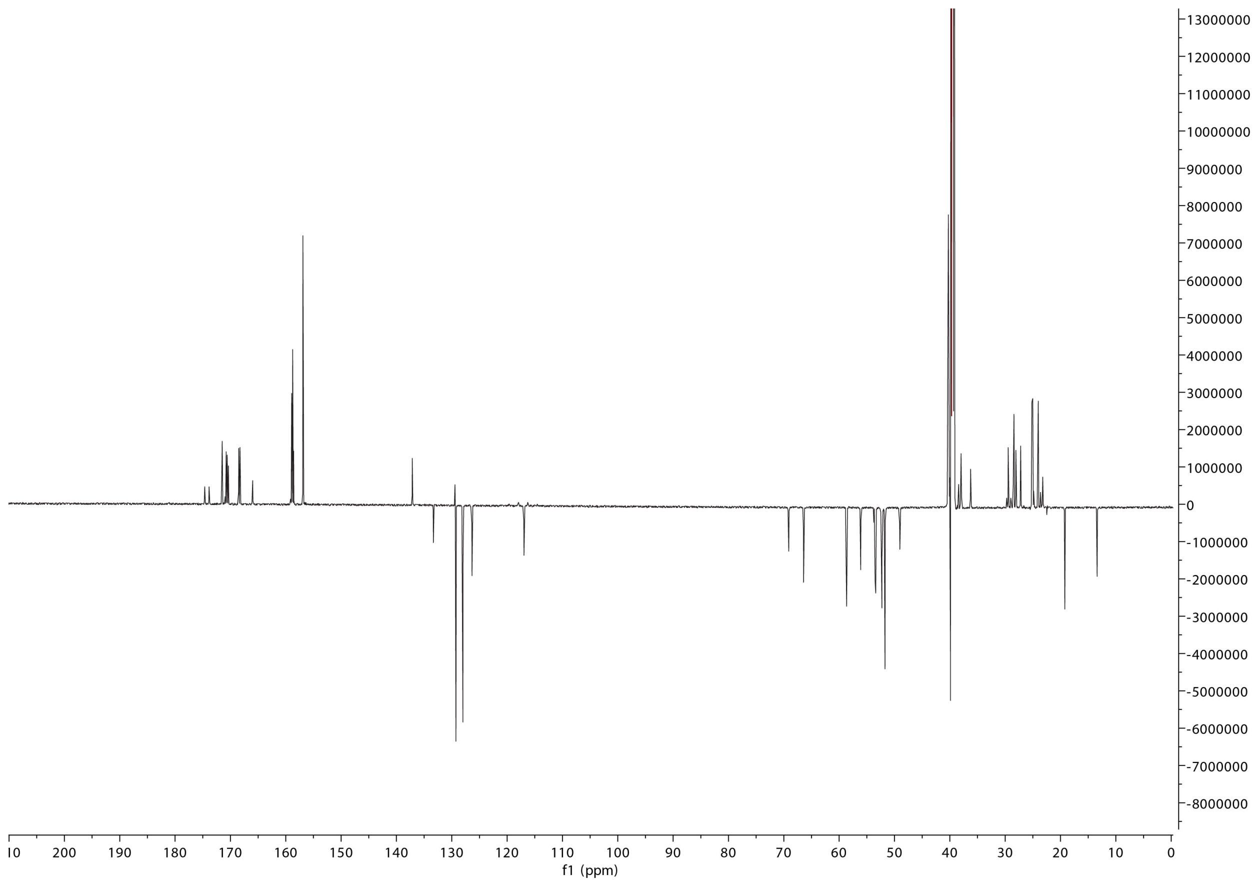

Supplementary Fig. 10 | ^{13}C DEPTQ NMR spectrum of MKM-B

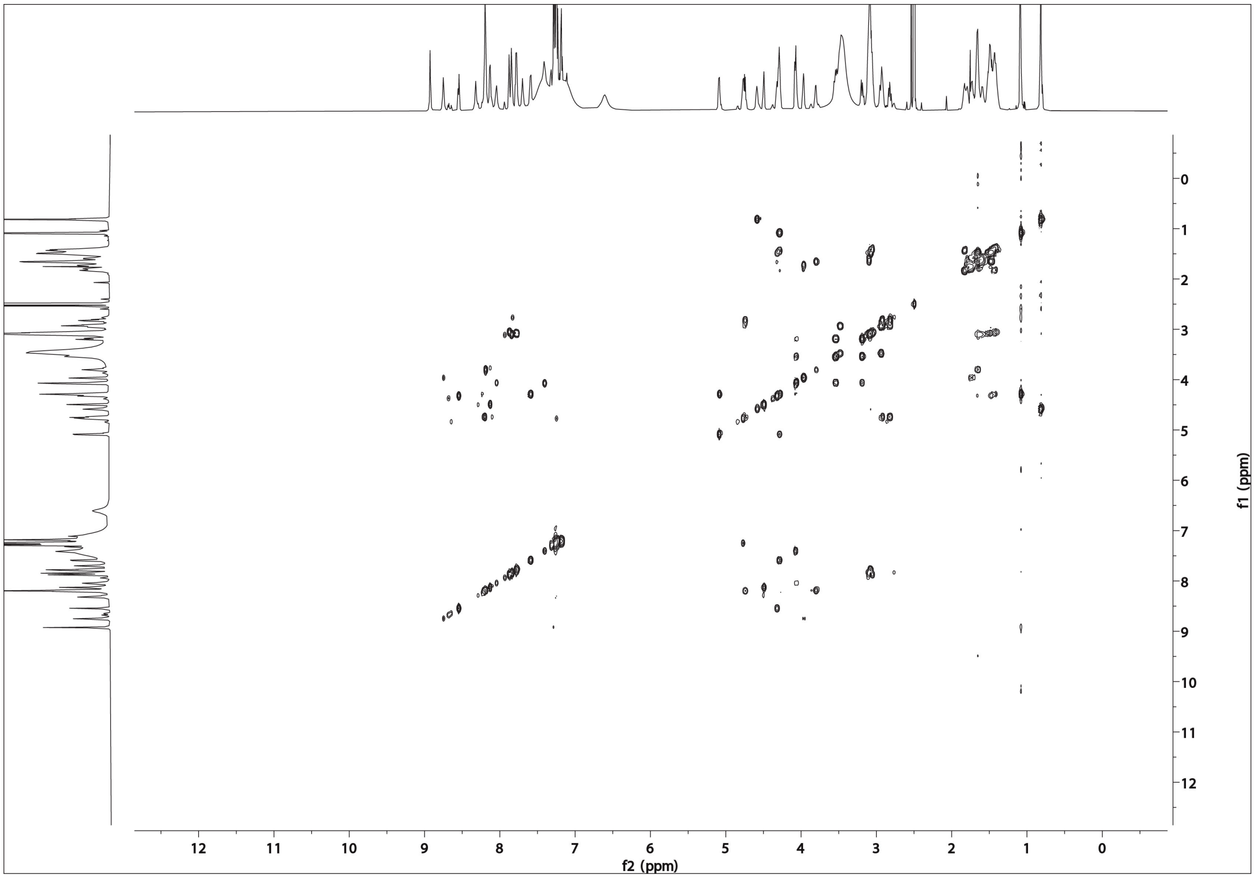

Supplementary Fig. 11 | ^1H-^1H COSY NMR spectrum of MKM-B

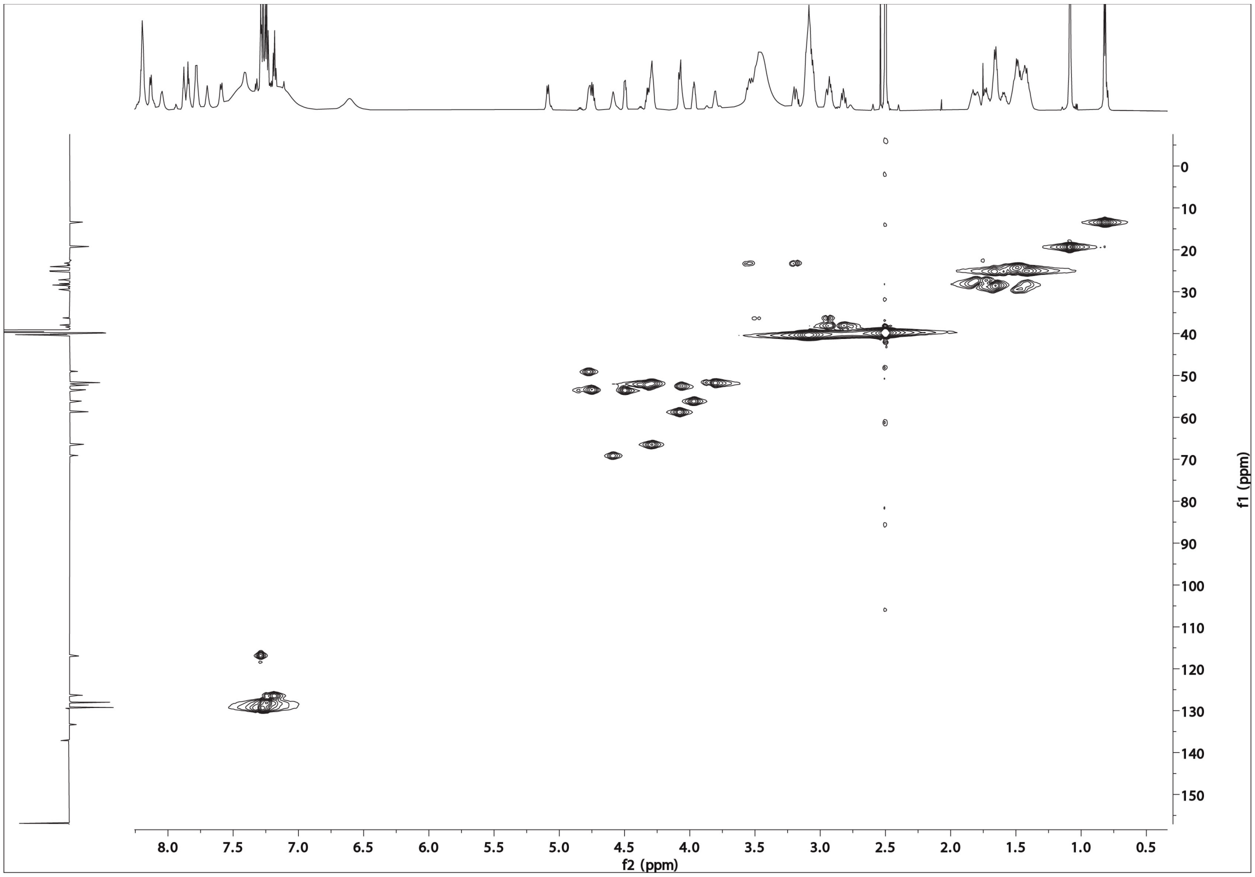

Supplementary Fig. 12 | ^{1}H-^{13}C HSQC NMR spectrum of MKM-B

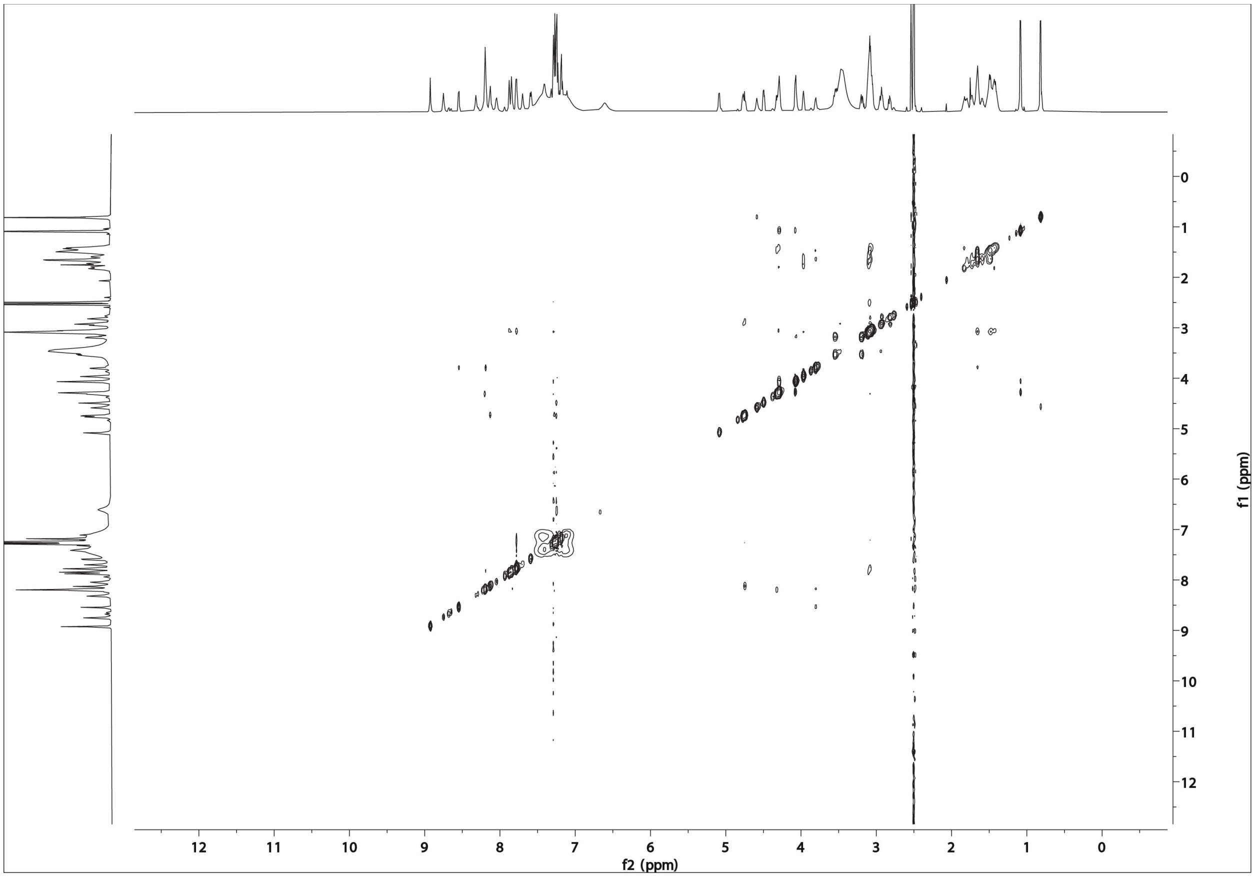

Supplementary Fig. 13 | ^{1}H-^{13}C HMBC NMR spectrum of MKM-B

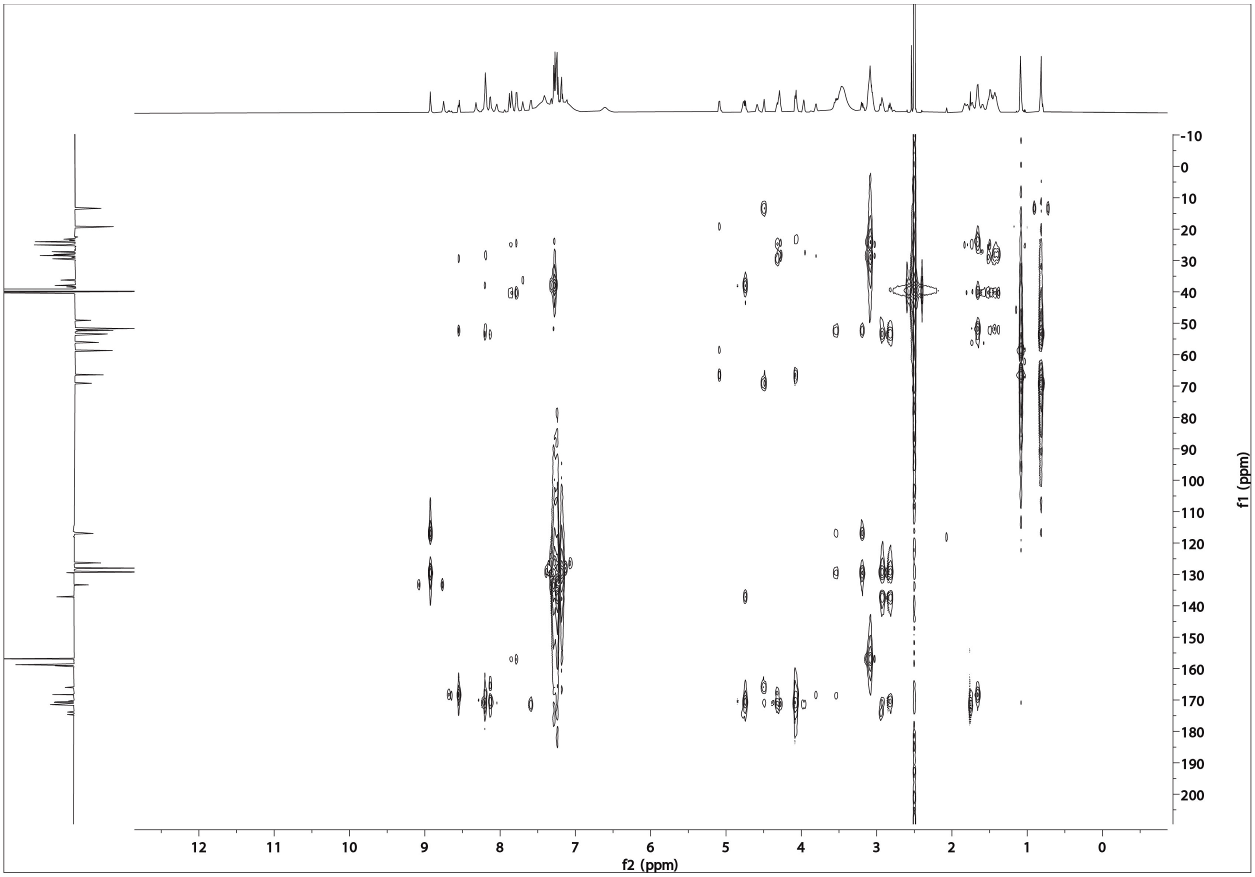

Supplementary Fig. 14 | TOCSY- HSQC NMR spectrum of MKM-B

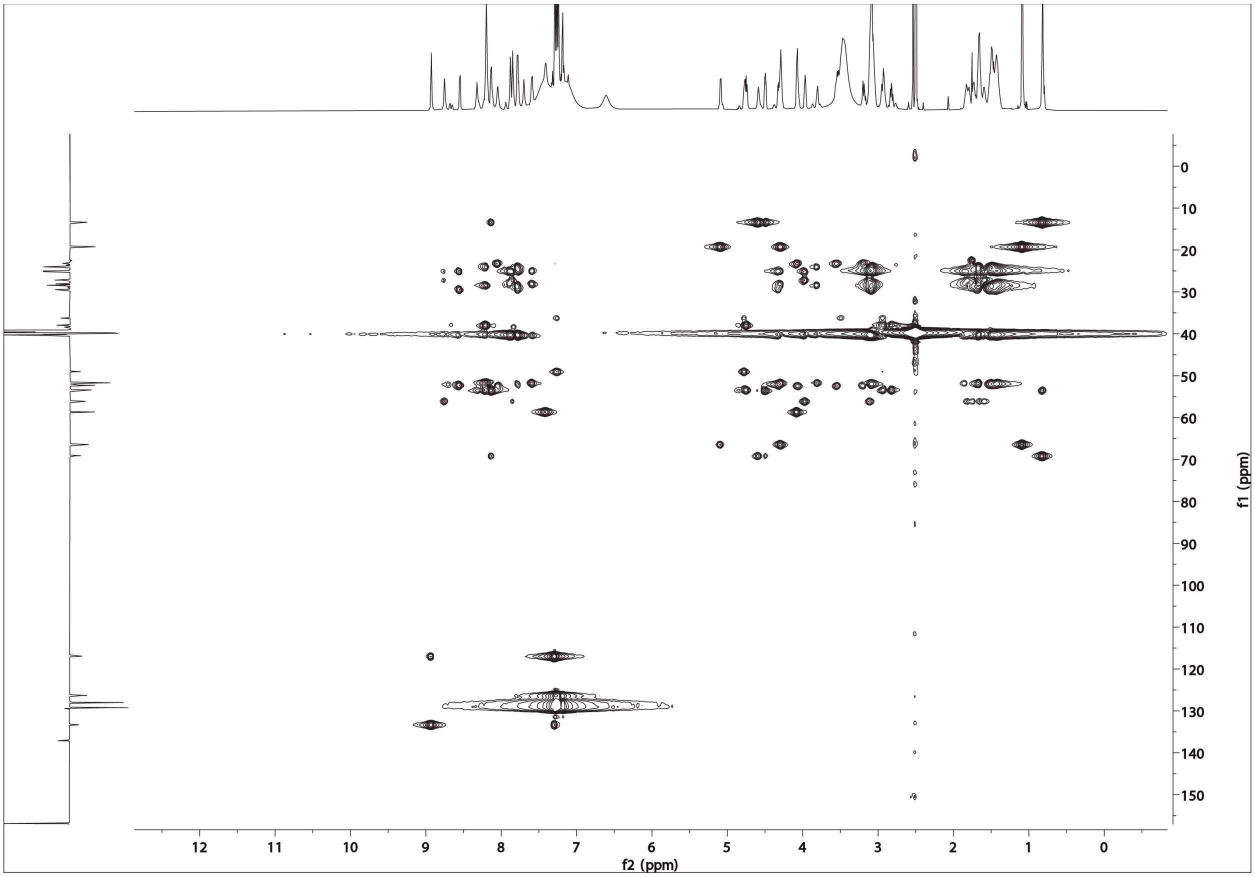

Supplementary Fig. 15 | NOESY-NMR spectrum of MKM-B

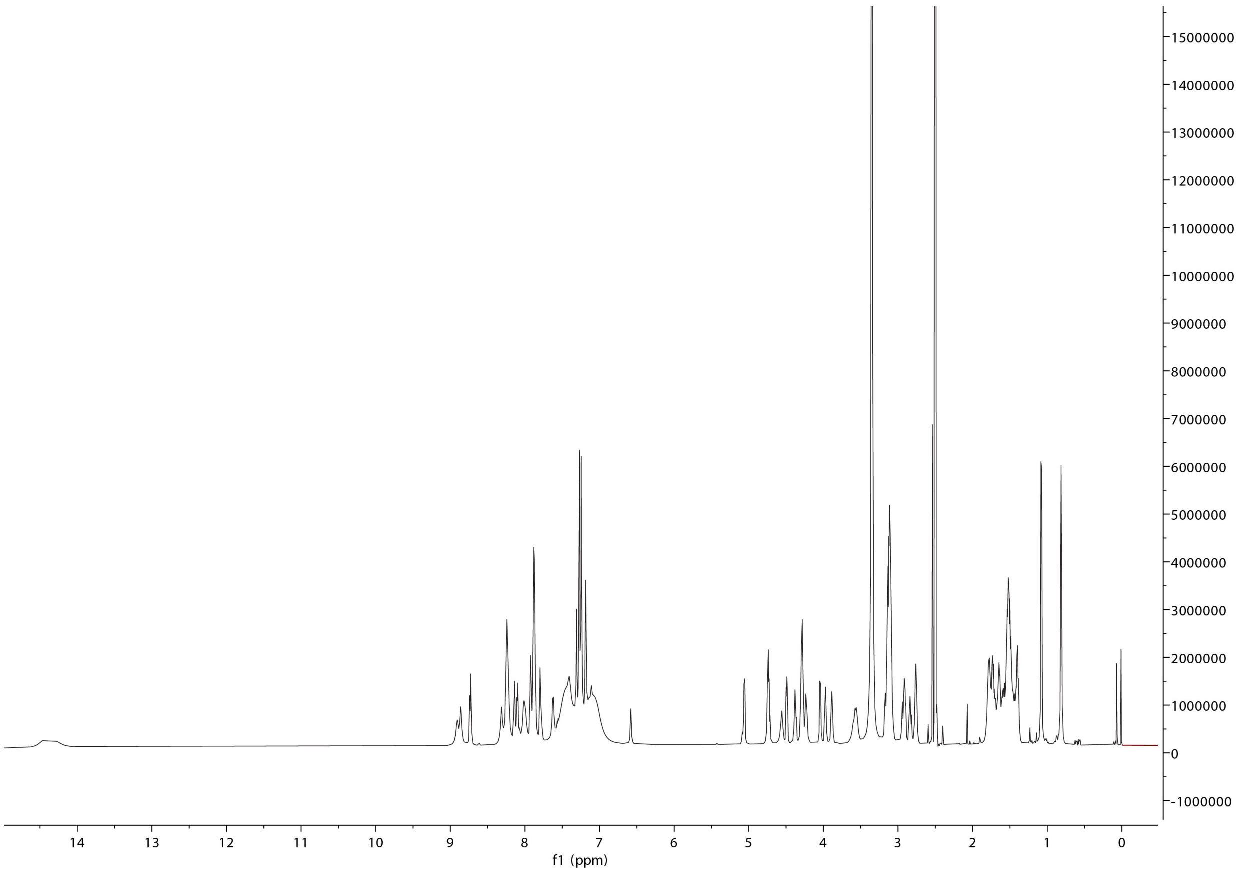

Supplementary Fig. 16 | ^{1}H NMR spectrum of MKM-E

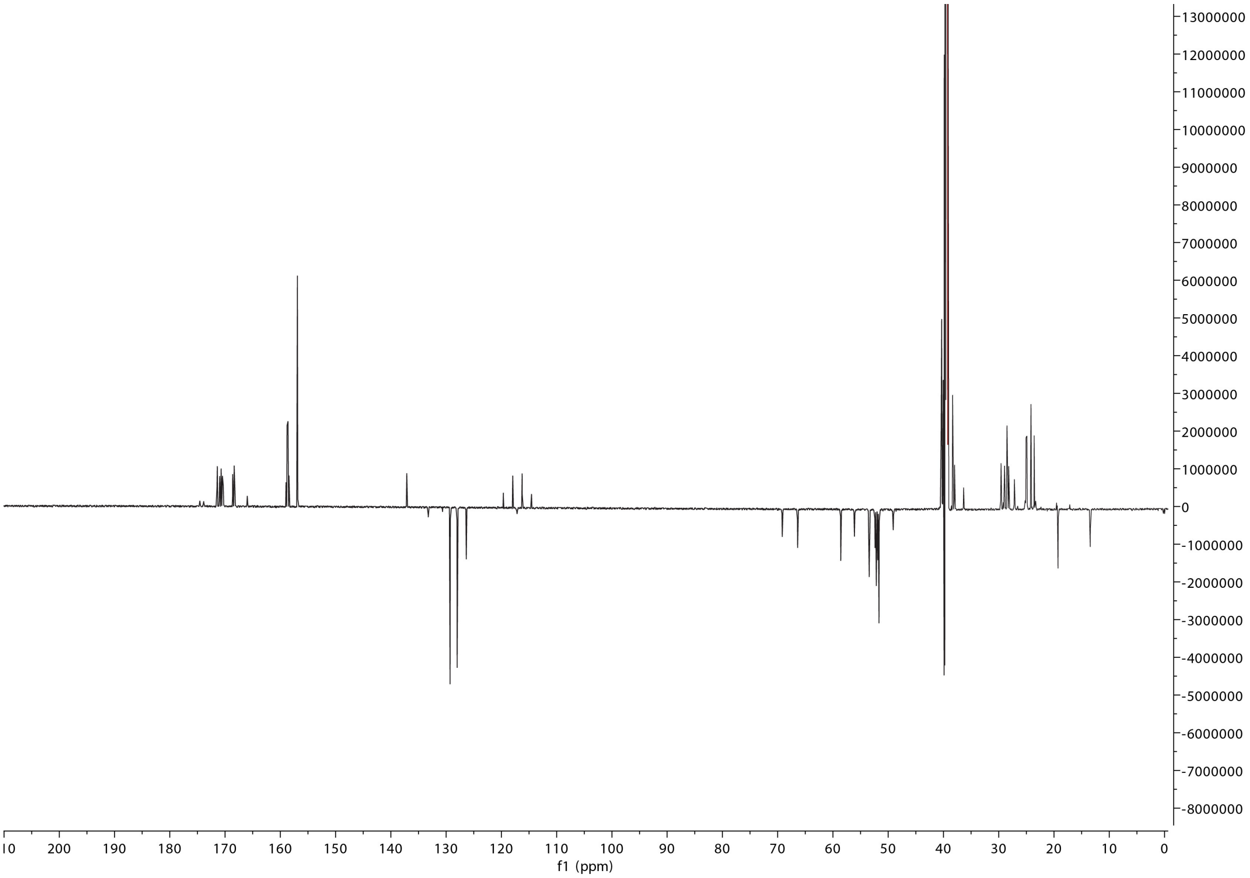

Supplementary Fig. 17 | ^{13}C DEPTQ NMR spectrum of MKM-E

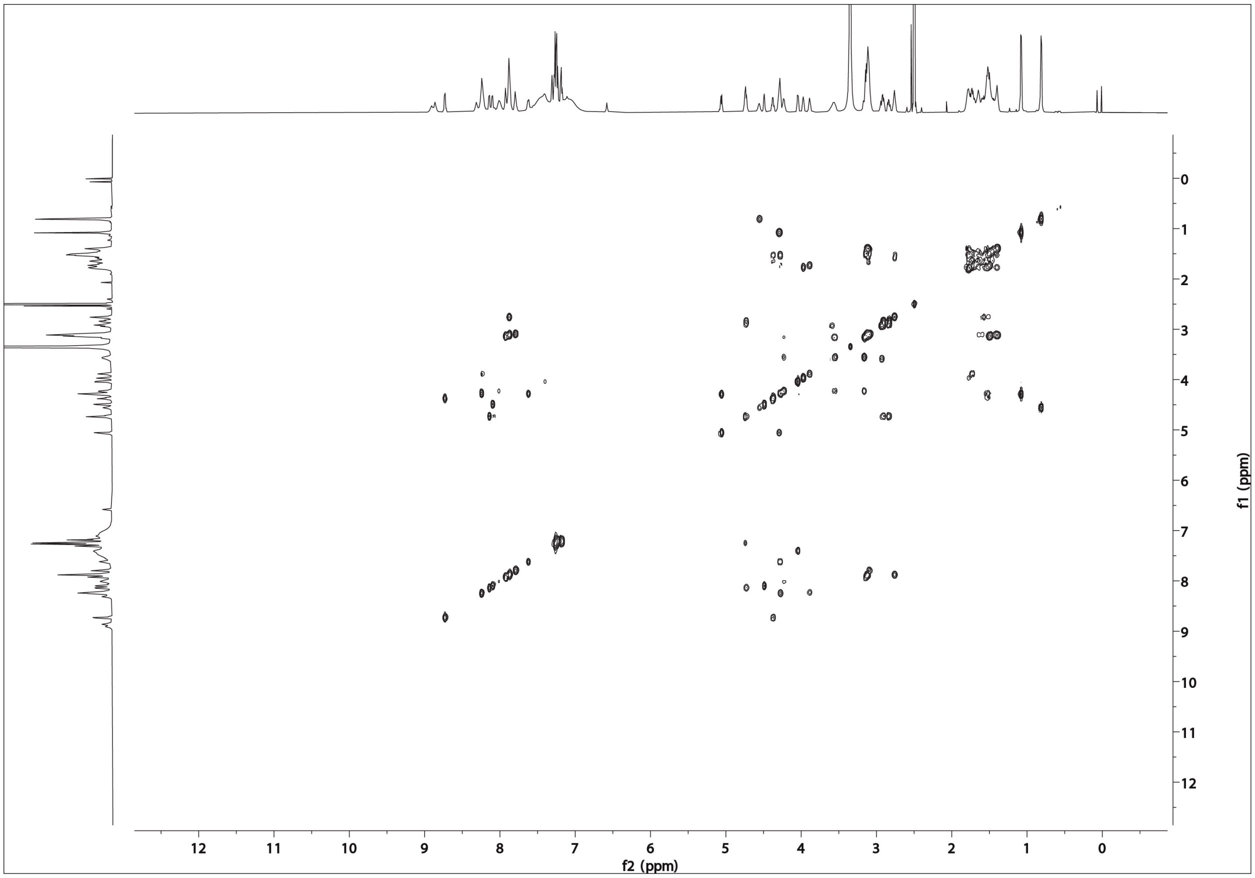

Supplementary Fig. 18 | ^{1}H-^{1}H COSY NMR spectrum of MKM-E

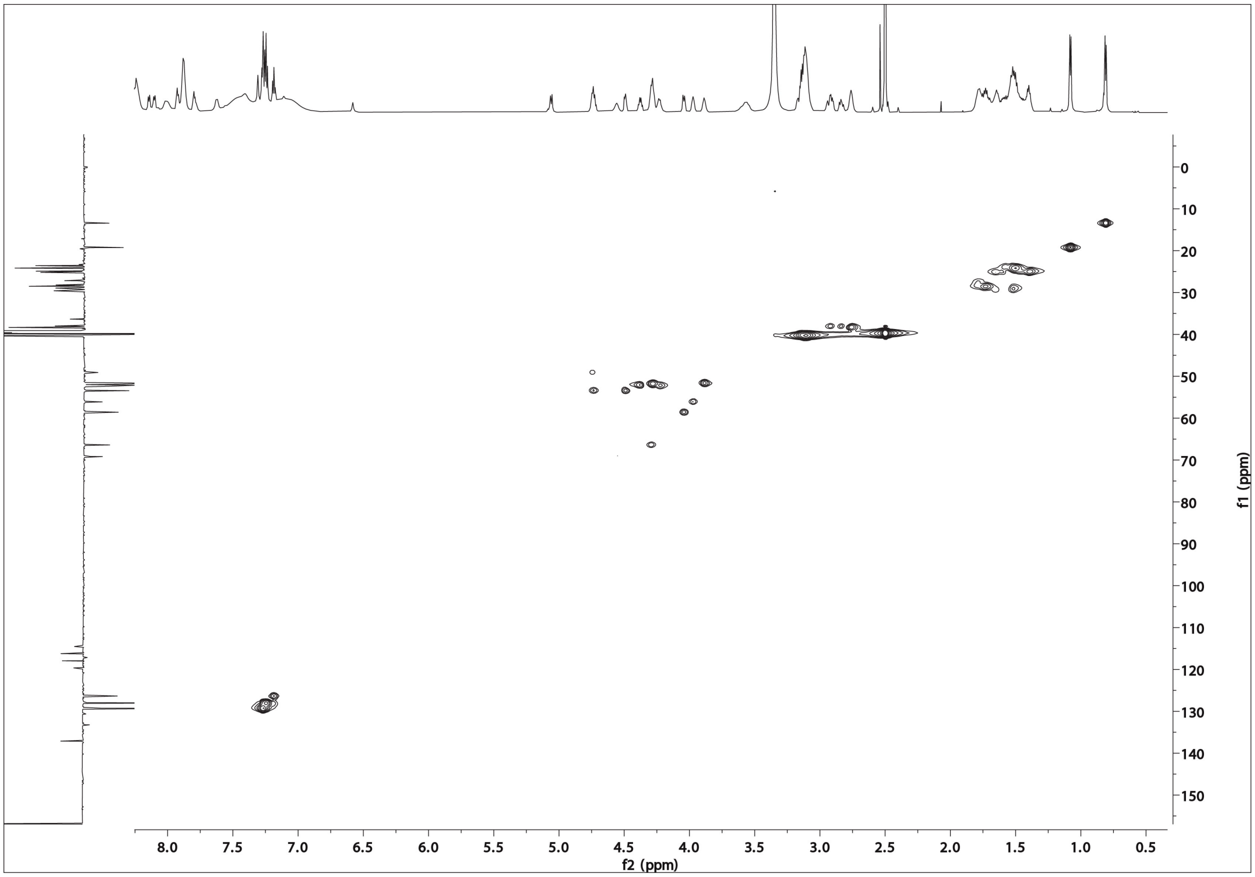

Supplementary Fig. 19 | ^{1}H-^{13}C HSQC NMR spectrum of MKM-E

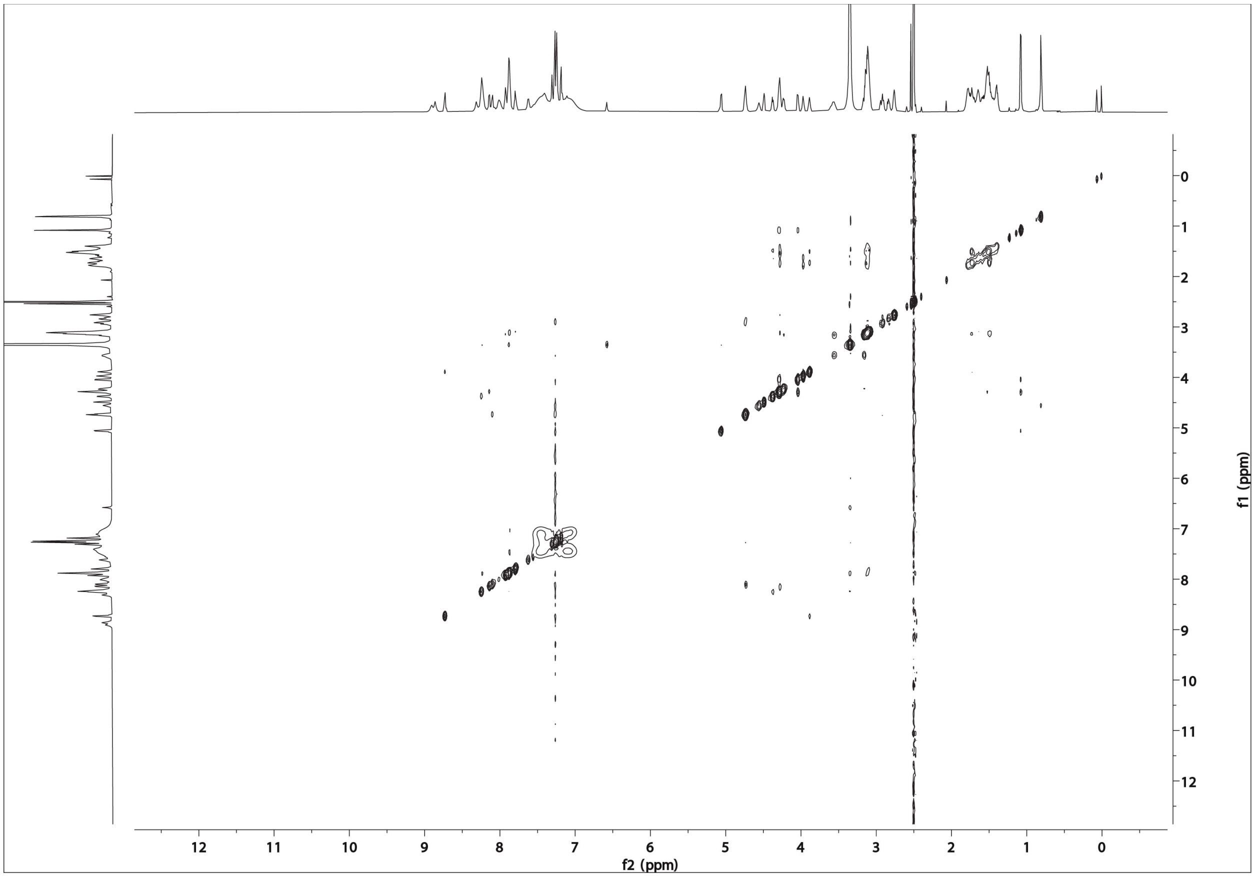

Supplementary Fig. 20 | ^{1}H-^{13}C HMBC NMR spectrum of MKM-E

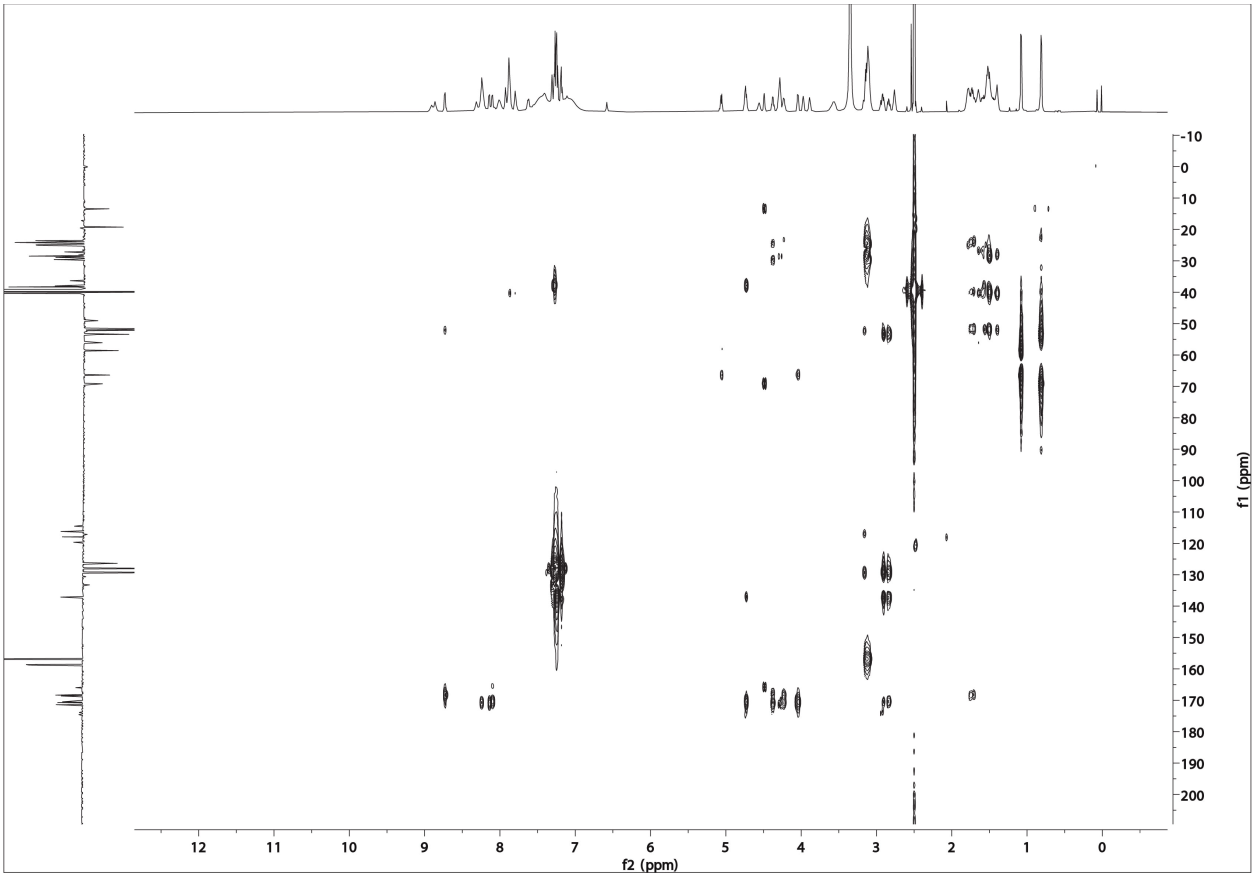

Supplementary Fig. 21 | TOCSY- HSQC NMR spectrum of MKM-E

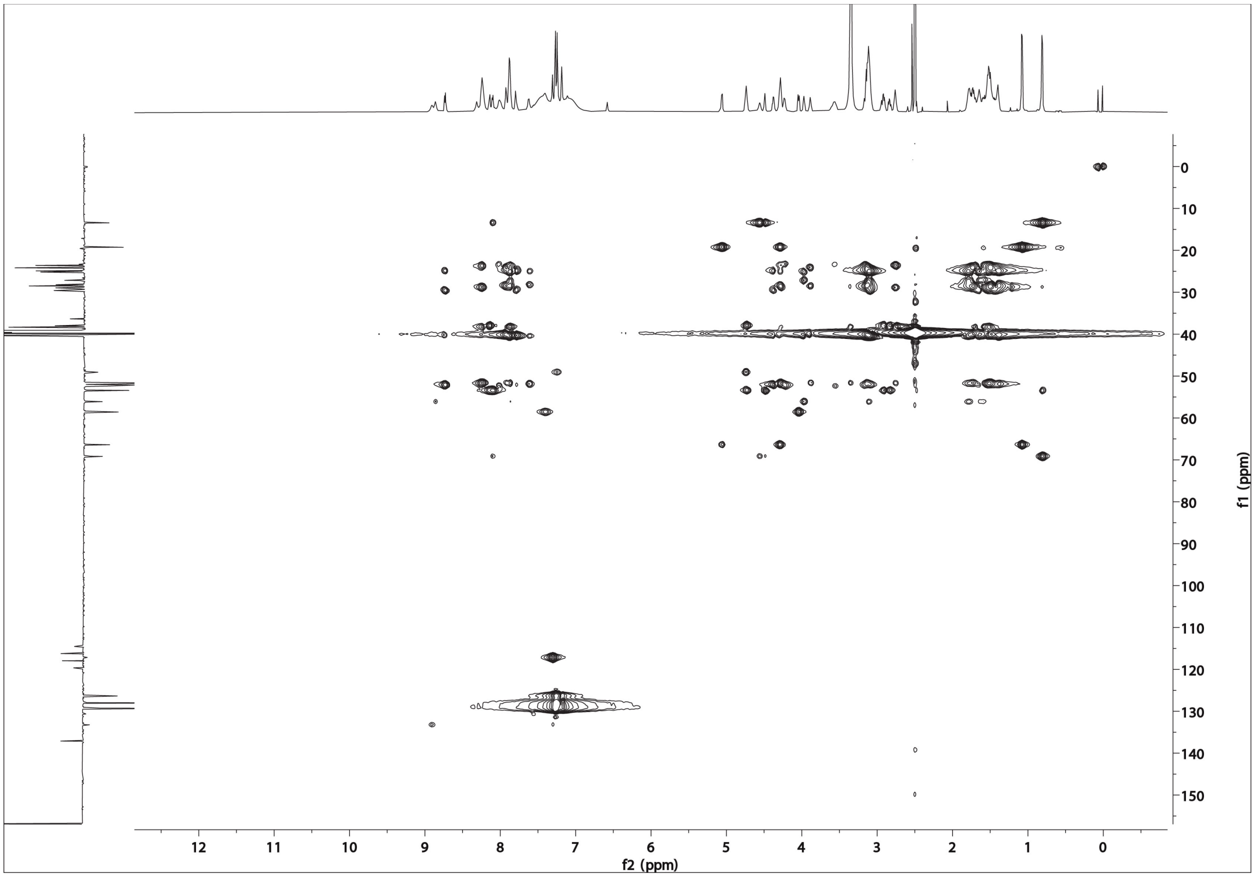

Supplementary Fig. 22 | NOESY-NMR spectrum of MKM-E

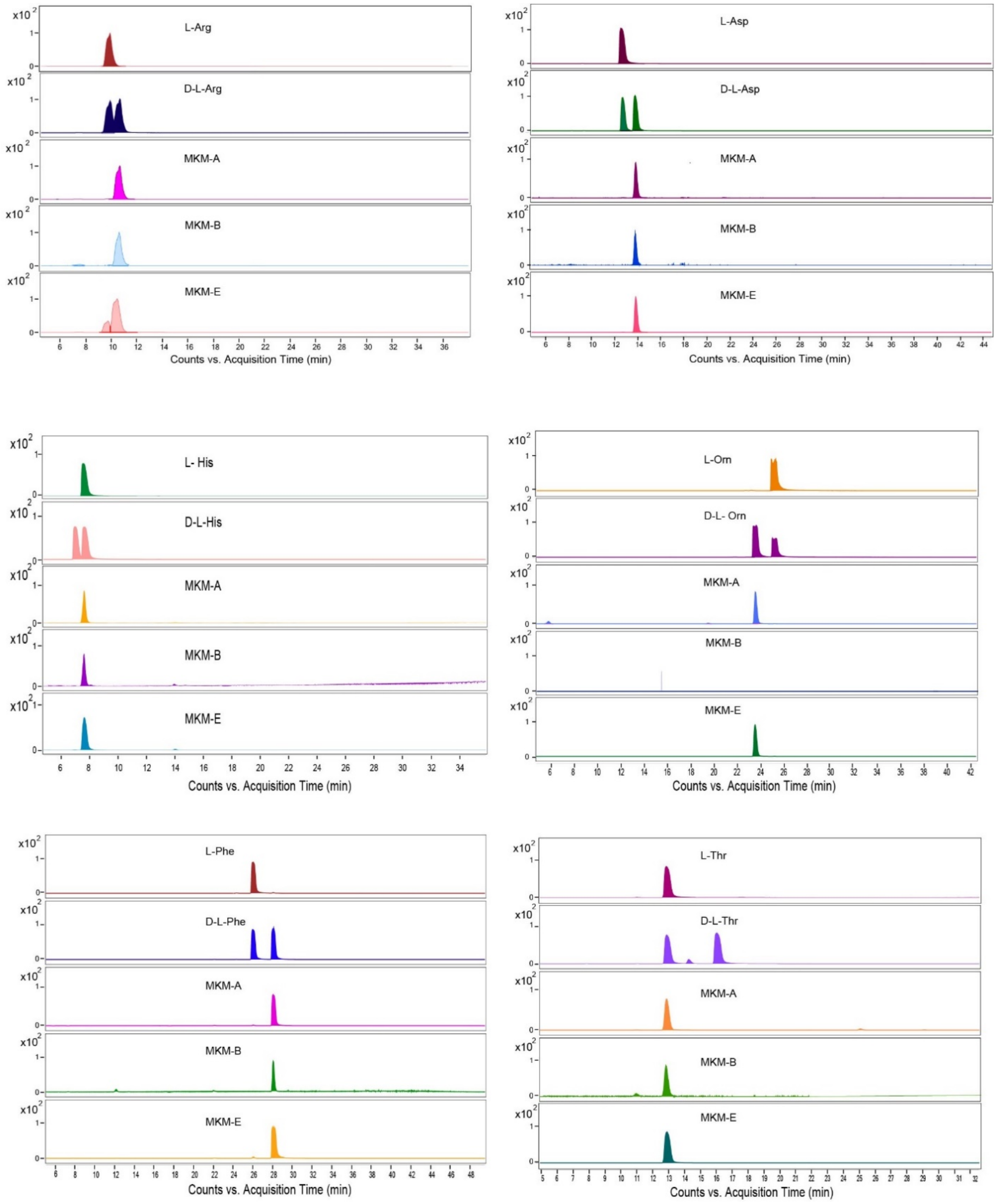

Supplementary Fig. 23 | Extracted Ion Chromatogram (EIC) of acid-hydrolyzed MKM variants treated with Marfey's reagent. Each chromatogram displays the EIC from the MKM variant samples, highlighting the peaks corresponding to the L- and D-amino acids as indicated. The retention times align with those of standard amino acids run under similar chromatographic

conditions. Because Asn converts to Asp during acid hydrolysis, the stereochemical comparison is made using Asp standards.

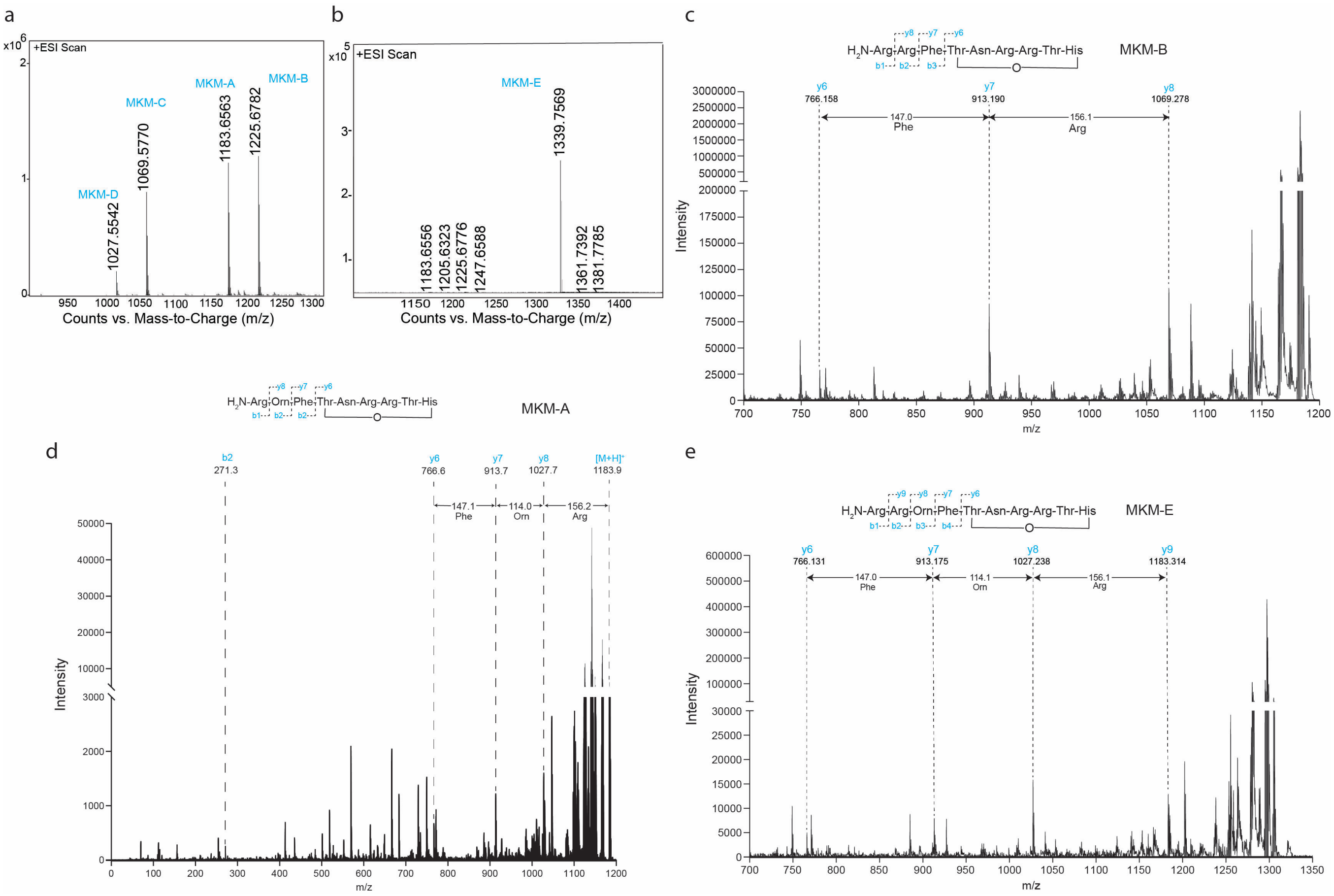

Supplementary Fig. 24 | Mass spectrometry analysis of MKM variants. a, LC-ESI-HRMS analysis of MKM-A, MKM-B, MKM-C, and MKM-D, showing the high-resolution mass spectra for each variant. **b,** LC-ESI-HRMS analysis of MKM-E. **c-e,** Tandem mass spectrometry (MALDI-MS/MS) spectrum of MKM-A, MKM-B and MKM-E. For MKM-C and MKM-D, which were available only in very low quantities, the comparative MS allowed us to propose plausible octapeptide sequences.

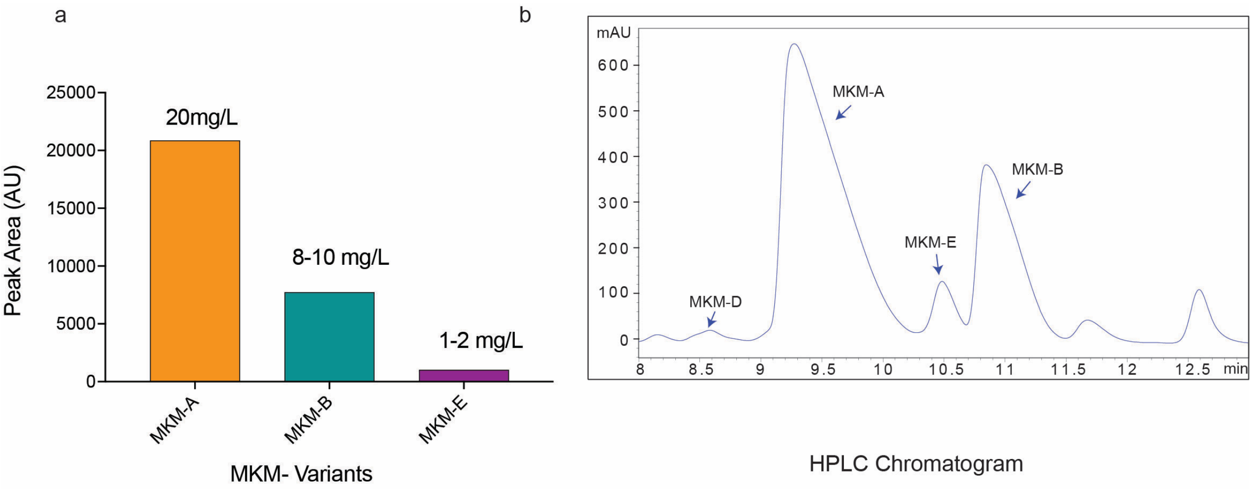

Supplementary Fig. 25 | (a) Yield proportions of the MKM variants. (b) HPLC chromatogram displaying the peaks corresponding to the individual MKM variants.

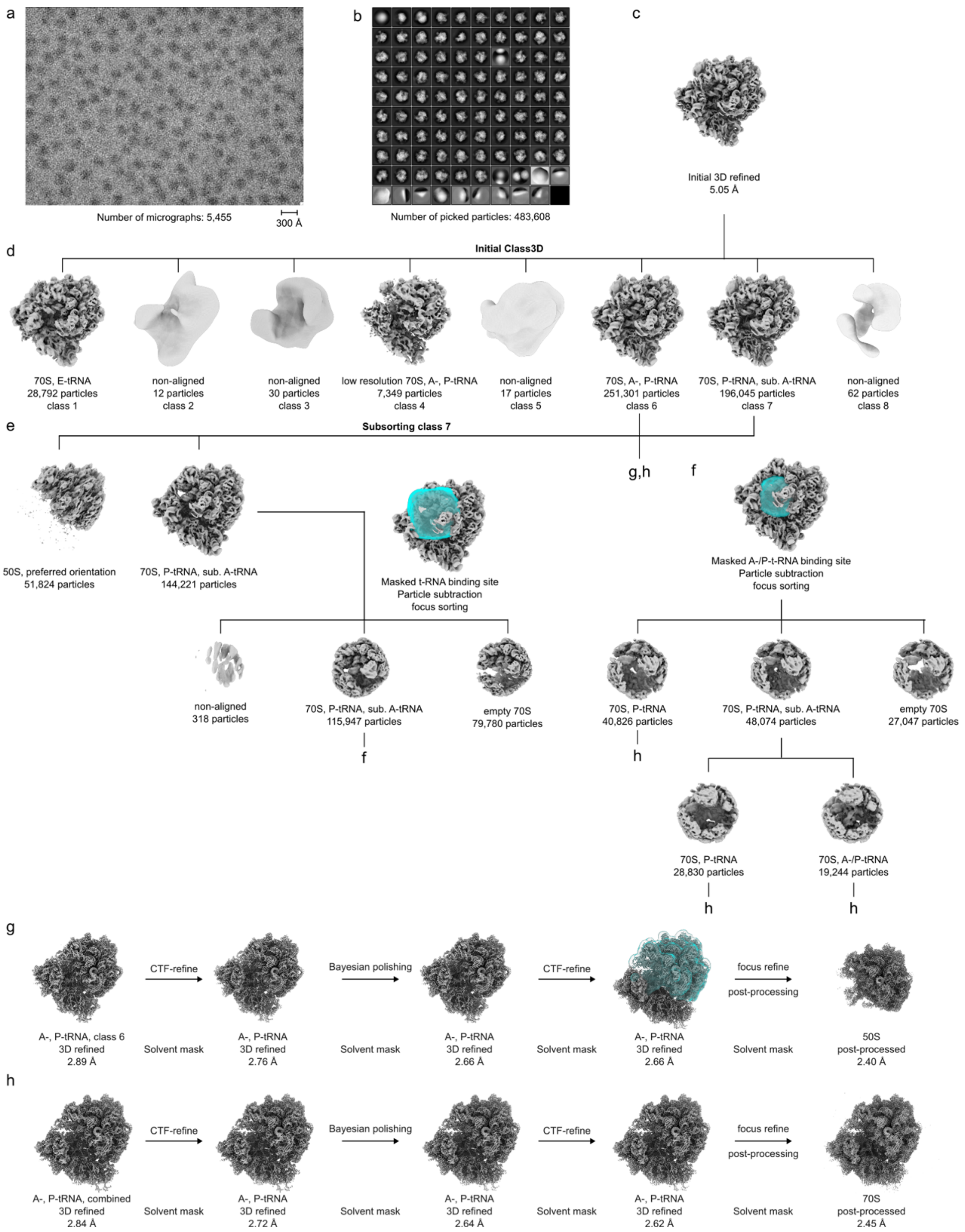

Supplementary Fig. 26. | In silico sorting scheme of the E. coli MKM complex. a-b, From (a), 5,455 micrographs (scale bar indicates 300 Å), (b) 483,608 ribosome-like particles were selected after 2D-classification. c, Particles were 3D refined at 4x decimated pixel size. d, Initial 3D classification for 200 iterations led to eight subclasses. e-f, The class 7 containing 70S with substoichiometric A-tRNA, was further subsorted (e) and the resulting 70S subclass was

subjected to 200 iterations of focused 3D classification with (f) a mask around the tRNA binding sites. g, The 70S complex containing A-tRNA and P-tRNA density was 3D refined at undecimated pixel size and subjected to CTF refinement (4th order aberrations, beam-tilt, anisotropic magnification and per-particle defocus value estimation), Bayesian polishing and again CTF refined. 70S particles were subtracted with a mask around the 50S subunit and subsequent 3D refinement resulted in a final average resolution for the masked reconstruction of 2.4 Å (at FSC0.143). h, 70S classes containing no E-tRNA were combined, refined at undecimated pixel size, subjected to CTF refinement (4th order aberrations, beam-tilt, anisotropic magnification and per-particle defocus value estimation), Bayesian polishing, again CTF refined and after a final 3D refinement yielded a final average resolution of 2.45 Å (at FSC0.143).

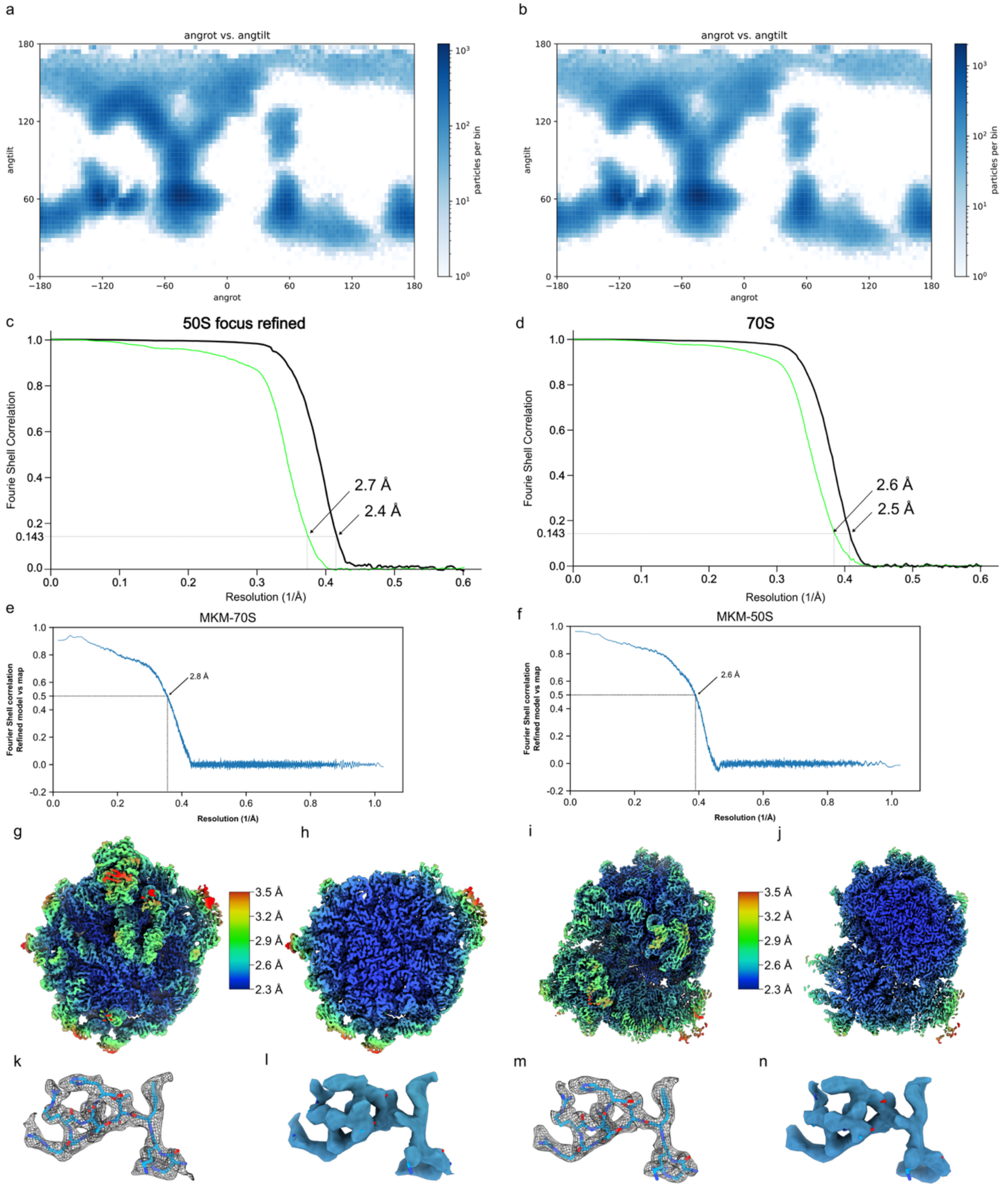

Supplementary Fig. 27. | Angular distribution and local resolution of MKM cryo-EM maps. a-b, Angular distribution of the particles that comprise the (**a**) 50S focus refined map and (**b**) the combined 70S classes. **c-d**, Fourier shell correlation (FSC) curve (green, unmasked; black, masked) of the (**c**) 50S focus refined cryo-EM map and (**d**) the 70S complex. **e-f**, FSC map versus model for (**e**) MKM-70S and (**f**) MKM-50S structures. **g-j**, Cryo-EM density map colored according to local resolution for (**g**) the 50S subunit with (**h**) transverse section and (**i**) the 70S ribosome with (**j**) transverse section. **k-n**, Cryo-EM density for MKM from (**k-l**) the 50S subunit and (**m-n**) the 70S ribosome, shown as (**k,m**) mesh with molecular model or (**l,n**) coloured according to local resolution.

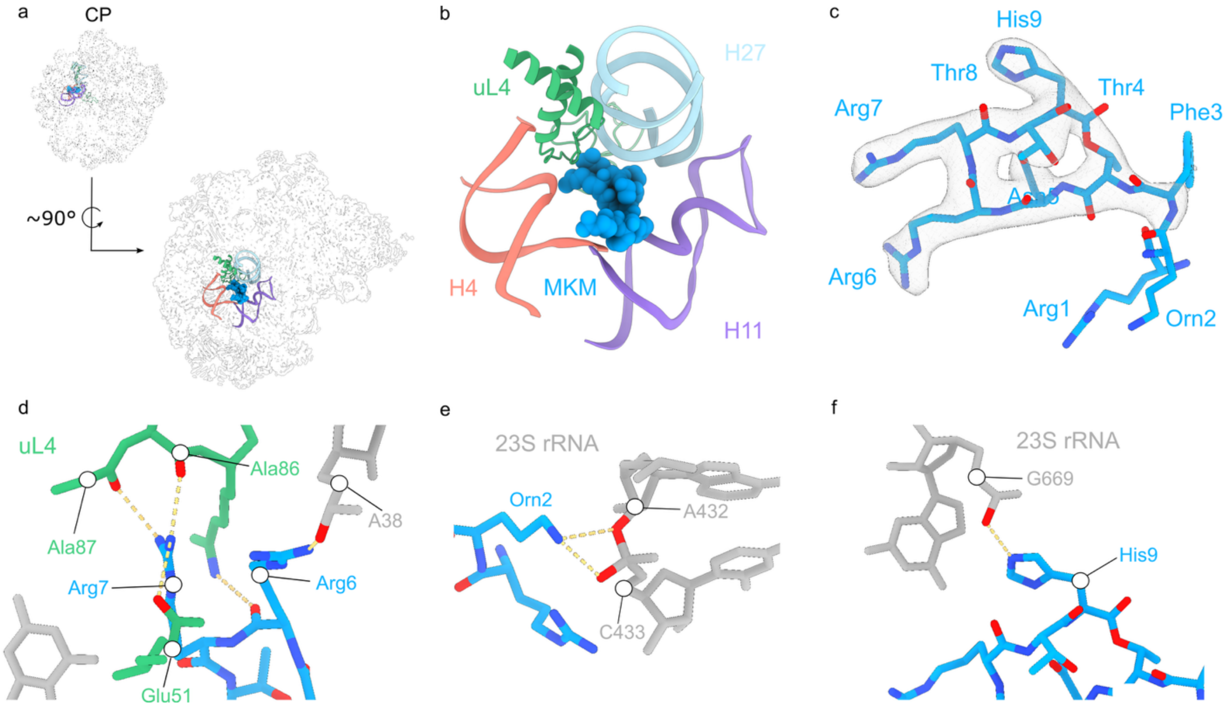

Supplementary Fig. 28. | Secondary binding site of MKM on the 50S subunit. a, Overview and (**b**) zoom onto the secondary MKM binding site on the 50S subunit, highlighting ribosomal protein uL4 (green) and 23S rRNA helices H4 (coral), H11 (purple) and H27 (light blue). **c**, Cryo-EM density (mesh) with molecular model of MKM (blue) from the secondary binding site. **d-f**, Potential hydrogen bond interactions (dashed yellow line) between MKM (blue) and (**d**) uL4, and (**e-f**) 23S rRNA nucleotides (grey).

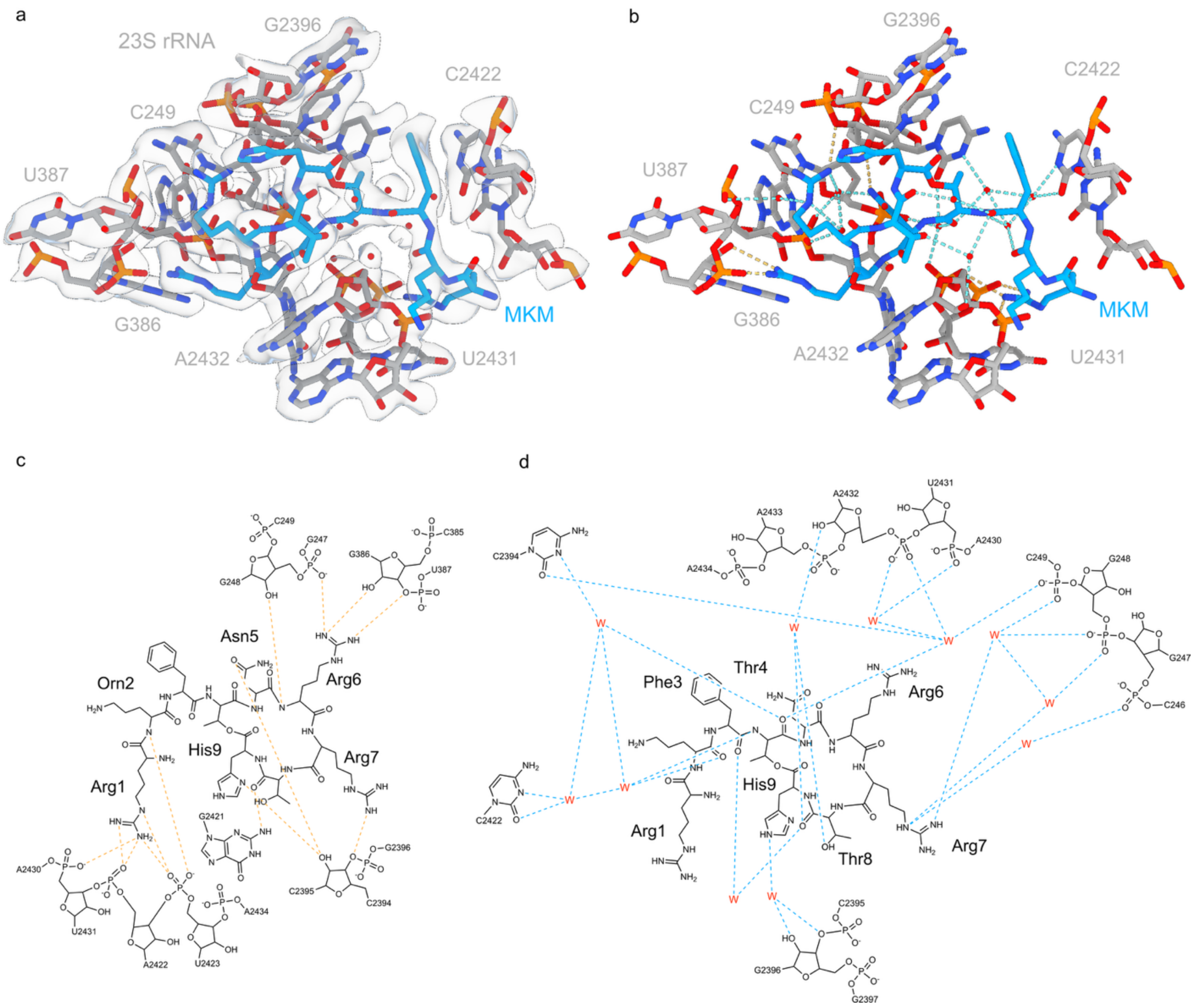

Supplementary Fig. 29. | Interaction of MKM in the E-site of the 50S subunit. a, Cryo-EM density (transparent surface) for MKM (blue), 23S rRNA nucleotides (grey) and waters (red spheres). **b**, model of MKM binding site with direct (dashed yellow lines) and water-mediated (blue dashed lines) interactions between MKM and the 23S rRNA nucleotides. **c-d**, schematic representation of the (**c**) direct (yellow) and (**d**) indirect water-mediated (blue) interactions.

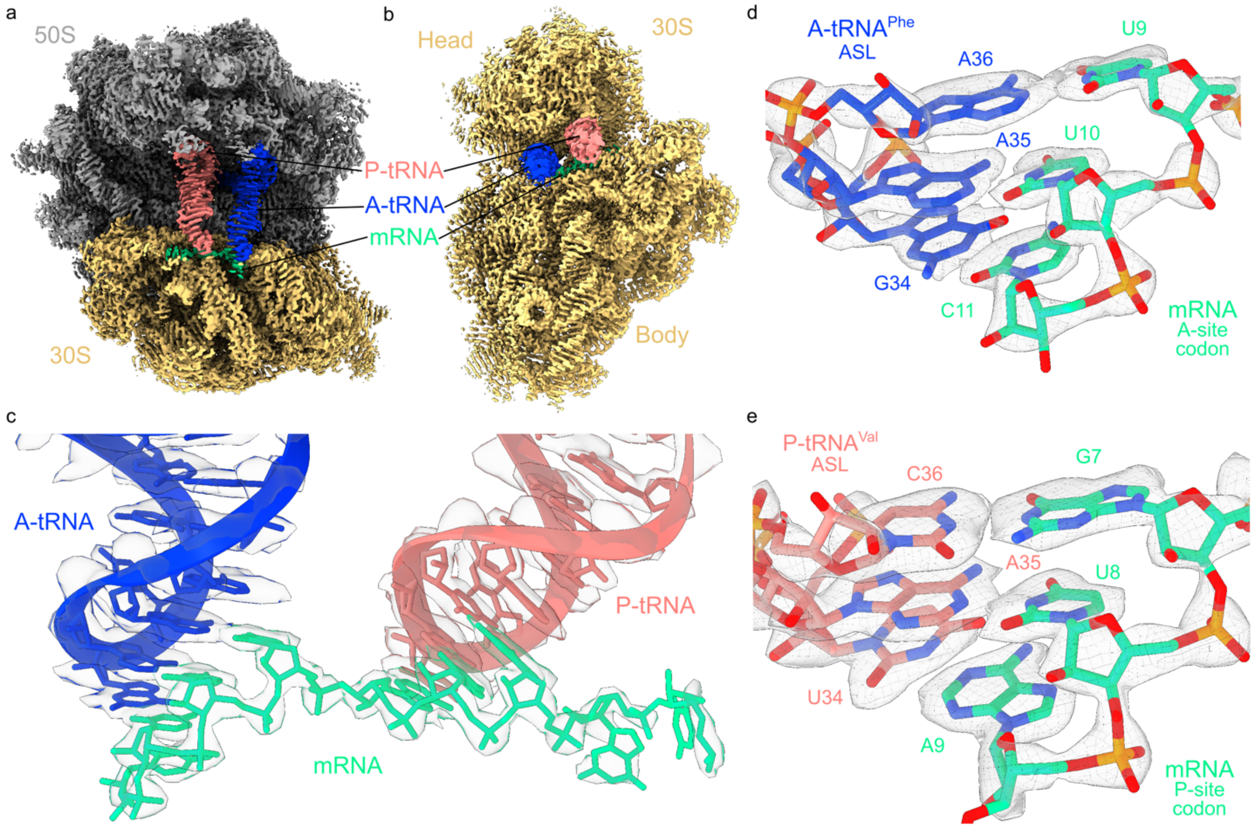

Supplementary Fig. 30 | tRNA identity and codon-anticodon interaction. a-b, cryo-EM map of the MKM-stalled 70S ribosome, shown as (a) transverse section with P-tRNA (salmon), A-tRNA (blue) and mRNA (green) highlighted and (b) 30S interface view. c, extracted cryo-EM density (grey mesh) and model of P-tRNA (salmon), A-tRNA (blue) and mRNA (green). d-e, extracted cryo-EM density of the codon-anticodon interaction for the (d) A-site tRNAPhe anticodon-stem loop (blue, GAA) and A-site codon (green, UUC) and (e) P-site tRNAVal anticodon-stem loop (salmon, UAC) and P-site codon (green, GUA).

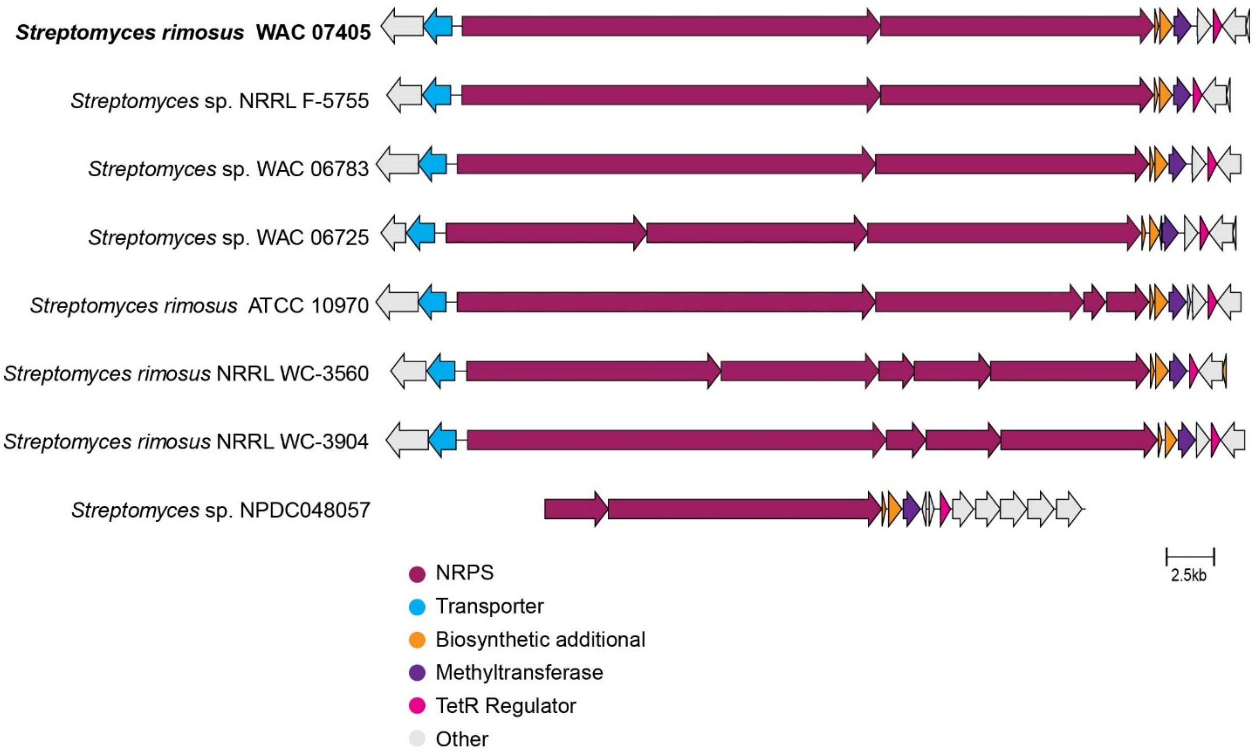

Supplementary Fig. 31 | BLAST analysis of the MKM BGC. BLAST analysis of the MKM BGC reveals its presence exclusively in the genomes of *Streptomyces rimosus*. The different genes involved in MKM biosynthesis are color-coded to distinguish them from additional associated genes, with each color representing a distinct functional group within the BGC.

Module	Stachelhaus sequence
Module 1- L-ARG	DTDDVGFVDK
Module 2- D-ARG	DSDDVGFVDK
Module 3- D- ARG/ORN	DTDDSGCVDK
Module 7- D-ARG	DTDDVGFVDK
Module 8-D-ARG	DTDDVGFVDK

Supplementary Fig. 32 | Stachelhaus code analysis. Stachelhaus code sequences for the arginine-containing modules in the MKM BGC, generated using antiSMASH.

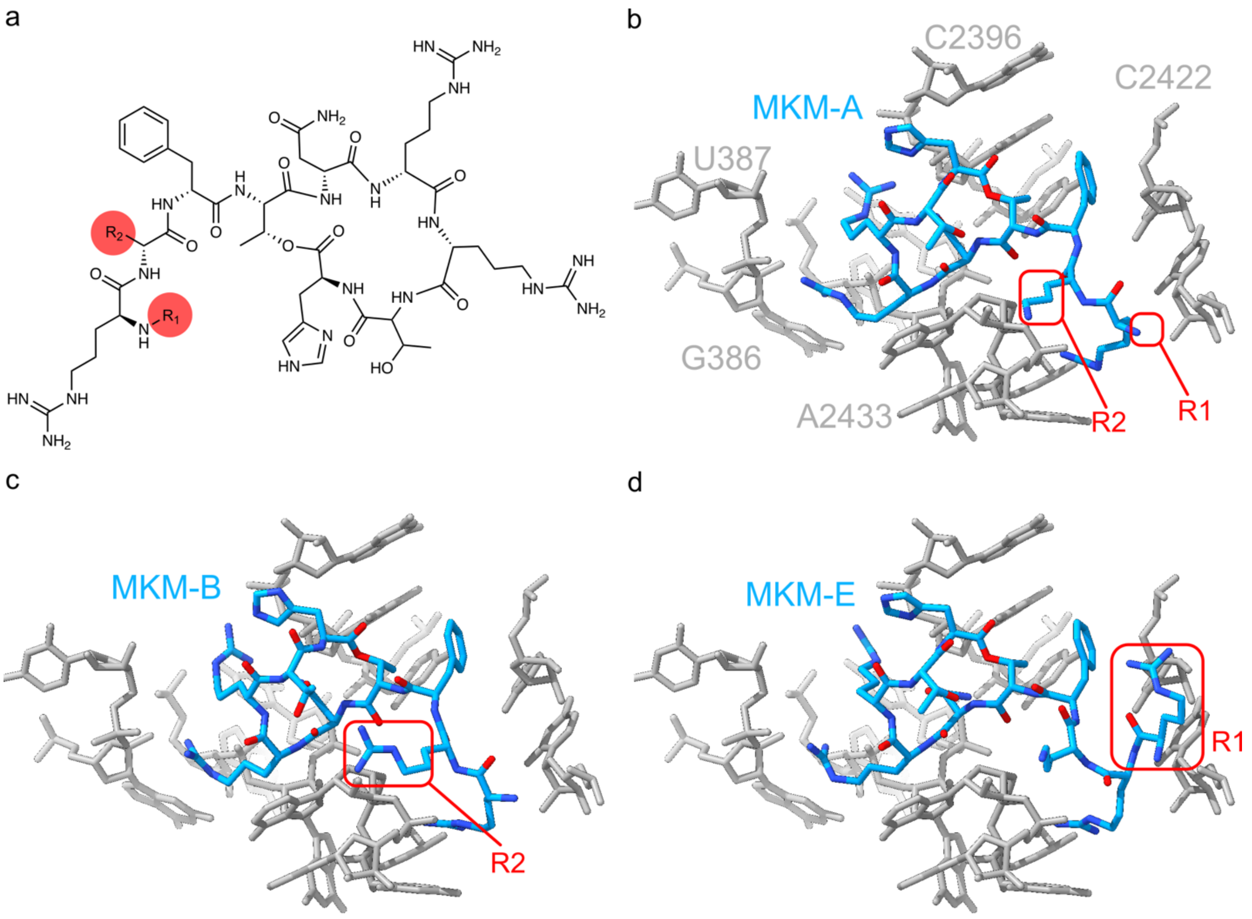

Supplementary Fig. 33 | Conserved binding mode of MKM congeners. Structural alignment shows that the binding mode determined for MKM-A is compatible with other MKM congeners, including MKM-B and MKM-E. This suggests that these variants likely share a common, context-specific mechanism of action to inhibit translocation, as elucidated for MKM-A.

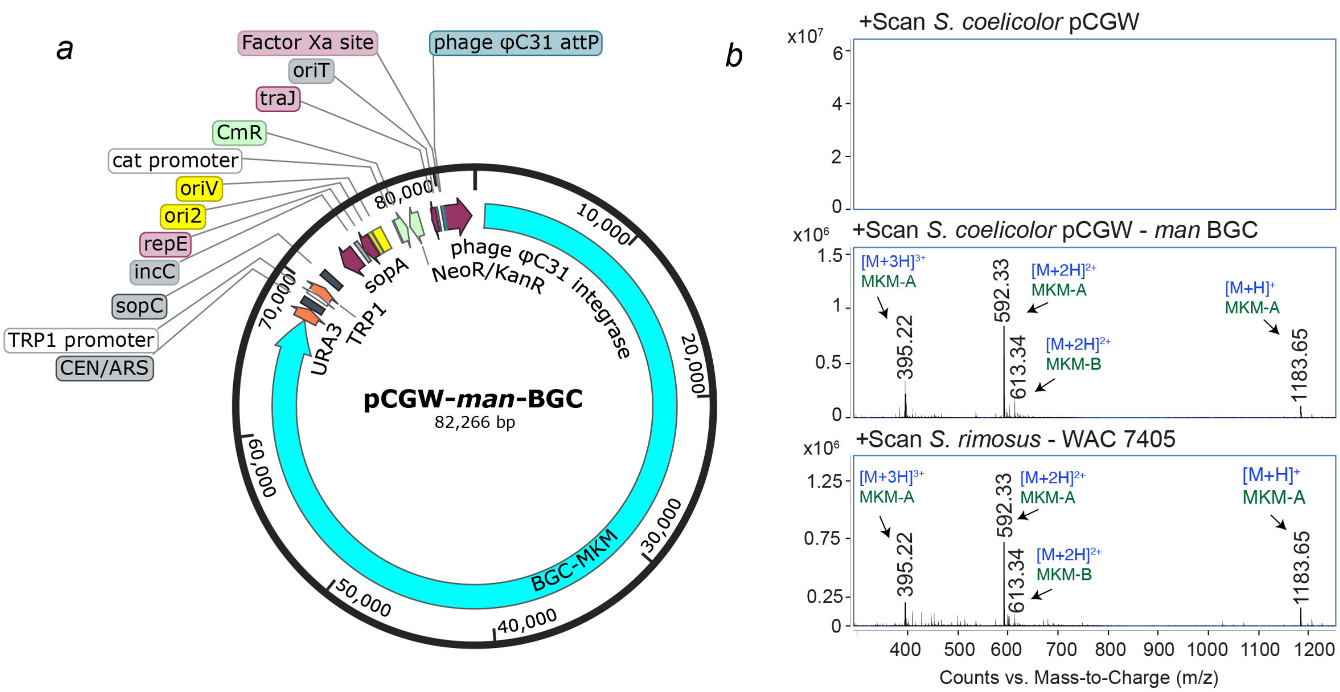

Supplementary Fig. 34 | Map of the pCGW vector with MKM BGC. (a) The pCGW plasmid served as the backbone for cloning and expression, providing essential regulatory elements. The map illustrates the successful capture and integration of the MKM BGC into the pCGW backbone, ensuring its proper organization and potential for heterologous expression. Key features of the construct, including restriction sites, selection markers, and regulatory elements, are highlighted (b) Comparison of the mass spectrometric profiles from the host strain carrying the *MKM* biosynthetic gene cluster (BGC) and the wild-type strain WAC 7405.

a

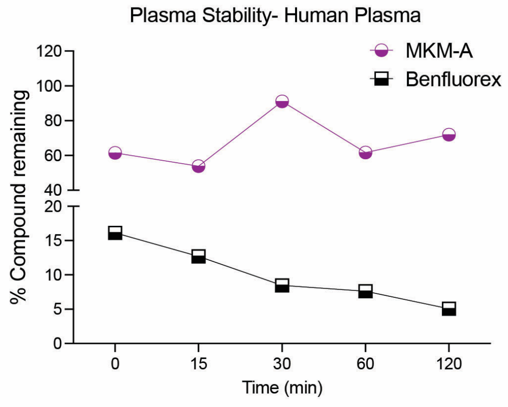

b

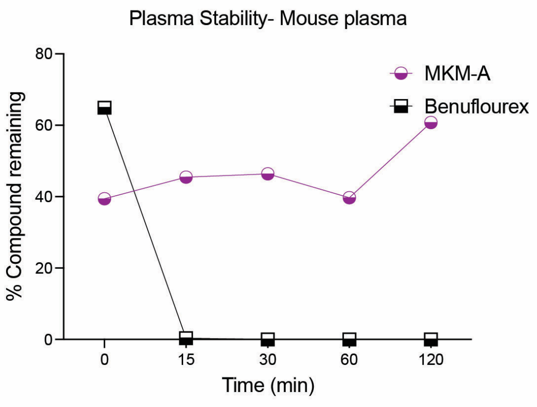

c

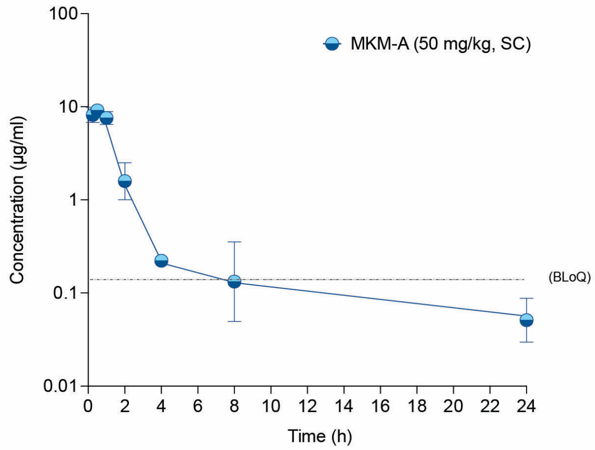

PK parameters

Parameters	Values
Cmax	9.131 µg/mL
Tmax	0.5h
Elimination rate constant (k) Lambda_Z	1.15h^{-1}
Terminal Half life (t½)	35.9 min
AUC (0 – 24h)	16.71µg.h/mL

d

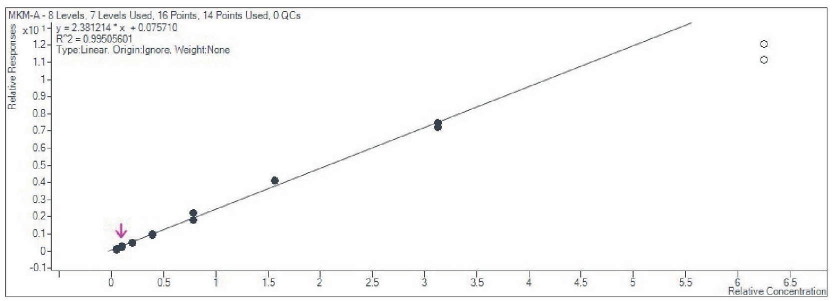

Supplementary Fig. 35| Plasma stability and pharmacokinetic characterization of MKM-A. (a, b) Comparative plasma stability profiles of MKM-A and benfluorex in human plasma (a) and mouse plasma. Data shows mean values of two replicates and is representative of two biological repeats (b), demonstrating superior stability of MKM-A over 120 minutes. (c) Plasma concentration vs time curve following subcutaneous administration of MKM-A (50 mg/kg) in mice with corresponding pharmacokinetic parameters. The dashed line indicates the approximate Below lower limit of quantification (BLoQ). Data represent mean ± SD; n = 3 per timepoint.(d) MKM-A standards for calibration curve were prepared in a matrix of plasma at the following concentration: 100, 50, 25, 12.5, 6.25, 3.12, 1.6, and 0.8 µg/ml. 100µg/ml was above the linear range used for quantitation and 0.8µg/ml was the lower limit of detection (LLOD). Calibrants contained the same amount of internal standard as the test samples (16 µg/ml). The linear range of detection was from 1.6 to 50 µg/ml.

a

MKM-A MIC (μg/ml)	*E. coli* WT	Δ*sbmA*	Δ*yejA*	Δ*yejB*	Δ*yejE*	Δ*yejF*
MHB	32	128	128-256	64	64-128	128
Minimal media	8	8	4-8	4-8	2-8	4-8

b

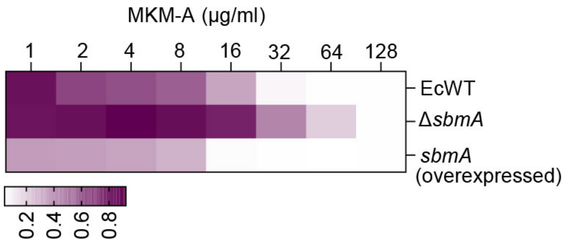

Supplementary Fig. 36 | Role of peptide uptake transporters SbmA and YejABEF in MKM susceptibility in E. coli. a, MIC of MKM for *E. coli* strains lacking SbmA, YejA, YejB, YejE, or YejF in cation-adjusted MHB medium and MOPS minimal medium. These deletion strains are part of the Keio collection[1]. **b**, Comparison of MICs upon deletion or overexpression of SbmA in wild-type *E. coli*. The overexpressed strain was a part of the ASKA library[2]. The data are displayed as a heatmap, where darker colours indicate relative growth based on OD_{600} values, as shown in the scale bar.

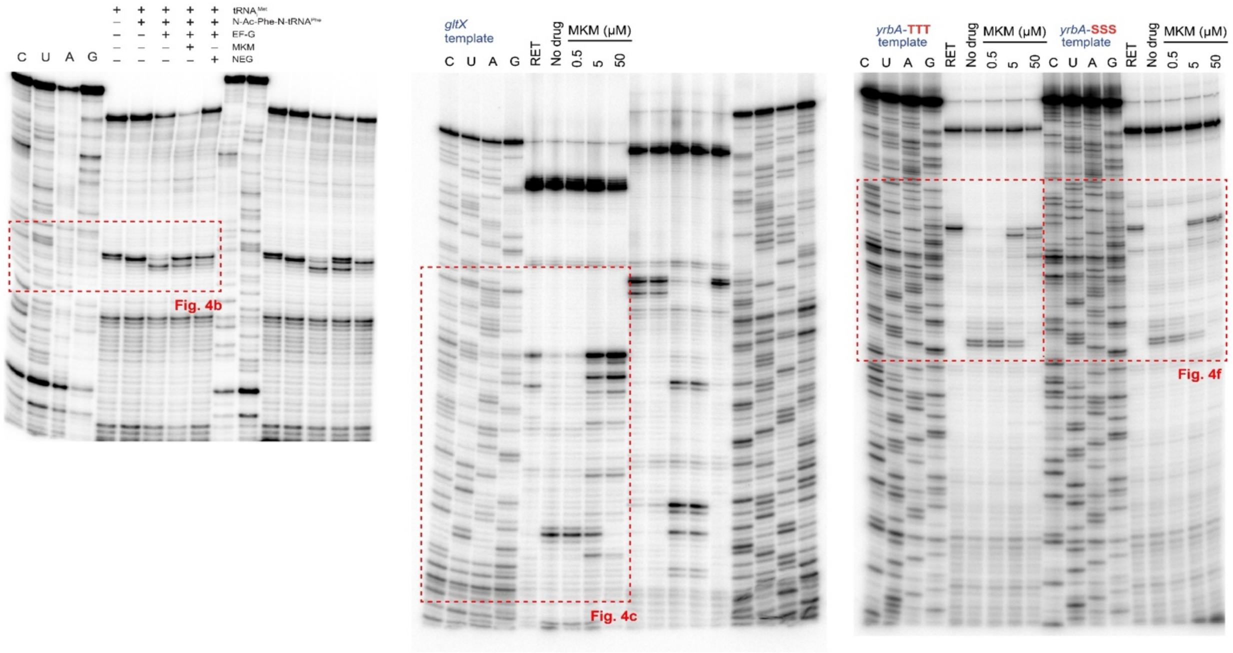

Supplementary Fig. 37 | Raw blots for toeprinting experiments used in Fig. 4b,c,f. The red box highlights the area used in main Figure panels.

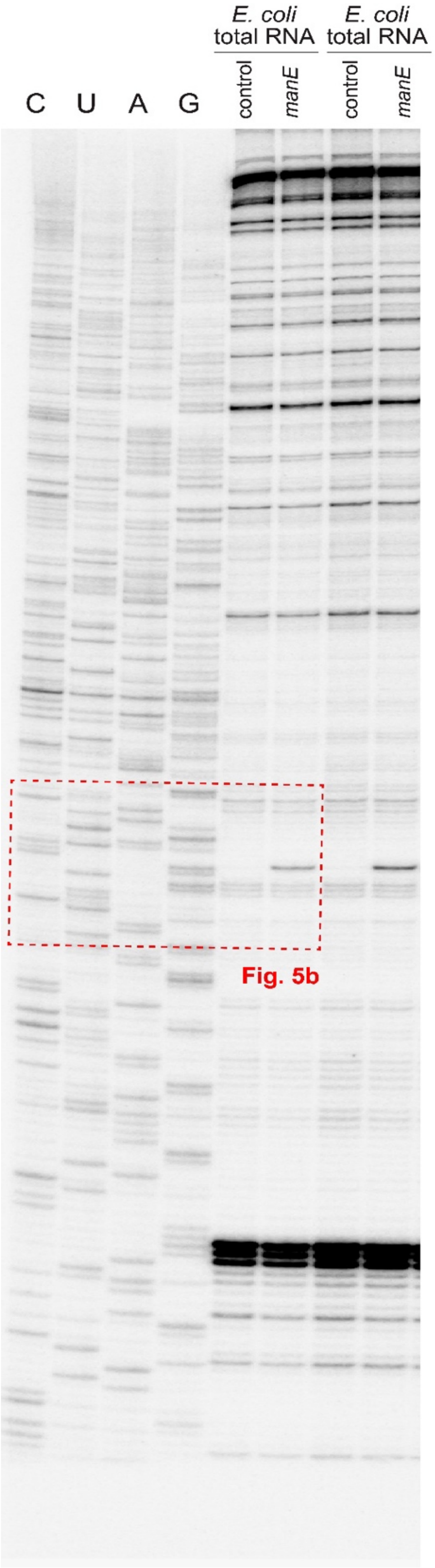

Supplementary Fig. 38 | Raw blot for toeprinting experiment used in Fig. 5b. The red box highlights the area used in main Figure panels.

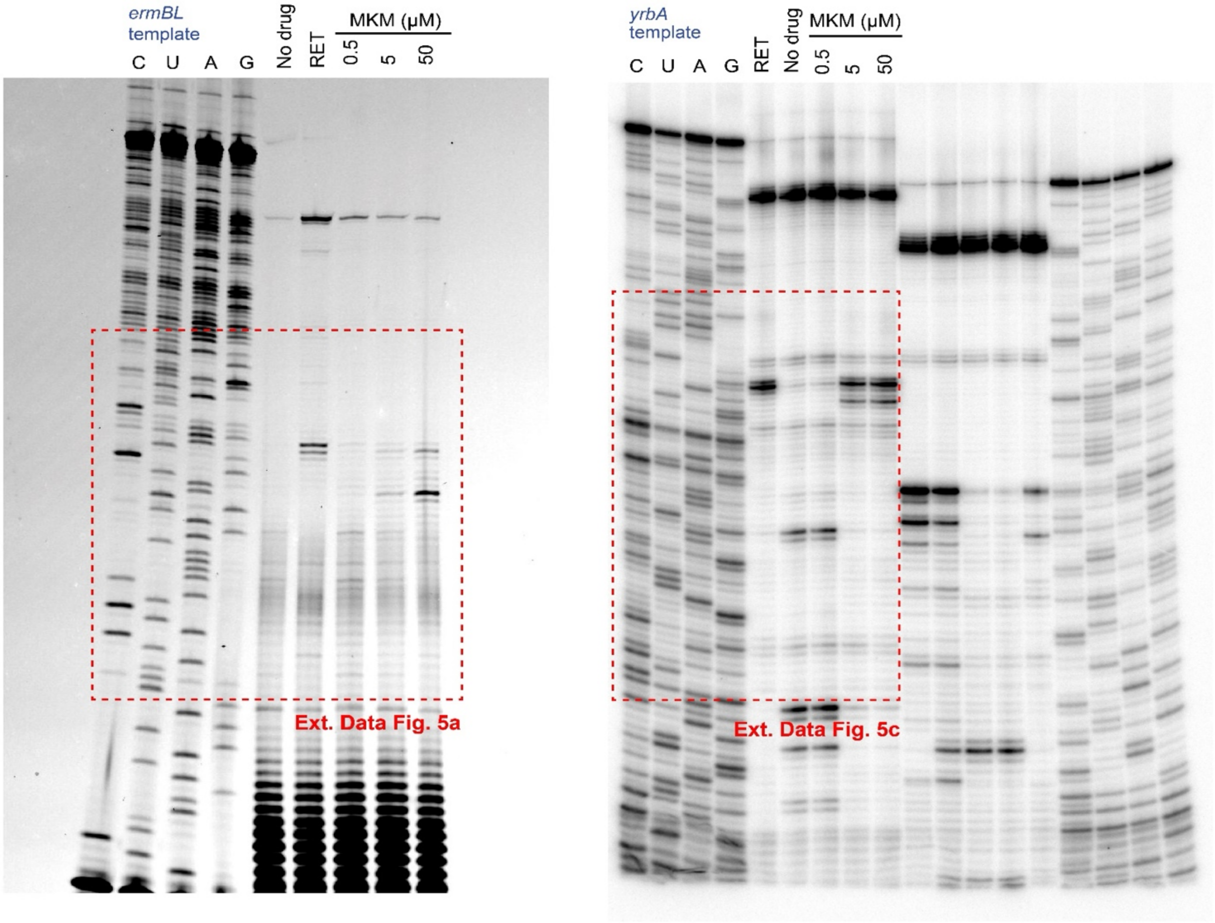

Supplementary Fig. 39 | Raw blots for toeprinting experiments used in Extended data Fig. 4 a, c. The red box highlights the area used in main Figure. panels.

SUPPLEMENTARY TABLES

Supplementary Table 1 | ^{1}H and ^{13}C NMR assignment of MKM-A

No.	δ_H	δ_C	No.	δ_H	δ_C
Arg1 CO		168.3	Arg2 CO		171.5
α	3.79 (m)	51.7	α	3.97 (m)	56.1
β	1.65 (2H, m, overlapped)	28.4	β	1.73 (m, overlapped)<br>1.80 (m, overlapped)	27.2
γ	1.47 (2H, m, overlapped)	24.0	γ	1.66 (2H, m, overlapped)	25.1
δ	3.08 (2H, m, overlapped)	40.2	δ	3.08 (2H, m, overlapped)	40.3
C=NH		156.8	C=NH		156.8
α-NH	8.21 (br s)		α-NH	8.75 (br s)	
Ornithine CO		170.6	Arg3 CO		171.4
α	4.37 (m)	51.6	α	4.28 (m, overlapped)	51.8
β	1.71 (m, overlapped)<br>1.51 (m, overlapped)	29.0	β	1.60 (m, overlapped)<br>1.46 (m, overlapped)	28.0
γ	1.60 (m, overlapped)<br>1.56 (m, overlapped)	23.4	γ	1.40 (2H, m, overlapped)	25.0
δ	2.78 (2H, m, overlapped)	38.3	δ	3.08 (2H, m, overlapped)	40.3
α-NH	8.56 (d, J = 5.0 Hz)		C=NH		156.8
NH2	7.81 (brs, overlapped)		α-NH	7.60 (d, J = 9.0 Hz)	
Phe CO		170.3	Thr2 CO		170.5
α	4.74 (m, overlapped)	53.4	α	4.07 (br d, J = 8.7 Hz)	58.6
β	2.92 (dd, J = 13.4, 6,8 Hz)<br>2.82 (dd, J = 13.4, 8,6 Hz)	38.1	β	4.30 (m, overlapped)	66.4
1’		137.1	γ	1.08 (d, J = 6.5 Hz)	19.2
2’, 6’	7.26 (t, J = 7.3 Hz)	129.2	α-NH	7.39 (br d, J = 8.7 Hz)	
3’, 5’	7.25 (t, J = 7.2 Hz)	128.0	OH	5.07 (br d, J = 8.3 Hz)	
4’	7.19 (t, J = 7.3 Hz)	126.3	His CO		168.5
α-NH	8.25 (d, J = 7.2 Hz)		α	4.11 (br dd, J = 5.5, 14.7 Hz)	52.4
Thr1 CO		166.0	β	3.54 (m, overlapped)<br>3.19 (dd, J = 5.5, 15.3 Hz)	23.4
α	4.50 (dd, J = 4.1, 7.1 Hz)	53.5	1’		129.4
β	4.57 (dq, J = 7.1, 6.2 Hz)	69.1	2’	7.30 (br s)	116.9
γ	0.82 (d, J = 6.2 Hz)	13.4	4’	8.94 (br s)	133.2
α-NH	8.15 (d, J = 4.1 Hz)		α-NH	8.04 (br s)	
Asn CO		174.6	3’-NH	8.94 (br s)	
α	4.76 (m, overlapped)	49.0			
β	3.50 (m, overlapped)<br>2.94 (m, overlapped)	36.3			
$COONH_4$		173.8			
α-NH	7.25 (m, overlapped)				

Supplementary Table 2 | ^{1}H and ^{13}C NMR assignment of MKM-B

No.	δ_H	δ_C	No.	δ_H	δ_C
Arg1 CO		168.3	<u>C</u>OONH$_4$		174.6
α	3.78 (m)	51.7	α-NH	7.25 (m, overlapped)	
β	1.65 (2H, m, overlapped)	28.4	Arg6 CO		171.5
γ	1.48 (2H, m, overlapped)	24.0	α	3.97 (m)	56.1
δ	3.08 (2H, m, overlapped)	40.1	β	1.72 (m, overlapped)<br>1.82 (m, overlapped)	27.2
<u>C</u>=NH		156.9	γ	1.65 (m, overlapped)<br>1.59 (m, overlapped)	25.2
α-NH	8.19 (m, overlapped)		δ	3.08 (2H, m, overlapped)	40.4
Arg2 CO		170.6	<u>C</u>=NH		156.9
α	4.33 (m, overlapped)	52.3	α-NH	8.75 (br s)	
β	1.67 (m, overlapped)<br>1.47 (m, overlapped)	29.4	Arg7 CO		171.5
γ	1.65 (m, overlapped)<br>1.41 (m, overlapped)	25.1	α	4.29 (m, overlapped)	51.8
δ	3.08 (2H, m, overlapped)	40.3	β	1.65 (m, overlapped)<br>1.42 (m, overlapped)	28.1
α-NH	8.55 (d, J = 7.6 Hz)		γ	1.43 (2H, m, overlapped)	25.0
			δ	3.08 (2H, m, overlapped)	40.3
Phe3 CO		170.4	<u>C</u>=NH		156.9
α	4.74 (m, overlapped)	53.4	α-NH	7.59 (d, J = 8.7 Hz)	
β	2.92 (dd, J = 13.5, 5.8 Hz)<br>2.82 (dd, J = 13.5, 9.0 Hz)	38.0	Thr8 CO		170.8
1’		137.1	α	4.08 (m, overlapped)	58.6
2’, 6’	7.28 (d, J = 7.5 Hz)	129.2	β	4.29 (m, overlapped)	66.4
3’, 5’	7.24 (t, J = 7.2 Hz)	128.0	γ	1.08 (d, J = 6.4 Hz)	19.2
4’	7.18 (t, J = 7.1 Hz)	126.4	α-NH	7.41 (overlapped)	
α-NH	8.20 (m, overlapped)		OH	5.09 (br d, J = 8.0 Hz)	
Thr4 CO		166.0	His9 CO		168.5
α	4.50 (dd, J = 4.3, 7.1 Hz)	53.5	α	4.06 (m, overlapped)	52.4
β	4.58 (dd, J = 4.3, 4.8 Hz)	69.2	β	3.54 (m, overlapped)<br>3.19 (dd, J = 4.7, 15.1 Hz)	23.2
γ	0.82 (d, J = 6.3 Hz)	13.4	1’		129.4
α-NH	8.13 (d, J = 7.1 Hz)		2’	7.29 (br s)	116.9
Asn CO		173.8	4’	8.93 (br s)	133.3
α	4.77 (m, overlapped)	49.0	α-NH	8.04 (br s)	
β	3.49 (m, overlapped)<br>2.93 (m, overlapped)	36.2			

Supplementary Table 3 | ^{1}H and ^{13}C NMR assignment of MKM-E

No.	δ_H	δ_C	No.	δ_H	δ_C
Arg1 CO		168.3	α-NH	8.10 (d, J = 7.1 Hz)	
α	3.89 (m)	51.7	Asn6 CO		173.9
β	1.73 (2H, m, overlapped)	28.5	α	4.74 (m, overlapped)	49.1
γ	1.50 (2H, m, overlapped)	24.2	β	3.58 (m, overlapped)<br>2.93 (m, overlapped)	36.3
δ	3.08 (2H, m, overlapped)	40.0	COONH4		174.6
C=NH		156.9	α-NH	7.25 (m, overlapped)	
α-NH_2	8.23 (m)		Arg7 CO		171.5
Arg2 CO		170.7	α	3.97 (m)	56.1
α	4.38 (m)	52.1	β	1.78 (2H, m, overlapped)	27.2
β	1.66 (m, overlapped)<br>1.52(m, overlapped)	29.6	γ	51.65 (2H, m, overlapped)	25.2
γ	1.43 (2H, m, overlapped)	24.9	δ	3.11 (2H, m, overlapped)	40.3
δ	3.11 (2H, m, overlapped)	40.0	C=NH		156.9
C=NH		156.9	α-NH	8.86 (br s)	
α-NH	8.73 (br s)		Arg8 CO		171.4
Ornithine3 CO		171.0	α	4.28 (m, overlapped)	51.9
α	4.27 (m)	51.7	β	1.78 (m, overlapped)<br>1.51 (m, overlapped)	28.2
β	1.73 (m, overlapped)<br>1.53 (m, overlapped)	28.9	γ	1.65 (m, overlapped)<br>1.40 (m, overlapped)	25.0
γ	1.58 (m, overlapped)<br>1.50 (m, overlapped)	23.6	δ	3.11 (2H, m, overlapped)	40.3
δ	2.76 (2H, m, overlapped)	38.3	C=NH		156.9
α-NH	8.25 (m, overlapped)		α-NH	7.62 (d, J = 8.5 Hz)	
NH2	7.81 (m, overlapped)		Thr9 CO		170.5
Phe4 CO		170.3	α	4.04 (dd, J = 8.7, 2.1 Hz)	58.6
α	4.73 (m, overlapped)	53.4	β	4.29 (m, overlapped)	66.4

β	2.92 (dd, *J* = 13.7, 4.6 Hz)<br>2.83 (dd, *J* = 13.7, 8.4 Hz)	38.0	γ	1.08 (d, *J* = 6.4 Hz)	19.3
1’		137.2	α-NH	7.41 (br s)	
2’, 6’	7.26 (d, *J* = 7.3 Hz)	129.2	OH	5.06 (br d, *J* = 8.2 Hz)	
3’, 5’	7.25 (t, *J* = 7.3 Hz)	128.0	His10 CO		168.3
4’	7.19 (t, *J* = 7.3 Hz)	126.4	α	4.23 (m)	52.4
α-NH	8.14 (d, *J* = 7.7 Hz)		β	3.57 (m, overlapped)<br>3.16 (m, overlapped)	23.3
Thr5 CO		166.0	1’		129.3
α	4.49 (dd, *J* = 3.9, 7.1 Hz)	53.5	2’	7.31 (br s)	117.1
β	4.56 (br s)	69.2	4’	8.91 (br s)	133.2
γ	0.81 (d, *J* = 6.5 Hz)	13.4	α-NH	8.01 (br s)	

Supplementary Table 4 | Calculated and Observed Masses of MKM variants.

MKMs	Chemical formula	Calculated mass $[M+H]^+$	Observed Mass $[M+H]^+$	Mass error Δ(ppm)
MKM-A	$C_{50}H_{82}N_{22}O_{12}$	1183.6561	1183.6563	0.1
MKM-B	$C_{51}H_{84}N_{24}O_{12}$	1225.6779	1225.6782	0.2
MKM-C	$C_{45}H_{72}N_{20}O_{11}$	1069.5768	1069.5770	0.1
MKM-D	$C_{44}H_{70}N_{18}O_{11}$	1027.5550	1027.5542	0.7
MKM-E	$C_{56}H_{94}N_{25}O_{13}$	1339.7572	1339.7569	0.2

Supplementary Table 5 | Proposed functions of proteins encoded by the MKM BGC (accession no: JBPBLP010000002.1) using antiSMASH v6.0.0[3] and antiSMASH v8.0.0[4]

Location	Gene	Homolog	Proposed function
ACRVTS_10640	*orf-2*	Alpha/beta hydrolase fold protein	Putative peptidase
ACRVTS_10650	*orf-1*	Penicillin amidase	Penicillin amidohydrolase
ACRVTS_10655	*manD*	ABC transporter	Transporter
ACRVTS_10660	*manA*	NRPS (modules 1-6)	Peptide synthase
ACRVTS_10665	*manB*	NRPS (modules 7-10)	Peptide synthase
ACRVTS_10670	*man1*	MbtH	MbtH-like protein
ACRVTS_10675	*manC*	Thioesterase	Thioesterase domain
ACRVTS_10680	*manE*	FtsJ-like methyltransferase	Ribosomal RNA large subunit methyltransferase
ACRVTS_10690	*man2*	(NADP oxidoreductase coenzyme F420-dependent)	Oxidoreductase
ACRVTS_10695	*manR*	Bacterial regulatory proteins, TetR family	Transcriptional regulator
ACRVTS_10700	*man3*	Amidase [*Pseudomonas putida*]	Amidase activity

Supplementary Table 6 | Mutations in MKM-resistant *E. coli* mutants from serial passaging

Mutant ID	Mutation/position	Gene(s)	Function	Gene_synonym	locus_tag
Ecmut1	+G(675/1221 nt)	*sbmA*	nonspecific peptide importer of the inner membrane	ECK0372; JW0368	BW25113_0377
Ecmut1	Δ1bp (1900/2598 nt)	*topA*	DNA topoisomerase I	ECK1268; JW1266	BW25113_1274
Ecmut1	Δ208 bp	[*rpmI*]	[*rpmI*]	ECK1715;JW1707	BW25113_1717
Ecmut2	Δ5,466 bp (IS*3*-mediated)	*yaiT*–[*yaiW*]	*yaiT*, *yaiV*, *ampH*, *sbmA*, [*yaiW*]	ECK02368; JWO362	BW25113_4580
Ecmut2	C→TA180V (GCG→GTG)	*ndh*	respiratory NADH dehydrogenase 2 / cupric reductase	ECK1095;JW1095	BW25113_1109
Ecmut2	(T)7→6(43/198 nt)	*rpmI*	50S ribosomal subunit protein bL35	ECK1715;JW1707	BW25113_1717

Supplementary Table 7 | Bacterial strains and plasmids used in the study.

Strain	Res	Experiments/ Description	Reference or source
Acinetobacter baumannii ATCC17978	-	MIC determination	Wright lab strain collection
Bacillus subtilis 168	-	MIC determination, trpC2, wild type	Common laboratory strain
Bacteroides fragilis GC73	-	MIC determination, gut isolate	Surette lab collection
Bifidobacterium breve GC96	-	MIC determination, gut isolate	Surette lab collection
Blautia obeum GC925	-	MIC determination, gut isolate	Surette lab collection
Candida albicans ATCC 90028	-	MIC determination	Wright lab strain collection
Clostridium perfringens GC1584	-	MIC determination, gut isolate	Surette lab collection
Escherichia coli BW25113	-	MIC determination and other experiments, Wild type	Common laboratory strain
E. coli BW25113 Δ*bamB*Δ*tolC*	-	MIC determination and expression of resistance genes	Wright lab strain collection
E. coli C0617	-	MIC determination, MDR	Wright clinical collection
E. coli C1507	-	MIC determination, MDR	Wright clinical collection
E. coli C1508	-	MIC determination, MDR	Wright clinical collection
E. coli ET12567	-	Conjugation experiment; *dam-13::Tn9 dcm-6 hsdMRS cat*	Wright lab strain collection
E. coli EPI300		Inducible *trfA* controlling oriV	Wright lab strain collection
E. coli ET12567 pR9406	Ap	Conjugation experiment; helper strain	Wright lab strain collection
E. coli SQ110 Δ*tolC* ::pZ*sbmA*	Ap, Sp	Selection of mutants in rRNA genes, contains a single *rrnE* operon	Mankin lab collection
E. coli BL21 Δ*tolC*		Ribosome profiling	Mankin lab collection
E. coli BW25113 Δ*sbmA*	Km	MIC determination	Keio collection
E. coli BW25113 Δ*yejA*	Km	MIC determination	Keio collection
E. coli BW25113 Δ*yejB*	Km	MIC determination	Keio collection
E. coli BW25113 Δ*yejE*	Km	MIC determination	Keio collection
E. coli BW25113 Δ*yejF*	Km	MIC determination	Keio collection
E. coli BW25113 Δ*rpmI*	Km	MIC determination	Keio collection
Klebsiella pneumoniae C1459	-	MIC determination, MDR	Wright clinical collection
K. pneumoniae C1557	-	MIC determination, MDR	Wright clinical collection
K. pneumoniae C1559	-	MIC determination, MDR	Wright clinical collection
Lactobacillus plantarum GC4	-	MIC determination, gut isolate	Surette lab collection
Mycobacterium tuberculosis Ra	-	MIC determination	Wright lab strain collection
M. smegmatis MC^2 155	-	MIC determination	Wright lab strain collection
M. avium subsp. *avium* ATCC 25291	-	MIC determination	Wright lab strain collection
M. fortuitum subsp. *fortuitum* ATCC 6841	-	MIC determination	Wright lab strain collection
M. abscessus ATCC *19977*	-	MIC determination	Wright lab strain collection
M. bovis BCG Pasteur ATCC 35734	-	MIC determination	Wright lab strain collection
Pseudomonas aeruginosa PAO1	-	MIC determination	Wright lab strain collection

Streptomyces coelicolor M1154	-	Conjugation experiment	Wright lab strain collection
S. *aureus* USA 300	-	MIC determination	Wright clinical collection
S. aureus ATCC 29213	-	MIC determination	Wright clinical collection
S. cerevisiae VL6-48N		MAT α, *his3-D200*, *trp1*-Δ*1*, *ura3*-Δ*1*, *lys2*, *ade2−101*, *met14*, *psi+cir*^{O}, TAR host strain	Wright lab strain collection
S. coelicolor M1154 -MKM-BGC	Km	Heterologous expression of MKM; contains pCGW-*man* plasmid	This study
S. coelicolor M1154 pCGW	Km	Control strain, contain vector only	This study
Streptomyces rimosus WAC 7405		MKM Producer Strain	Wright Actinomycete collection
**Plasmids**			
pCGW	Km	Engineered copy number controlled capturing vector for TAR, *aac(3)IV ura3 cen/ARS trp1 sopABC repE ori2 oriV cat neo traJ-oriT attP-int*$_{\Phi C31}$	Lab stock
pGDP3	Ap	a low copy plasmid with a P_{lac} promoter[5]	Lab stock

Res. – resistance marker, MDR – multidrug resistant, Ap – ampicillin, Km – kanamycin, Sp - spectinomycin

Supplementary Table 8 | Oligonucleotide primers used in this study.

Primer name	Sequence (5′–3′)
MKM-GBK	GCCTCCCATGGTATAAATAGTGGCAAAGCGGGCCAAGGGCCCGGCCAAGAAGACCGCCAAGAAGACGACGGCCAAGTTTAAACGAGTCCAAGATCCGTCCGGTCACGCACGCGCTGCTCAAGGTGCGGCTGGACGGCAAATGTCGAAAGCTACATATAAGGA
MKM BGC-DF:	TTTGCCTGGTGTTCTCATTC
MKMBGC-DR	AGTTCATGATGTTGACCGTG
METH-TRANS-FP	TACTAC**CAT**ATGCCCACCAGCATATTCTCCACCTC
METH-TRANS-RP	TACTAC**CTCGAG**TCAGCGCAGCCGTGC
MKM-DF	GTGAATGCCAGGAAGAGC
MKM-DR	CCAACCTGACCGGCTT
L2507	CCAGGATGTGATGAGCCG
23S_rRNA_F	CAAATTTTCGCAACACGATGATG
23S_rRNA_R	AAGGTTAAGCCTCACGG
T7_IR_AUG_F	TAATACGACTCACTATAGGGCTTAAGTATAAGGAGGAAAACATATG
yrbA_wt_F	TATAAGGAGGAAAACATATGATATACCCCTGCGGAGTGGGCGCGCG
yrbA_TTT_F	TATAAGGAGGAAAACATATGACCACGACCTGCGGAGTGGGCGCGCG
yrbA_SSS_F	TATAAGGAGGAAAACATATGAGCTCTAGCTGCGGAGTGGGCGCGCG
yrbA_wt_R	CCAACTGTCGAGGTCGGCCTAAAGCCGTTCAGTTTGCGATCGCGCGCCCACTC
yrbA_Ile_catch_R	CCAACTGTCGAGGTCGGCCTAAAGCCGTTCAATTTGCGATCGCGCGCCCACTC
posT-NV1_R	GGTTATAATGAATTTTGCTTATTAACTCGACATCGCATCAGGATTCAGCACGTGAATCCAACTGTCGAGGTCG
T7_gltX_F	TAATACGACTCACTATAGGGCATCTTTCTAAACGTAAG
gltX_NV1_R	GGTTATAATGAATTTTGCTTATTAACCCAGGAGTAAAGAGCTTAAGTACGCGCGCCGCC
NV1	GGTTATAATGAATTTTGCTTATTAA
MF_F1	ATTAATACGACTCACTATAGGGCAACCTAAAACTTACACACGCCCCGGTAAGGAAATAAAAAT
MF_F2	GCCCCGGTAAGGAAATAAAAATGTTCAAAGCATTCAAAAACATCATACGTACTCGTACTC
MF_R	GGTTATAATGAATTTTGCTTATTAACCTTGCCTGCGCTTAAAGAGTACGAGTACGTATGATGT
T7_ermBL_F	TAATACGACTCACTATAGGGAGACTTAAGTATAAGGAGGAAAAAATATGTTGGTATTCCAAATGCGTAATGTAGATAA
ermBL_UGA_R	GGTTATAATGAATTTTGCTTATTAACGATAGAATTCTATCACTCAAATAGTAGATGTTTTATCTACATTACGCATTT

Supplementary Table 9 | mRNA templates used in the study.

Template name	Sequence (5′–3′)**	ORF translation
yrbA (wt)	**AAUACGACUCACUAUAGGG**CUUAAGUAUAAGGAGGA<br>AAACAU**AUGAUAUACCCCUGCGGAGUGGGCGCGCGA**<br>**UCGCAAACUGAACGGCUUUAG**GCCGACCUCGACAGU<br>UGGAUUCACGUGCUGAAUCCUGAUGCGAUGUCGA**GU**<br>**UAAUAAGCAAAAUUCAUUAUAACC**	**MIYPCGVGARSQ<br>TERL***
*yrbA*_SSS	**UAAUACGACUCACUAUAGGG**CUUAAGUAUAAGGAGG<br>AAAACAU**AUGAGCUCUAGCUGCGGAGUGGGCGCGCG**<br>**AUCGCAAAUUGAACGGCUUUAG**GCCGACCUCGACAG<br>UUGGAUUCACGUGCUGAAUCCUGAUGCGAUGUCGA**G**<br>**UUAAUAAGCAAAAUUCAUUAUAACC**	**MSSSCGVGARSQ<br>IERL***
*yrbA*_TTT	**UAAUACGACUCACUAUAGGG**CUUAAGUAUAAGGAGG<br>AAAACAU**AUGACCACGACCUGCGGAGUGGGCGCGCG**<br>**AUCGCAAAUUGAACGGCUUUAG**GCCGACCUCGACAG<br>UUGGAUUCACGUGCUGAAUCCUGAUGCGAUGUCGA**G**<br>**UUAAUAAGCAAAAUUCAUUAUAACC**	**MTTTCGVGARSQ<br>IERL***
gltX	**UAAUACGACUCACUAUAGGG**CAUCUUUCUAAACGUA<br>AGGCCAUUUC**AUGAAAAUCAAAACUCGCUUCGCGCC**<br>**AAGCCCAACAGGCUAUCUGCACGUUGGCGGCGCGCG**<br>**UACUUAA**GCUCUUUACUCCUGG**GUUAAUAAGCAAAA**<br>**UUCAUUAUAACC**	**MKIKTRFAPSPT<br>GYLHVGGART***
ermBL	**UAAUACGACUCACUAUAGGG**AGACUUAAGUAUAAGG<br>AGGAAAAAAU**AUGUUGGUAUUCCAAAUGCGUAAUGU**<br>**AGAUAAAACAUCUACUAUUUGA**GUGAUAGAAUUCUA<br>UC**GUUAAUAAGCAAAAUUCAUUAUAACC**	**MLVFQMRNVDKT<br>STI***
MFK	AUU**AAUACGACUCACUAUAGGG**CAACCUAAAACUUA<br>CACACGCCCCGGUAAGGAAAUAAAA**AUGUUCAAAGC**<br>**AUUCAAAAACAUCAUACGUACUCGUACUCUUUAA**GC<br>GCAGGCAAG**GUUAAUAAGCAAAAUUCAUUAUAACC**	**MFKAFKNIIRTR<br>TL***

** ORF in the template sequence is shown in red, T7 promotor is shown in blue, NV1 primer binding sequence – in green.

Supplementary data references

1 Baba, T. *et al.* Construction of Escherichia coli K-12 in-frame, single-gene knockout mutants: the Keio collection. *Mol Syst Biol* **2**, 2006 0008 (2006). https://doi.org/10.1038/msb4100050

2 Kitagawa, M. *et al.* Complete set of ORF clones of Escherichia coli ASKA library (a complete set of E. coli K-12 ORF archive): unique resources for biological research. *DNA Res* **12**, 291-299 (2005). https://doi.org/10.1093/dnares/dsi012

3 Blin, K. *et al.* antiSMASH 6.0: improving cluster detection and comparison capabilities. *Nucleic Acids Res* **49**, W29-W35 (2021). https://doi.org/10.1093/nar/gkab335

4 Blin, K. *et al.* antiSMASH 8.0: extended gene cluster detection capabilities and analyses of chemistry, enzymology, and regulation. *Nucleic Acids Res* **53**, W32-W38 (2025). https://doi.org/10.1093/nar/gkaf334

5 Cox, G. *et al.* A Common Platform for Antibiotic Dereplication and Adjuvant Discovery. *Cell Chem Biol* **24**, 98-109 (2017). https://doi.org/10.1016/j.chembiol.2016.11.011
