# Supplementary material for: A natural depsipeptide antibiotic binds the E-site of the bacterial ribosome
Source: Nature. 2026 Jun 3;655(8123):737–46. doi: 10.1038/s41586-026-10589-2 (PMC13372681; doi:10.1038/s41586-026-10589-2)
Supplement: Supplementary file 2 — Reporting Summary [file 41586_2026_10589_MOESM2_ESM.pdf]

nature portfolio

Corresponding author(s): Dr. Gerard D. Wright

Last updated by author(s): Apr 9, 2026

Reporting Summary

Nature Portfolio wishes to improve the reproducibility of the work that we publish. This form provides structure for consistency and transparency in reporting. For further information on Nature Portfolio policies, see our Editorial Policies and the Editorial Policy Checklist.

Statistics

For all statistical analyses, confirm that the following items are present in the figure legend, table legend, main text, or Methods section.

n/a	Confirmed	
☐	☒	The exact sample size (n) for each experimental group/condition, given as a discrete number and unit of measurement
☐	☒	A statement on whether measurements were taken from distinct samples or whether the same sample was measured repeatedly
☐	☒	The statistical test(s) used AND whether they are one- or two-sided<br>*Only common tests should be described solely by name; describe more complex techniques in the Methods section.*
☒	☐	A description of all covariates tested
☐	☒	A description of any assumptions or corrections, such as tests of normality and adjustment for multiple comparisons
☐	☒	A full description of the statistical parameters including central tendency (e.g. means) or other basic estimates (e.g. regression coefficient) AND variation (e.g. standard deviation) or associated estimates of uncertainty (e.g. confidence intervals)
☐	☒	For null hypothesis testing, the test statistic (e.g. F, t, r) with confidence intervals, effect sizes, degrees of freedom and P value noted<br>*Give P values as exact values whenever suitable.*
☒	☐	For Bayesian analysis, information on the choice of priors and Markov chain Monte Carlo settings
☒	☐	For hierarchical and complex designs, identification of the appropriate level for tests and full reporting of outcomes
☒	☐	Estimates of effect sizes (e.g. Cohen's d, Pearson's r), indicating how they were calculated

Our web collection on statistics for biologists contains articles on many of the points above.

Software and code

Policy information about availability of computer code

Data collection	CryoEM data were collected using the EPU 2.6.1 software (FEI, Netherlands)
Data analysis	Breseq v0.37.1<br>GraphPad Prism 10.2.3. and v. 10.5.0.<br>antiSMASH v.6.0, 8.0<br>MassHunter Quantitation (v10.1)<br>Phoenix WinNonlin (Build 8.4.)<br>MEGA11 with the in-built MUSCLE algorithm<br>RELION v5.0.0 with MotionCor2 v1.2.1, CTFFIND 4.1.14, and crYOLO v1.8.04b47 were used for processing micrographs, picking particles, classification and refining cryo-EM maps. RELION was used to calculate local resolution. Coot v0.9.8.92 and aceDRG (CCP4 v8.0.017) for model building and Refmac 5 in Servalcat v0.4.28 and Phenix (1.20.1-4487) for model refinement and statistics. Figures were generated using ChimeraX v1.6.1.<br>Pipeline used for ribosome profiling data processing is available at: https://github.com/mmaiensc/RiboSeq/wiki/RiboSeq-profiling

For manuscripts utilizing custom algorithms or software that are central to the research but not yet described in published literature, software must be made available to editors and reviewers. We strongly encourage code deposition in a community repository (e.g. GitHub). See the Nature Portfolio guidelines for submitting code & software for further information.

Data

Policy information about availability of data

All manuscripts must include a data availability statement. This statement should provide the following information, where applicable:

- Accession codes, unique identifiers, or web links for publicly available datasets
- A description of any restrictions on data availability
- For clinical datasets or third party data, please ensure that the statement adheres to our policy

Cryo-EM maps and molecular models were deposited in the Electron Microscopy Data Bank (EMDB) and Protein Data Bank (PDB) with accession codes EMD-54009 and PDB ID 9RJA (MKM-70S complex) and EMD-53943 and PDB ID 9RFW (MKM-50S complex), respectively.
All previously published structures used in this work for structural comparisons were retrieved from the RCSB Protein Data Bank: PDB entries 2OTJ, 4U3U, 4U4R, 4U4Z, 5O61, 6SGC, 6SPG, 6ND6,7K00, 8AKN, 8GLP, 8P2G,.
This Whole Genome Shotgun project (Streptomyces rimosus WAC 7405) has been deposited at DDBJ/ENA/GenBank under the accession JBPBLP000000000. The version described in this paper is version JBPBLP010000000. Sequencing data collected for ribosome profiling experiment were deposited in NCBI Sequence Read Archive (SRA) with BioProject ID PRJNA1265262.

Research involving human participants, their data, or biological material

Policy information about studies with human participants or human data. See also policy information about sex, gender (identity/presentation), and sexual orientation and race, ethnicity and racism.

Reporting on sex and gender	The study used commercially available human blood (HUMANWBK2-0000405, Gender Unspecified) purchased from BioIVT (New York, USA).
Reporting on race, ethnicity, or other socially relevant groupings	NA
Population characteristics	NA
Recruitment	NA
Ethics oversight	Not applicable since we used commercially available human blood.

Note that full information on the approval of the study protocol must also be provided in the manuscript.

Field-specific reporting

Please select the one below that is the best fit for your research. If you are not sure, read the appropriate sections before making your selection.

☒ Life sciences ☐ Behavioural & social sciences ☐ Ecological, evolutionary & environmental sciences

For a reference copy of the document with all sections, see nature.com/documents/nr-reporting-summary-flat.pdf

Life sciences study design

All studies must disclose on these points even when the disclosure is negative.

Sample size	No sample size-calculation was performed. For the mouse PK study, the sample size was chosen to demonstrate replication of results while considering ethical constraints. For other experiments, sample size was chosen to show the statistical significance and replication of results while considering material availability.
Data exclusions	Micrographs with low estimated resolution or poorly fitted CTFs were discarded, as were particles that clustered into poorly defined classes during 2D and 3D classification, otherwise no data was excluded.
Replication	All the experiments were performed with three biological replicates, and the representative results from two independent experiments with similar results are shown, as mentioned in figure legends. All experiments were successfully reproduced.
Randomization	For 3D refinement in RELION, particles are randomly placed in one of two subsets. These subsets are maintained for CTF refinement. For other experiments too, samples were randomly allocated.
Blinding	Blinding was not deemed necessary as the experiments conducted did not involve the experimenter assessing or comparing distinct test groups

Reporting for specific materials, systems and methods

We require information from authors about some types of materials, experimental systems and methods used in many studies. Here, indicate whether each material, system or method listed is relevant to your study. If you are not sure if a list item applies to your research, read the appropriate section before selecting a response.

Materials & experimental systems

n/a	Involved in the study
☒	☐ Antibodies
☐	☒ Eukaryotic cell lines
☒	☐ Palaeontology and archaeology
☐	☒ Animals and other organisms
☒	☐ Clinical data
☒	☐ Dual use research of concern
☒	☐ Plants

Methods

n/a	Involved in the study
☒	☐ ChIP-seq
☒	☐ Flow cytometry
☒	☐ MRI-based neuroimaging

Eukaryotic cell lines

Policy information about cell lines and Sex and Gender in Research

Cell line source(s)	HEK293 (ATCC CRL-1573; generation 6), HepG2 cells (ATCC HB8065; generation 18)
Authentication	The cell line was not authenticated.
Mycoplasma contamination	The cell lines used were not tested for mycoplasma contamination.
Commonly misidentified lines (See ICLAC register)	No commonly misidentified cell lines were used.

Animals and other research organisms

Policy information about studies involving animals; ARRIVE guidelines recommended for reporting animal research, and Sex and Gender in Research

Laboratory animals	All animal studies were performed with uninfected female ICR CD-1 mice (seven–ten weeks old; Envigo)
Wild animals	The study did not involve any wild animals.
Reporting on sex	Only females mice were used in the experiments.
Field-collected samples	No field samples were used.
Ethics oversight	All animal studies were conducted according to guidelines set by the Canadian Council on Animal Care using protocols approved by the Animal Review Ethics Board at McMaster University under Animal Use Protocol #24-37.

Note that full information on the approval of the study protocol must also be provided in the manuscript.

Plants

Seed stocks	NA
Novel plant genotypes	NA
Authentication	NA
