# Supplementary material for: A natural depsipeptide antibiotic binds the E-site of the bacterial ribosome
Source: Nature. 2026 Jun 3;655(8123):737–46. doi: 10.1038/s41586-026-10589-2 (PMC13372681; doi:10.1038/s41586-026-10589-2)
Supplement: Supplementary file 4 — Peer Review file [file 41586_2026_10589_MOESM4_ESM.pdf]

nature portfolio

Peer Review File

A Natural Depsipeptide Antibiotic binds the E site of the Bacterial Ribosome

Corresponding Author: Professor Gerard Wright

Any redactions in this file are there to maintain patient confidentiality, the confidentiality of unpublished data, or to remove third-party material.

This file contains all reviewer reports in order by version, followed by all author rebuttals in order by version.

Version 0:

Reviewer comments:

Referee #1

(Remarks to the Author)
The authors report the discovery of a new antimicrobial depsipeptide, manikomycin (MKM), from Streptomyces rimosus, with selective activity against Enterobacteriaceae. Resistance mapping identified the inner membrane transporter SbmA as essential for compound uptake, explaining some selectivity in its action. The resistance studies also implicated the ribosome as the target. The highlight of the study is the structural analysis performed on MKM's interaction with stalled ribosomes using toeprinting and cryo-EM, which shows that MKM binds the large ribosomal subunit, blocking the movement of deacylated tRNA into the E site. This is consistent with resistance mutations and in vitro translation assays, which confirmed the mechanism of action. Self-resistance in the Streptomyces producer was also explained by identifying ManE, an rRNA methyltransferase, which prevents MKM's binding to the ribosome by a posttranscriptional modification.

The MKM's mechanism of action is novel for bacteria, and in this regard, it is an interesting study. The compound itself however is not a realistic drug candidate. Potency is moderate, and MIC tests were not performed in the presence of serum, so it is not clear how much of the activity will be diminished by protein binding; frequency of resistance due to null mutations in transport is high, 10-7; spectrum of action limited to E. coli and Klebsiella; the compound is a polycation, likely to cause nephrotoxicity; stability in vivo is poor, precluding mammalian study.

Referee #2

(Remarks to the Author)
I co-reviewed this manuscript with one of the reviewers who provided the listed reports.

Referee #3

(Remarks to the Author)
Kaur et al. report the identification and characterization of manikomycin (MKM), a novel antibiotic that inhibits bacterial mRNA-tRNA translocation through specific targeting of the ribosomal P site. MKM is active against multi-drug-resistant Enterobacteriaceae, displays low toxicity against human cells, and is not affected by known resistance mechanisms, making it an excellent candidate for future drug development. Remarkably, MKM was identified from a known antibiotic producer, Streptomyces rimosus, thanks to an improved fractionation approach. The importance of this work therefore extends beyond the discovery of a new, promising antibiotic by showing that careful analysis of previously analyzed soil organisms can still yield novel, therapeutically-relevant natural products when subjected to careful scrutiny. In summary, the novel insights brought about by this study will be of general interest to readers of Nature, and will form the basis for developing new antibiotic leads to counter multi-drug resistant pathogens.

The study is elegantly conducted, conclusions are fully justified and well supported by a series of complementary

experiments. Data and replicates are well presented, and statistical aspects well documented. I therefore fully recommend that this manuscript be considered for publication and only have the following minor comments:

p. 4 – A couple of lines describing the “Wright Actinomycetes Collection (WAC) library” and what is special about the overall approach that avoids rediscovery of existing compounds would be a useful addition to the introduction.

p. 4 – For the uninitiated, please explain what “Global Natural Products Social (GNPS) molecular networking analysis of the mass spectral signatures” is.

p. 5 – “The stereochemistry predicted based on the presence or absence of epimerase domains, accurately aligned with experimental data for all ten amino acids (Supplementary Fig. 10).” - It seems that the reference should be to Suppl. Fig. 9.

p. 7 – The interaction with U387 is mentioned in the text, but not shown in Fig. 3.

p. 8 – “The overlap in the binding site of MKM with the CCA end of the E-site tRNA (Fig. 3i)” should refer to Fig. 3h.

p. 8 – Although it is clear from panel 3h that the 3’ end of E-tRNA and MKM overlap, how this impedes translocation is less obvious and should be discussed within the context of the established bacterial translocation mechanism. A schematic showing how the drug interferes with this process within the context of the whole ribosome would make this easier to grasp for readers outside the ribosome field.

p. 9 and Fig. 4 – Are the amino acid-specific effects observed codon-specific as well? In addition to looking at enriched/depleted amino acid motifs, the authors should also analyze their Ribo-Seq data from a codon-centric perspective.

p. 9 – “This result suggests that the ribosome translating through threonine codons is more impervious to antibiotic action than at other codons.” I find it difficult to rationalize this mechanistically, because T depletion is observed over many codons, including the A-site. Perhaps the authors could comment further on this aspect?

p. 9 – “In contrast, despite the presence of MKM, a fraction of ribosomes was able to move through the Thr codons of the yrbA-TTT mRNA (Fig. 4e, right gel)”. How do the author explain the stall on Thr2 in the presence of 5 uM MKM? Based on Ribo-Seq data, are drug-bound ribosomes expected to bypass all Thr codons with equal ease?

p. 27 – The second paragraph of section “Cryo-EM data processing” should be merged with the preceding paragraph as it repeats some of the information.

Fig. 3g – The residue labeled as His9 is in fact D-Phe3.

Fig. 5e – Showing the y-axis using a log2 scale might be more appropriate in this case. Also, why are methylated nucleoside ions other than C depleted in the ManE-expressing cells?

Extended Data Figure 1 – Please show the WAC 7405 chromatography profile.

Supp. Fig. 11 – “Bayesian” is misspelled.

Supp. Fig. 12 – Please provide model-map cc curves.

Supp. Fig. 13a – Please label and add outlines to 30S and 50S to give a better sense of where the secondary site is located.

Supp. Fig. 15 – Density for distinctive tRNA modifications should be shown as a separate panel. Moreover, modified tRNA bases should also be modelled (they are absent from the PDBs provided).

Supp. Table 2 – Please include the reference to antiSMASH since the annotations are derived from it.

Supp. Table 3 – Why are the full-length sbmA, topA and rpmI genes 3 nucleotides longer than in the corresponding Uniprot entries? Adding the accession codes to the table would be helpful.

Referee #4

(Remarks to the Author)
A. SUMMARY OF THE KEY RESULTS
Kaur and colleagues report the discovery of a novel antibiotic from a well-studied bacterial species. Following the isolation of this antibiotic, manikomycin, they use an extensive array of approaches to determine a detailed understanding of its mechanism of action. These experiments include structural analysis using cryo-EM, activity towards various E. coli mutants, cytotoxicity assays, C. elegans infection experiments, phenotypic analysis of E. coli, transcription/translation assays and associated toeprinting analysis and ribosome profiling. Further experiments show that sensitivity to the MKMs is not impacted by resistance mechanisms for other antibiotic classes, which is a promising result. The chemical identification and associated biosynthetic gene cluster analysis is reported very well, where gene cluster cloning confirmed the biosynthetic origin of the manikomycins. Identification of the gene cluster also led to the discovery of a self-resistance mechanism via a

cluster-encoded ribosomal methyltransferase. Overall, the manuscript is very well-written and presented.

B. ORIGINALITY AND SIGNIFICANCE
Overall, this represents an impressive study that provides a benchmark for assessing the antimicrobial potential and mechanism of action of newly discovered antibiotics. The identification of a new target for an antibacterial molecule is an exciting result, while the fact that this molecule comes from a well-studied bacterial species makes this work an ideal example of how novel antibiotic candidates have been overlooked for decades. Experimentally, the study uses well-established methodology, so the originality and significance is instead provided by the combination of approaches used to provide a detailed insight into mechanism of action and resistance that could previously take decades to achieve.

C. DATA & METHODOLOGY: VALIDITY OF APPROACH, QUALITY OF DATA, QUALITY OF PRESENTATION
The methodology and standard of data is mostly excellent, with a few points that are raised below.

D. APPROPRIATE USE OF STATISTICS AND TREATMENT OF UNCERTAINTIES
To the best of my knowledge, the statistical methodology is suitable for this work, although there will be more qualified judges of this than me.

E. CONCLUSIONS: ROBUSTNESS, VALIDITY, RELIABILITY
The conclusions of the manuscript are well-defined and robust.

F. SUGGESTED IMPROVEMENTS

1. A key detail of this study is that the molecule is truly novel, but the only evidence is an analysis using GNPS. GNPS will only show similarity to molecules in its own spectral libraries, which are obviously very limited. The authors should therefore indicate that they confirmed this novelty via structural searches against more comprehensive libraries, such as SciFinder, PubChem, Reaxys or similar. My own quick searches indicate that they are correct in their assertion, but this should be checked.

2. It seems surprising that the majority of the work is carried out with MKM-A when MKM-B and MKM-E are more potent (see Extended Data Table 1). There seems to be some detail here that is overlooked in the manuscript, which prompts multiple questions:
(a) Why did the authors not do more work with MKM-E? Can this be clearly rationalised or discussed in the manuscript?
(b) What is the relative proportion of these molecules during fermentation? This should be shown in a clear chromatogram.
(c) Does this proportion vary depending on the fermentation medium or time? Is there any reason why that peptide bond could be unstable, or do the authors suspect that a peptidase is removing the N-terminal Arg?
(d) Biosynthetically, the C domain in the first module should not be required for the reported molecules. Such "starter C" domains are usually associated with N-terminal acylation in cyclic peptides (e.g. in the biosynthesis of surfactin and some Pseudomonas and Paraburkholderia lipopeptides, e.g. https://www.nature.com/articles/s41467-020-20548-8). Is there any evidence for larger acylated molecules?

3. The experimental data for MKM-A is excellent (detailed NMR, MS/MS and Marfey's, plus structural data from the ribosome complexes), but the manuscript lacks some associated data for MKM-B and MKM-E. The structural proposals shown in Fig. 1 seem rational, but more supporting data should be presented:
(a) Supplementary Fig. 10 shows MS/MS data for MKM-A and indicates that this is "used as a reference for the comparative analysis of other MKM variants", but this comparative analysis isn't shown and should be added. This analysis would presumably provide some clear proposals for MKM-C and MKM-D, which are described as octopeptides, but no other information is presented beyond HR-MS.
(b) Supplementary Fig. 10 also shows high-resolution mass spectra for each variant but there's no indication whether these masses are within error for the proposed structures.
(c) MKM-B and MKM-E were purified in reasonable amounts as they were used in multiple MIC assays, along with Marfey's analysis, but there is no corresponding NMR data. Ideally, the NMR data should be presented. In the absence of NMR data, it is appropriate to refer to these as "proposed structures" or similar.

MINOR CORRECTIONS
Line 100 and reference 9: antiSMASH database is cited, but a more accurate citation is for the paper(s) that describe antiSMASH itself.

Lines 110-111: "6.7 kb" is an error. In addition, the original paper describing M1154 should be cited. Finally, it would be informative to highlight how the BGC was cloned, given there are multiple options for this step. This detail is in the methods, but a few words here would be informative.

Line 283: It would be useful to refer to the table that shows the mycobacteria data.

Line 295: the data in Fig. 6c does not appear to show "complete eradication" - could this be better phrased ("X-fold reduction" or similar), or is there control data to show that the low cfu after 6 hours is equivalent to an uninfected control?

Lines 323-324: "its Stachelhaus motif (a key region of protein)" could be phrased with more accuracy, such as "its Stachelhaus motif, which is a set of residues that confer substrate specificity to adenylation domains", or similar.

Lines 325-326: “such flexibility in adenylation domains has been observed previously”. One very specific example is mentioned here, but there are multiple other examples. Consider citing a few examples or editing the text to “has been observed previously, such as in the PheATE module”. Otherwise, is it possible that an arginase instead converts Arg to Orn in MKM biosynthesis?

Lines 464-465: “Yield varied in different batches of fermentation”. It would be useful to specify the range of yields that were obtained. Is this a molecule produced in sub mg/L quantities, or is it more abundant?

Figure 1: His9 is incorrectly drawn with a D- stereocentre. Has the correct stereochemistry been used when fitting the molecule to the cryo-EM density? Also, the drawing of the bond angles for Phe3 could be improved. The data shown for expression in S. coelicolor is reasonable, but some further proof via a supplementary figure would be ideal (exact mass and matching MS/MS data, which the authors likely have already obtained).

Figure 4: It may be clear to someone familiar with the assay, but could some more detail be included in the legend description of panel a?

Extended Data Fig. 4: The legend should clearly describe what is being measured for the shaded scale. This is eventually described in Ext. Data Fig. 7, but should be included here first.

Supplementary Fig. 9: For clarity, the figure legend could say that Asn converts to Asp under acid hydrolysis, hence why the comparison is made with Asp standards.

Supplementary Fig. 17: It would be useful to mention and cite the informatic tool that was used to identify these residues.

G REFERENCES: APPROPRIATE CREDIT TO PREVIOUS WORK?
The manuscript is appropriately cited, with a few suggested additions recommended above.

H. CLARITY AND CONTEXT
The manuscript is easy to read and well-structured. The comments above highlight a few sections where some further clarity could be provided to ensure all relevant data is presented and discussed clearly.

Version 1:

Reviewer comments:

Referee #1

(Remarks to the Author)
The authors have satisfactorily replied to most queries, but the question regarding the potential of manikomycin as drug remains unresolved. By convention, and based on both the Industry practice and recent publications in this Journal, a potential clinical candidate is a compound that shows mouse efficacy, typically thigh or lung infection. Apart from this, other criteria are potency, a reasonably low frequency of resistance, and the expectation of safety. Low in vivo stability of MKM A precluded an efficacy study. The authors note in the rebuttal that the compound does not cause acute toxicity in mice, but this is hard to evaluate given the low exposure of compound due to instability. A particular concern, which the authors acknowledge, is the polycationic nature of this series. Polycationic peptides have a common liability, nephrotoxicity. Most of the studies, including ex vivo and in vivo, were conducted with MKM-A (MIC 4-8 µg/ml) that has a charge of +5. The more active MKM-E (MIC 0.5-2 µg/ml) is +6, so activity increases with charge. It is not clear that a medicinal chemistry optimization program will be able to produce a potent lead that is not a polycation. In the rebuttal, the authors cite aminoglycosides and polymyxin as existing polycationic antibiotics. While this is a valid point, nephrotoxicity limits the use of these compounds, and the introduction of a novel semisynthetic aminoglycoside plazomicin that is largely free of preexisting resistance met with market failure, primarily due to concerns regarding nephrotoxicity. Compounding this, another polycation, murepavadin, failed in Phase III due to nephrotoxicity. Not clear that there is appetite for developing a polycationic antibiotic.

These considerations notwithstanding, the discovery of manikomycin is exciting, and the detailed understanding of its novel mode of action targeting the ribosomal E site not only broadens our understanding of antimicrobials, but is likely to inform rational development of compounds that may result in a therapeutic, even if it is not based on the MKM series per se.

Minor:
Not clear why most experiments were performed with the less active MKM-A. Throughout the manuscript, the authors use the generic term “MKM” without specifying which manikomycin analogue is employed in individual experiments (in the results section), which is problematic given the substantial potency differences. From the methods section, it appears that the full MKM series was used for MIC assays, hemolytic activity, and mammalian cytotoxicity tests. Both MKM-A and MKM-E were used for cell-free transcription/translation studies; but only MKM-A was used for outer membrane permeability tests, resistance studies, ribosome profiling, ex vivo, and in vivo studies. It is not clearly specified which MKM analogue was purified through heterologous expression, used for toeprinting analysis, treatment of cells imaged by SEM, or structural analysis.

Referee #2

(Remarks to the Author)
I co-reviewed this manuscript with one of the reviewers who provided the listed reports.

Referee #3

(Remarks to the Author)
The authors have satisfactorily addressed all points raised by myself and the other referees, and I congratulate them on their exciting and high-quality study. I have no further comments and recommend that the manuscript be considered for publication.

Referee #4

(Remarks to the Author)
The authors have addressed all referee comments very well, with additional data added along with suitable changes to the associated text. In addition, I think the authors' comments to referee 1 regarding the relevance of the molecule and its potency are appropriate and well supported in their response, as well as via edits to the manuscript.

One minor additional detail is that the legend of Extended Data Fig. 6 could be better phrased to describe the contents of the figure. For example, panels g-h are described as: "g-h, comparison of the archaeal/H. marismortui 70S ribosome bound with (g) 13-deoxytedanolide (purple, PDB ID 2OTJ) alone, or (h) overlayed with MKM (blue)."

However, h (along with b, d and f) instead seems to show structures of MKM bound to the E. coli ribosome, which is not discusses at all in the figure legend. Based on the existing figure legend, I expected panel h to show the MKM structure overlayed onto the H. marismortui 70S ribosome.

Open Access This Peer Review File is licensed under a Creative Commons Attribution 4.0 International License, which permits use, sharing, adaptation, distribution and reproduction in any medium or format, as long as you give appropriate credit to the original author(s) and the source, provide a link to the Creative Commons license, and indicate if changes were made.
In cases where reviewers are anonymous, credit should be given to 'Anonymous Referee' and the source.
The images or other third party material in this Peer Review File are included in the article's Creative Commons license, unless indicated otherwise in a credit line to the material. If material is not included in the article's Creative Commons license and your intended use is not permitted by statutory regulation or exceeds the permitted use, you will need to obtain permission directly from the copyright holder.
To view a copy of this license, visit https://creativecommons.org/licenses/by/4.0/

Referee #1 (Remarks to the Author):

The authors report the discovery of a new antimicrobial depsipeptide, manikomycin (MKM), from Streptomyces rimosus, with selective activity against Enterobacteriaceae. Resistance mapping identified the inner membrane transporter SbmA as essential for compound uptake, explaining some selectivity in its action. The resistance studies also implicated the ribosome as the target. The highlight of the study is the structural analysis performed on MKM's interaction with stalled ribosomes using toeprinting and cryo-EM, which shows that MKM binds the large ribosomal subunit, blocking the movement of deacylated tRNA into the E site. This is consistent with resistance mutations and in vitro translation assays, which confirmed the mechanism of action. Self-resistance in the Streptomyces producer was also explained by identifying ManE, an rRNA methyltransferase, which prevents MKM's binding to the ribosome by a posttranscriptional modification.

The MKM's mechanism of action is novel for bacteria, and in this regard, it is an interesting study. The compound itself however is not a realistic drug candidate. Potency is moderate, and MIC tests were not performed in the presence of serum, so it is not clear how much of the activity will be diminished by protein binding; frequency of resistance due to null mutations in transport is high, 10-7; spectrum of action limited to E. coli and Klebsiella; the compound is a polycation, likely to cause nephrotoxicity; stability in vivo is poor, precluding mammalian study.

Response to Reviewer 1.

We thank Reviewer 1 for his high opinion about our study and we respect his thoughts on the clinical prospects of MKM. However, we would like to clarify several points regarding his comments.

1. The compound itself however is not a realistic drug candidate.

While we agree with the reviewer that MKM in its current form may not be an immediate clinical candidate, it is important to remember that this is true for most natural bioactive compounds that typically serve as prototypes for clinically relevant semisynthetic drugs. The unprecedented mode of MKM action and the novelty of its chemical scaffold make it an exciting starting point for further development, given its unique mode of action and structure. We note that the top-ranked priority by the WHO is Carbapenem-resistant *Klebsiella pneumoniae*, which is sensitive to MKM, as demonstrated in our manuscript. The clinical promise of the MKM scaffold is reinforced by its unique binding to the E-site of the bacterial ribosome, which makes it impervious to existing target-based resistance mechanisms in pathogens. Additionally, its origin from a well-known *Streptomyces*

strain effectively counters claims that these organisms are an exhausted source of new chemistry. For these reasons, we view MKM as a significant, if not groundbreaking, innovation that could open new avenues in drug discovery and ribosome research, supporting its publication in *Nature*.

2. Potency is moderate, and MIC tests were not performed in the presence of serum, so it is not clear how much of the activity will be diminished by protein binding.

We respectfully disagree with the reviewer's assessment of MKM's potency. Emphasizing once again that we are presenting an unoptimized natural compound, we find its potency quite notable. We have demonstrated strong sub- and single-digit MIC values for the MKM-E variant: <0.5 μg/mL against *E. coli*, 1-2 μg/mL against *Klebsiella*, and 1 μg/mL against *Salmonella*, all measured in RPMI-1640 medium, which is increasingly recognized as a more physiologically relevant medium for MIC testing (e.g., Miller, Sarah, et al., 2023 *Antimicrobial Agents and Chemotherapy* 67.1 (2023): e01320-22; Luna, B. et al., Nat. Microbiol. 5, 1134–1143 (2020)). Additionally, MKM showed excellent activity against *Mycobacterium tuberculosis*, with an MIC of 0.25 μg/mL, further supporting its potential.

Our *ex vivo* blood model experiments provide compelling evidence of MKM's stability in body fluids. We observed a significant reduction in viable counts of MDR *Klebsiella* clinical isolates within 6 hours of treatment in human blood, indicating effectiveness in a complex biological matrix and largely addressing the serum protein binding concerns (Figure 6(c)). Additionally, direct stability assays conducted in both human and mouse blood plasma confirmed that MKM remains stable under these conditions (Supplementary Fig. 35 a,b).

Furthermore, Table A shows that serum does not affect MKM's MIC, suggesting that protein binding is not an issue (see below).

Table A. Minimum inhibitory concentrations (MIC) of strains with and without serum, tested with MKM-A

Strain	MIC (RPMI) μg/ml	MIC (RPMI+10% Human serum) μ/ml	Fold change
E. coli C0617	8	8	1
E. coli C1508	4	4	1
K. pneumoniae C1559	8	8	1
K. pneumoniae C1459	8	8	1

3. frequency of resistance due to null mutations in transport is high, 10-7; spectrum of action limited to E. coli and Klebsiella;

We probably did not make it sufficiently clear that the 10^{-7} frequency of resistance was observed only when we used an artificial, antibiotic-hypersusceptible *E. coli* strain (SQ110ΔtolC::pZsbmA), which has been engineered to carry a single ribosomal RNA operon. This experiment was designed to investigate the site of action and the resistance mechanism in a sensitized background, not to determine the actual intrinsic resistance rates.

The actual resistance frequencies for wild-type strains are significantly lower: 3.7×10^{-10} for *E. coli* BW25113 and 1.1×10^{-8} for *Klebsiella* C1559. Serial passaging required to obtain significantly resistant mutants and sequenced genomes revealed multiple mutations. These rates are very favorable for a drug scaffold, consistent with those of clinically successful antibiotics. It was our oversight during manuscript editing not to include these wild-type data in the results section, despite mentioning the experiment in the methods section. These data have been added to the Results section.

Furthermore, we demonstrated that, unlike some other antibiotics (such as certain antimicrobial peptides), which depend on a single transporter to enter the cell, MKM utilizes multiple transporters. As a result, mutations in just one transporter led to only low-level resistance.

Regarding the spectrum, MKM is strategically focused on *Enterobacteriaceae*, including *E. coli, Klebsiella*, and *Salmonella*, with potent activity against multidrug-resistant clinical isolates (e.g., carbapenem-resistant *Enterobacteriaceae*). MKM is also active against mycobacteria. This targeted spectrum is a strength, not a limitation, as these pathogens are classified as "critical" priorities by the WHO's global priority list because of their role in severe infections like urinary tract infections (UTIs), sepsis, and hospital-acquired pneumonia, with CRE alone causing 13,100 cases and 1,100 deaths annually in the U.S. (CDC, 2019 Antibiotic Resistance Threats Report). In addition, *in vitro* studies on a panel of human microbiome strains (Extended Data Table 1) show that MKM does not inhibit the growth of beneficial gut flora, potentially avoiding *Clostridioides difficile*-associated complications, a major concern with broad-spectrum antibiotics.

4. the compound is a polycation, likely to cause nephrotoxicity; stability in vivo is poor, precluding mammalian study.

While MKM's polycationic nature raises legitimate concerns about nephrotoxicity, we note that many successful drugs have overcome similar challenges through optimization of the compound, dosing, and formulation. This establishes a precedent that toxicity risks can be managed or minimized through structural modifications. Notably, several important antibiotics (e.g., aminoglycosides, polymyxins) are also polycationic. We also want to emphasize that MKM is well-tolerated in mice, although we did not include these data in the original manuscript. Animal studies showed that mice tolerated doses of up to 220 mg/kg (divided into four doses over 20 hours) via the subcutaneous route without showing signs of acute toxicity. While, in a mouse neutropenic thigh infection model, we did not consistently observe a statistically significant reduction in CFUs in tissue, the lack of efficacy in these preliminary *in vivo* studies likely reflects pharmacokinetic limitations rather than an inherent failure of the compound, as supported by our compelling ex vivo blood activity results (Figure 6(c)).

To further explore this hypothesis, we conducted a more detailed pharmacokinetic evaluation in uninfected mice. *In vitro* PD revealed excellent stability in mouse and human plasma (Supplementary Fig 35 a, b). However, MKM-A exhibited sub-optimal peak plasma concentration (C_{max} 9.13 µg/ml) and rapid systemic elimination with a terminal half-life of ~36 minutes following a single SC dose of 50 mg/kg (Supplementary Fig 35 c). These results suggest that insufficient plasma exposure, rather than inherent pharmacological inactivity, may underlie the inconclusive efficacy findings. These results are now included in the manuscript. Follow-up studies will focus on medicinal chemistry to improve pharmacological properties.

In conclusion, although the reviewer's concerns raise valid questions about the future of MKM per se as a drug candidate, which will require further research efforts to address, we believe that the novelty of being the first antibiotic targeting the bacterial ribosome E-site and our comprehensive microbiological, biochemical, and structural data outweigh the inherent limitations of this first-in-class prototype. Detailed SAR studies using medicinal chemistry to improve PK properties are the next crucial steps, and we are actively pursuing them, although they are beyond the scope of our report.

Referee #2 (Remarks to the Author):

I co-reviewed this manuscript with one of the reviewers who provided the listed reports.

Referee #3 (Remarks to the Author):

Kaur et al. report the identification and characterization of manikomycin (MKM), a novel antibiotic that inhibits bacterial mRNA-tRNA translocation through specific targeting of the ribosomal P site. MKM is active against multi-drug-resistant Enterobacteriaceae, displays low toxicity against human cells, and is not affected by known resistance mechanisms, making it an excellent candidate for future drug development. Remarkably, MKM was identified from a known antibiotic producer, Streptomyces rimosus, thanks to an improved fractionation approach. The importance of this work therefore extends beyond the discovery of a new, promising antibiotic by showing that careful analysis of previously analyzed soil organisms can still yield novel, therapeutically-relevant natural products when subjected to careful scrutiny. In summary, the novel insights brought about by this study will be of general interest to readers of Nature, and will form the basis for developing new antibiotic leads to counter multi-drug resistant pathogens.

We thank the reviewer for appreciating and emphasizing the key points that make our work innovative and exciting.

The study is elegantly conducted, conclusions are fully justified and well supported by a series of complementary experiments. Data and replicates are well presented, and statistical aspects well documented. I therefore fully recommend that this manuscript be considered for publication and only have the following minor comments:

p. 4 – A couple of lines describing the “Wright Actinomycetes Collection (WAC) library” and what is special about the overall approach that avoids rediscovery of existing compounds would be a useful addition to the introduction.

This information is added to the text

p. 4 – For the uninitiated, please explain what “Global Natural Products Social (GNPS) molecular networking analysis of the mass spectral signatures” is.

A brief explanation is included

p. 5 – “The stereochemistry predicted based on the presence or absence of epimerase domains, accurately aligned with experimental data for all ten amino acids (Supplementary Fig. 10).” - It seems that the reference should be to Suppl. Fig. 9.

This has been corrected

p. 7 – The interaction with U387 is mentioned in the text, but not shown in Fig. 3.

Panel e in Fig. 3 has now been updated to include U387.

p. 8 – “The overlap in the binding site of MKM with the CCA end of the E-site tRNA (Fig. 3i)” should refer to Fig. 3h.

This has been corrected.

p. 8 – Although it is clear from panel 3h that the 3’ end of E-tRNA and MKM overlap, how this impedes translocation is less obvious and should be discussed within the context of the established bacterial translocation mechanism. A schematic showing how the drug interferes with this process within the context of the whole ribosome would make this easier to grasp for readers outside the ribosome field.

We added a panel to Figure 4 showing the overall scheme of translocation (Fig. 4a) and clarified in the text and the figure legend why binding of MKM in the E site would interfere with this process.

p. 9 and Fig. 4 – Are the amino acid-specific effects observed codon-specific as well? In addition to looking at enriched/depleted amino acid motifs, the authors should also analyze their Ribo-Seq data from a codon-centric perspective.

In response to the reviewer’s question, we analyzed the enrichment of specific codons at the sites of MKM-induced arrest. In neither of the A, P, or E sites did we observe any significant preference for specific Thr codons. We show these data as an additional panel in the Extended Data Fig. 5 (panel b) and refer to them in the Discussion section of the revised manuscript.

p. 9 – “This result suggests that the ribosome translating through threonine codons is more impervious to antibiotic action than at other codons.” I find it difficult to rationalize this mechanistically, because T depletion is observed over many codons, including the A-site. Perhaps the authors could comment further on this aspect?

Please see clarification of this point below after the next comment.

p. 9 – “In contrast, despite the presence of MKM, a fraction of ribosomes was able to move through the Thr codons of the yrbA-TTT mRNA (Fig. 4e, right gel)”. How do the author explain the stall on Thr2 in the presence of 5 uM MKM? Based on Ribo-Seq data, are drug-bound ribosomes expected to bypass all Thr codons with equal ease?

In the original version of the manuscript, we wrote that Thr codons were “excluded” at the sites of MKM-induced arrest and that the ribosome translating through these codons was “impervious” to the antibiotic. This was an incorrect term and in the revised version we replaced ‘excluded’ with ‘depleted’ and eliminated the term ‘impervious’. In fact, MKM does arrest the ribosome at all codons but with varying efficiency. For unknown reasons, the arrest, while still happening, is less efficient when the ribosome translates through Thr codons. Therefore, it is not surprising that during in vitro translation in the presence of MKM, the ribosome stalls at the Thr codons that are proximal to the start codon of the ORF as observed in our

toeprinting experiments (Fig. 4f). In the MKM-treated cells, the ribosome traverses Thr codons more efficiently than other codons until it gets arrested at the subsequent codons more conducive to MKM action. This would explain the reduced presence of Thr codons located upstream from the sites of the drug-induced arrest observed in the ribosome profiling data. To clarify these points, we now write in the Results section: "I*nterestingly, the strongest effect was depletion of threonine codons at the sites of MKM-induced arrest and over several preceding codons (Fig 4e, Extended Data Fig. 5b). This result suggests that arrest at the threonine codons is the least efficient compared to that at other codons*." We further write in Discussion "*Faster E-site tRNA dissociation, slow accommodation of the A-site tRNA, or slow peptide bond formation would provide a better opportunity for MKM binding in the vacant E site. Conversely, longer occupancy of the E site by the deacylated tRNA or preferential antibiotic displacement by certain tRNAs during formation of the ribosome hybrid state (Fig. 4a) would make MKM-induced ribosome stalling less likely. Reduced obstruction of ribosome transit through such codons prior to the drug-induced ribosome arrest could possibly account for the underrepresntation of threonine codons at and prior to the site of the drug-induced translation arrest (Fig. 4b). Further investigations can be carried out to elucidate which structural properties of tRNA^{Thr} could account for such an effect."*

p. 27 – The second paragraph of section "Cryo-EM data processing" should be merged with the preceding paragraph as it repeats some of the information.

We merged the two paragraphs in the cryo-EM data processing section as requested.

Fig. 3g – The residue labeled as His9 is in fact D-Phe3.

We have now corrected the label in Fig 3g to Phe3.

Fig. 5e – Showing the y-axis using a log2 scale might be more appropriate in this case. Also, why are methylated nucleoside ions other than C depleted in the ManE-expressing cells?

We believe that in this case, using the linear scale reveals more vividly the magnitude of the effect. As to why some methylated nucleoside ions are slightly depleted, there could be several possible explanations. For example, ManE maybe acting at the intermediate steps of the ribosome assembly modulating the progression of the ribosome through the assembly process and affecting activity of other rRNA modifying enzymes. Alternatively, overexpression of the MKM resistance methyltransferase may negatively affect expression of some other cellular proteins, including housekeeping rRNA modifying enzymes. Because we do not have a definite explanation and given the very small magnitude of the effect, we prefer to leave this discussion out of the paper not to distract the reader from the main message.

Extended Data Figure 1 – Please show the WAC 7405 chromatography profile.

The chromatogram profile has been added

Supp. Fig. 11 – "Bayesian" is misspelled

Thank you for spotting this spelling mistake. This has now been corrected.

Supp. Fig. 12 – Please provide model-map cc curves.

We have now provided model-map cc curves in Supp. Fig. 12 (now Supp.Fig 27) (new panels e and f) as requested.

Supp. Fig. 13a – Please label and add outlines to 30S and 50S to give a better sense of where the secondary site is located.

There appears to be a misunderstanding here since the image is only the 50S, therefore, we cannot add an outline for the 30S. Instead, we have added an additional, more familiar "crown" view of the 50S, and then indicated how it relates to the enlarged view that best visualizes the drug bound in its secondary site at the back of the 50S subunit.

Supp. Fig. 15 – Density for distinctive tRNA modifications should be shown as a separate panel. Moreover, modified tRNA bases should also be modelled (they are absent from the PDBs provided).

Unfortunately, the local resolution of the tRNAs is not sufficient to observe the modifications, presumably due to flexibility in the tRNAs, hence their absence from the deposited models. We were also hoping to use the modifications as support for the assignment of the tRNAs, however, this is not possible. therefore, the best support for the tRNA assignment comes from biochemistry, coupled with the codon-anticodon interactions, which we have shown in Fig. 15. One exception is the small bit of additional density for the msi^{2}A modification at position A37 of the A-site tRNA, which is also consistent with our assignment of tRNA^{Phe} in this position (panel a below) and not tRNA^{Leu}, which lacks this modification (panel b below. However, given the lack of density for the majority of the modification, we also did not feel that it was appropriate to include this modification in the models, therefore, we have left the modifications out of the current models.

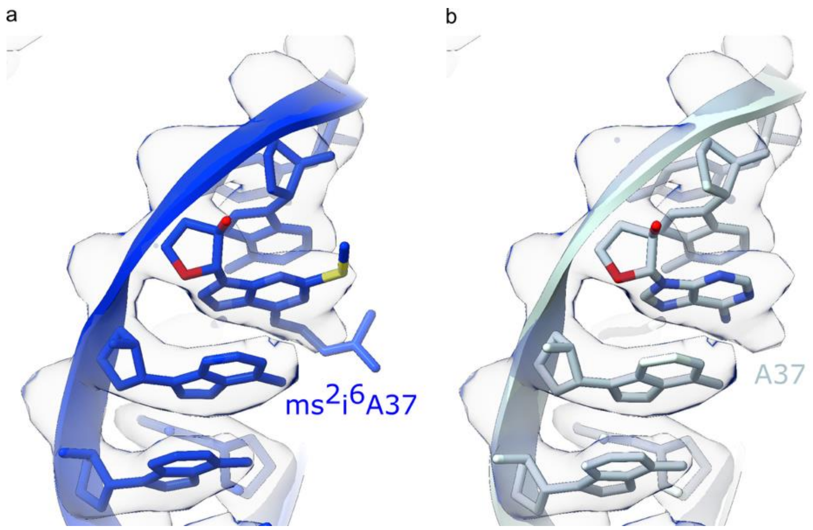

Supp. Table 2 – Please include the reference to antiSMASH since the annotations are derived from it.

This has been included

Supp. Table 3 – Why are the full-length sbmA, topA and rpmI genes 3 nucleotides longer than in the corresponding Uniprot entries? Adding the accession codes to the table would be helpful.

Uniprot entries do not take into account the stop codon of the gene; this accounts for the three-nucleotide difference in the gene length. The accession numbers to the relevant gene sequences have been added to the table.

Referee #4 (Remarks to the Author):

A. SUMMARY OF THE KEY RESULTS

Kaur and colleagues report the discovery of a novel antibiotic from a well-studied bacterial species. Following the isolation of this antibiotic, manikomycin, they use an extensive array of approaches to determine a detailed understanding of its mechanism of action. These experiments include structural analysis using cryo-EM, activity towards various E. coli mutants, cytotoxicity assays, C. elegans infection experiments, phenotypic analysis of E. coli, transcription/translation assays and associated toeprinting analysis and ribosome profiling. Further experiments show that sensitivity to the MKMs is not impacted by resistance mechanisms for other antibiotic classes, which is a promising result. The chemical identification and associated biosynthetic gene cluster analysis is reported very well, where gene cluster cloning confirmed the biosynthetic origin of the manikomycins. Identification of the gene cluster also led to the discovery of a self-resistance mechanism via a cluster-encoded ribosomal methyltransferase. Overall, the manuscript is very well-written and presented.

We thank the reviewer for the favorable evaluation of our study.

B. ORIGINALITY AND SIGNIFICANCE

Overall, this represents an impressive study that provides a benchmark for assessing the antimicrobial potential and mechanism of action of newly discovered antibiotics. The identification of a new target for an antibacterial molecule is an exciting result, while the fact that this molecule comes from a well-studied bacterial species makes this work an ideal example of how novel antibiotic candidates have been overlooked for decades. Experimentally, the study uses well-established methodology, so the originality and significance is instead provided by the combination of approaches used to provide a detailed insight into mechanism of action and resistance that could previously take decades to achieve.

C. DATA & METHODOLOGY: VALIDITY OF APPROACH, QUALITY OF DATA, QUALITY OF PRESENTATION

The methodology and standard of data is mostly excellent, with a few points that are raised below.

D. APPROPRIATE USE OF STATISTICS AND TREATMENT OF UNCERTAINTIES

To the best of my knowledge, the statistical methodology is suitable for this work, although there will be more qualified judges of this than me.

E. CONCLUSIONS: ROBUSTNESS, VALIDITY, RELIABILITY

The conclusions of the manuscript are well-defined and robust.

F. SUGGESTED IMPROVEMENTS

1. A key detail of this study is that the molecule is truly novel, but the only evidence is an analysis using GNPS. GNPS will only show similarity to molecules in its own spectral libraries, which are obviously very limited. The authors should therefore indicate that they confirmed this novelty via structural searches against more comprehensive libraries, such as SciFinder, PubChem, Reaxys or similar. My own quick searches indicate that they are correct in their assertion, but this should be checked.

While GNPS provides spectral similarity within its curated libraries, we agree that broader chemical databases are necessary to establish structural novelty. Accordingly, we conducted structural searches in SciFinder and PubChem, which confirmed that the MKM-A scaffold is not present in the current chemical literature. This supports our conclusion that the molecule is novel. We have updated the text with this information.

2. It seems surprising that the majority of the work is carried out with MKM-A when MKM-B and MKM-E are more potent (see Extended Data Table 1). There seems to be some detail here that is overlooked in the manuscript, which prompts multiple questions:

(a) Why did the authors not do more work with MKM-E? Can this be clearly rationalised or discussed in the manuscript?

MKM-E was obtained in much lower yields during fermentation and purification, which limited the amount available for downstream assays, especially those requiring larger quantities (e.g., in vivo and mode-of-action studies). To maintain consistency across all experiments, we proceeded with MKM-A, even though its MIC is comparatively higher.

(b) What is the relative proportion of these molecules during fermentation? This should be shown in a clear chromatogram.

MKM-A is the major product, comprising the dominant peak in the chromatogram and yielding approximately 20 mg/L. **MKM-B is a medium-level product, produced at** ~**8–10 mg/L, less than half of MKM-A. MKM-E is a minor product**, produced at **only ~1–2 mg/L**, **representing less than 10% of MKM-A's yield**. **The small peak area of MKM-E in the chromatogram further reflects its low abundance in the crude extract. Due to these limited proportions, we were unable to obtain sufficient MKM-E for downstream assays that require higher compound quantities. To maintain experimental consistency and feasibility, we performed the experiments using MKM-A. We have added this data to the Supplementary Fig 25a,b.**

(c) Does this proportion vary depending on the fermentation medium or time? Is there any reason why that peptide bond could be unstable, or do the authors suspect that a peptidase is removing the N-terminal Arg?

Within the media and growth parameters used in this study, the relative ratios of MKM-A, B, remained consistent. In some batches MKM-E, MKM-C and MKM-D were not detected or only detected at trace levels (Supplementary Fig 25a,b.). Regarding the N-terminal Arg: the peptide bond does not appear intrinsically unstable. There is the presence of peptidase (orf-2), amidohydrolase (orf-1) and Amidase (man3) in the MKM- BGC. We knocked out each of these genes from the cluster and it did not affect the production of variants (Data not included in paper). There might be other nonspecific peptidases that could potentially remove the N-terminal Arg.

(d) Biosynthetically, the C domain in the first module should not be required for the reported molecules. Such "starter C" domains are usually associated with N-terminal acylation in cyclic peptides (e.g. in the biosynthesis of surfactin and some Pseudomonas and Paraburkholderia lipopeptides, e.g. https://www.nature.com/articles/s41467-020-20548-8). Is there any evidence for larger acylated molecules?

We carefully searched for larger acylated derivatives that would be consistent with a "starter C" domain function, including specifically screening for mass shifts corresponding to common fatty acyl groups. No such species were detected in either the crude extract or purified fractions.

3. The experimental data for MKM-A is excellent (detailed NMR, MS/MS and Marfey's, plus structural data from the ribosome complexes), but the manuscript lacks some associated data for MKM-B and MKM-E. The structural proposals shown in Fig. 1 seem rational, but more supporting data should be presented:

(a) Supplementary Fig. 10 shows MS/MS data for MKM-A and indicates that this is "used as a reference for the comparative analysis of other MKM variants", but this comparative analysis isn't shown and should be added. This analysis would presumably provide some clear proposals for MKM-C and MKM-D, which are described as octopeptides, but no other information is presented beyond HR-MS.

We have now included detailed NMR, MS/MS and Marfey's analysis for MKM-B and MKM-E as well (Supplementary Fig. 9-24). For MKM-C and MKM-D, which were available only in very low quantities, the comparative MS allowed us to propose plausible octapeptide sequences

(b) Supplementary Fig. 10 also shows high-resolution mass spectra for each variant but there's no indication whether these masses are within error for the proposed structures.

We have compared HR-MS with the calculated masses. All the detected species fall within the acceptable mass error (<1 ppm) for the proposed molecular formula. This has been added to Supplementary table 4.

(c) MKM-B and MKM-E were purified in reasonable amounts as they were used in multiple MIC assays, along with Marfey's analysis, but there is no corresponding NMR data. Ideally, the NMR data should be presented. In the absence of NMR data, it is appropriate to refer to these as "proposed structures" or similar.

We have now added the NMR data for both MKM-B and MKM-E (Supplementary Fig. 9-24).

MINOR CORRECTIONS

Line 100 and reference 9: antiSMASH database is cited, but a more accurate citation is for the paper(s) that describe antiSMASH itself.

This has been modified.

Lines 110-111: "6.7 kb" is an error. In addition, the original paper describing M1154 should be cited. Finally, it would be informative to highlight how the BGC was cloned, given there are multiple options for this step. This detail is in the methods, but a few words here would be informative.

This has been corrected

Line 283: It would be useful to refer to the table that shows the mycobacteria data.

The table has been referred

Line 295: the data in Fig. 6c does not appear to show “complete eradication” - could this be better phrased (“X-fold reduction” or similar), or is there control data to show that the low cfu after 6 hours is equivalent to an uninfected control?

The statement as been modified.

Lines 323-324: “its Stachelhaus motif (a key region of protein)” could be phrased with more accuracy, such as “its Stachelhaus motif, which is a set of residues that confer substrate specificity to adenylation domains”, or similar.

The statement as been modified.

Lines 325-326: “such flexibility in adenylation domains has been observed previously”. One very specific example is mentioned here, but there are multiple other examples. Consider citing a few examples or editing the text to “has been observed previously, such as in the PheATE module”. Otherwise, is it possible that an arginase instead converts Arg to Orn in MKM biosynthesis?

The statement as been modified.

Lines 464-465: “Yield varied in different batches of fermentation”. It would be useful to specify the range of yields that were obtained. Is this a molecule produced in sub mg/L quantities, or is it more abundant?

This has been included in Supplementary Fig 25.

Figure 1: His9 is incorrectly drawn with a D- stereocentre. Has the correct stereochemistry been used when fitting the molecule to the cryo-EM density?

We thank the reviewer for alerting us to this mistake. Indeed, His9 was incorrectly drawn as D-His and has now been corrected to L-His9 in Fig 1, ED Fig 1 and ED Fig 2 as well as Sup Fig 29. Unfortunately, we had used the wrong chemical structure (with D-His9) for model building; therefore, we have now re-modelled and refined the structure with the correct L-His9 stereochemistry. The difference is very minor (as shown in the image comparison below), but we have nevertheless updated the structural images using the new structure and amended the PDB deposition. We thank the reviewer again for their sharp eyes in noticing this mistake.

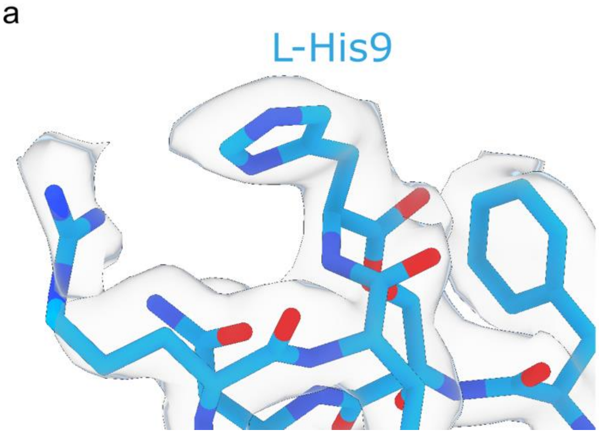

Also, the drawing of the bond angles for Phe3 could be improved. The data shown for expression in S. coelicolor is reasonable, but some further proof via a supplementary figure would be ideal (exact mass and matching MS/MS data, which the authors likely have already obtained).

This has been included in Supplementary Fig 34 b.

Figure 4: It may be clear to someone familiar with the assay, but could some more detail be included in the legend description of panel a?

The limited space allowed for the figure legends does not allow us to elaborate at specifics of the translocation assay (now shown in panel b of the figure). However, we hope that addition of a new panel to the figure (panel a) depicting the key steps of the ribosome translocation would help to better understand the principle of the translocation assay. Furthermore, we provide a detailed explanation of the experimental steps of this assay in the results section.

Extended Data Fig. 4: The legend should clearly describe what is being measured for the shaded scale. This is eventually described in Ext. Data Fig. 7, but should be included here first.

The legend has been modified.

Supplementary Fig. 9: For clarity, the figure legend could say that Asn converts to Asp under acid hydrolysis, hence why the comparison is made with Asp standards.

The statement has been added.

Supplementary Fig. 17: It would be useful to mention and cite the informatic tool that was used to identify these residues.

The bioinformatic tool has been included.

G REFERENCES: APPROPRIATE CREDIT TO PREVIOUS WORK?

The manuscript is appropriately cited, with a few suggested additions recommended above.

H. CLARITY AND CONTEXT

The manuscript is easy to read and well-structured. The comments above highlight a few sections where some further clarity could be provided to ensure all relevant data is presented and discussed clearly.

Referees' comments:

Referee #1 (Remarks to the Author):

The authors have satisfactorily replied to most queries, but the question regarding the potential of manikomycin as drug remains unresolved. By convention, and based on both the Industry practice and recent publications in this Journal, a potential clinical candidate is a compound that shows mouse efficacy, typically thigh or lung infection. Apart from this, other criteria are potency, a reasonably low frequency of resistance, and the expectation of safety. Low in vivo stability of MKM A precluded an efficacy study. The authors note in the rebuttal that the compound does not cause acute toxicity in mice, but this is hard to evaluate given the low exposure of compound due to instability. A particular concern, which the authors acknowledge, is the polycationic nature of this series. Polycationic peptides have a common liability, nephrotoxicity. Most of the studies, including ex vivo and in vivo, were conducted with MKM-A (MIC 4-8 µg/ml) that has a charge of +5. The more active MKM-E (MIC 0.5-2 µg/ml) is +6, so activity increases with charge. It is not clear that a medicinal chemistry optimization program will be able to produce a potent lead that is not a polycation. In the rebuttal, the authors cite aminoglycosides and polymyxin as existing polycationic antibiotics. While this is a valid point, nephrotoxicity limits the use of these compounds, and the introduction of a novel semisynthetic aminoglycoside plazomicin that is largely free of preexisting resistance met with market failure, primarily due to concerns regarding nephrotoxicity. Compounding this, another polycation, murepavadin, failed in Phase III due to nephrotoxicity. Not clear that there is appetite for developing a polycationic antibiotic.

These considerations notwithstanding, the discovery of manikomycin is exciting, and the detailed understanding of its novel mode of action targeting the ribosomal E site not only broadens our understanding of antimicrobials, but is likely to inform rational development of compounds that may result in a therapeutic, even if it is not based on the MKM series per se.

Minor:

Not clear why most experiments were performed with the less active MKM-A. Throughout the manuscript, the authors use the generic term “MKM” without specifying which manikomycin analogue is employed in individual experiments (in the results section), which is problematic given the substantial potency differences. From the methods section, it appears that the full MKM series was used for MIC assays, hemolytic activity, and mammalian cytotoxicity tests. Both MKM-A and MKM-E were used for cell-free transcription/translation studies; but only MKM-A was used for outer membrane permeability tests, resistance studies, ribosome profiling, ex vivo, and in vivo studies. It is not clearly specified which MKM analogue was purified through heterologous expression, used for toeprinting analysis, treatment of cells imaged by SEM, or structural analysis.

Authors' response:

We thank the reviewer for their thoughtful evaluation and feedback throughout the review process. We appreciate their acknowledgment that the discovery of manikomycin and its novel mechanism of action targeting the ribosomal E site represents an exciting contribution to antimicrobial research and our understanding of antibiotic mechanisms.

We agree that in the absence of demonstrable efficacy in mice, it would be inappropriate to position manikomycin as a potential clinical antibiotic candidate. In response, we have already revised the manuscript to avoid strong statements about clinical potential and instead emphasize the discovery of a new chemical scaffold and mechanistic insight into ribosomal E-site targeting. We also acknowledge the reviewer's concerns regarding the liabilities commonly associated with polycationic antibiotics (including nephrotoxicity) and agree that significant medicinal chemistry optimization would be required to address these limitations.

In response to the minor comments, we have clarified throughout the manuscript which specific manikomycin variant was used in each experiment. MKM-A (termed as MKM throughout) was used for all studies unless otherwise specified, primarily due to its availability and initial characterization. In contrast, MKM-E was obtained in much lower yields during fermentation and purification, limiting its use in downstream assays, particularly those requiring larger quantities, such as *in vivo* and mode-of-action studies. To ensure consistency across experiments, we therefore proceeded with MKM-A, despite its comparatively higher MIC.

Referee #2 (Remarks to the Author):

I co-reviewed this manuscript with one of the reviewers who provided the listed reports.

Referee #3 (Remarks to the Author):

The authors have satisfactorily addressed all points raised by myself and the other referees, and I congratulate them on their exciting and high-quality study. I have no further comments and recommend that the manuscript be considered for publication.

Authors' response:

We thank the reviewers for their positive evaluation and kind words regarding our study, and we appreciate their recommendation for publication.

Referee #4 (Remarks to the Author):

The authors have addressed all referee comments very well, with additional data added along with suitable changes to the associated text. In addition, I think the authors' comments to referee 1 regarding the relevance of the molecule and its potency are appropriate and well supported in their response, as well as via edits to the manuscript.

One minor additional detail is that the legend of Extended Data Fig. 6 could be better phrased to describe the contents of the figure. For example, panels g-h are described as: "g-h, comparison of the archaeal/H. marismortui 70S ribosome bound with (g) 13-deoxytedanolide (purple, PDB ID 2OTJ) alone, or (h) overlayed with MKM (blue)."

However, h (along with b, d and f) instead seems to show structures of MKM bound to the E. coli ribosome, which is not discusses at all in the figure legend. Based on the existing figure legend, I expected panel h to show the MKM structure overlayed onto the H. marismortui 70S ribosome.

Authors' response:

We thank the reviewer for their positive assessment and constructive feedback throughout the review process.

We have corrected the legend of Extended Data Figure 6 as suggested to accurately describe the contents of each panel.
